# Sieve analysis of breakthrough HIV-1 sequences in HVTN 505 identifies vaccine pressure targeting the CD4 binding site of Env-gp120

**DOI:** 10.1371/journal.pone.0185959

**Published:** 2017-11-17

**Authors:** Allan C. deCamp, Morgane Rolland, Paul T. Edlefsen, Eric Sanders-Buell, Breana Hall, Craig A. Magaret, Andrew J. Fiore-Gartland, Michal Juraska, Lindsay N. Carpp, Shelly T. Karuna, Meera Bose, Steven LePore, Shana Miller, Annemarie O'Sullivan, Kultida Poltavee, Hongjun Bai, Kalpana Dommaraju, Hong Zhao, Kim Wong, Lennie Chen, Hasan Ahmed, Derrick Goodman, Matthew Z. Tay, Raphael Gottardo, Richard A. Koup, Robert Bailer, John R. Mascola, Barney S. Graham, Mario Roederer, Robert J. O’Connell, Nelson L. Michael, Merlin L. Robb, Elizabeth Adams, Patricia D’Souza, James Kublin, Lawrence Corey, Daniel E. Geraghty, Nicole Frahm, Georgia D. Tomaras, M. Juliana McElrath, Lisa Frenkel, Sheila Styrchak, Sodsai Tovanabutra, Magdalena E. Sobieszczyk, Scott M. Hammer, Jerome H. Kim, James I. Mullins, Peter B. Gilbert

**Affiliations:** 1 Vaccine and Infectious Disease Division and Statistical Center for HIV/AIDS Research and Prevention, Fred Hutchinson Cancer Research Center, Seattle, Washington, United States of America; 2 US Military HIV Research Program, Walter Reed Army Institute of Research, Silver Spring, Maryland, United States of America; 3 The Henry M. Jackson Foundation for the Advancement of Military Medicine, Inc., Bethesda, Maryland, United States of America; 4 Department of Biostatistics, University of Washington, Seattle, Washington, United States of America; 5 Department of Microbiology, University of Washington, Seattle, Washington, United States of America; 6 Department of Biology, Emory University, Atlanta, Georgia, United States of America; 7 Duke Human Vaccine Institute, Duke University, Durham, North Carolina, United States of America; 8 Vaccine Research Center, National Institute of Allergy and Infectious Diseases, National Institutes of Health, Bethesda, Maryland, United States of America; 9 Division of AIDS, National Institute of Allergy and Infectious Diseases, National Institutes of Health, Bethesda, Maryland, United States of America; 10 Clinical Research Division, Fred Hutchinson Cancer Research Center, Seattle, Washington, United States of America; 11 Department of Global Health, University of Washington, Seattle, Washington, United States of America; 12 Department of Medicine, University of Washington, Seattle, Washington, United States of America; 13 Department of Laboratory Medicine, University of Washington, Seattle, Washington, United States of America; 14 Seattle Children’s Research Institute, Seattle, Washington, United States of America; 15 Department of Pediatrics, University of Washington, Seattle, Washington, United States of America; 16 Department of Medicine, Division of Infectious Diseases, Columbia University Medical Center, New York, New York, United States of America; 17 International Vaccine Institute, Seoul, Korea; University of Hong Kong, HONG KONG

## Abstract

Although the HVTN 505 DNA/recombinant adenovirus type 5 vector HIV-1 vaccine trial showed no overall efficacy, analysis of breakthrough HIV-1 sequences in participants can help determine whether vaccine-induced immune responses impacted viruses that caused infection. We analyzed 480 HIV-1 genomes sampled from 27 vaccine and 20 placebo recipients and found that intra-host HIV-1 diversity was significantly lower in vaccine recipients (P ≤ 0.04, Q-values ≤ 0.09) in Gag, Pol, Vif and envelope glycoprotein gp120 (Env-gp120). Furthermore, Env-gp120 sequences from vaccine recipients were significantly more distant from the subtype B vaccine insert than sequences from placebo recipients (P = 0.01, Q-value = 0.12). These vaccine effects were associated with signatures mapping to CD4 binding site and CD4-induced monoclonal antibody footprints. These results suggest either (i) no vaccine efficacy to block acquisition of any viral genotype but vaccine-accelerated Env evolution post-acquisition; or (ii) vaccine efficacy against HIV-1s with Env sequences closest to the vaccine insert combined with increased acquisition due to other factors, potentially including the vaccine vector.

## Introduction

A safe and effective HIV-1 vaccine remains a global priority. Despite improved access to treatment and prevention methods, HIV-1 prevalence and incidence rates remain high in many populations [1–3]. Of the six preventive HIV-1 vaccine efficacy trials to date [4–9], only the RV144 trial showed some efficacy in preventing HIV-1 infections (31% reduction, P = 0.04) [8]. HVTN 505 was a phase 2b efficacy study of a DNA prime/recombinant adenovirus serotype 5 (rAd5) boost HIV-1 vaccine regimen (DNA/rAd5) versus placebo. It enrolled 2,504 HIV-1 negative men and transgender women who had sex with men in the US. The DNA prime vaccine encoded Env from subtypes A, B, and C and subtype B Gag, Pol, and Nef [10, 11]. The rAd5 vector boost expressed Env from subtypes A, B, and C and a subtype B Gag-Pol fusion protein [12]. Each Env construct encoded the surface glycoprotein (gp120) and partial transmembrane glycoprotein (gp41) sequences. Motivated partly by nonhuman primate data [13], the primary goals of HVTN 505 were to examine whether this regimen could prevent HIV-1 infection and/or lower viral load in HIV-1 breakthrough infections. However, the vaccine did not reduce HIV-1 acquisition or viral load in individuals who became infected, where the estimated hazard ratio (vaccine/placebo) of HIV-1 infection was 1.25 (95% confidence interval [CI], 0.71 to 2.21; P = 0.44) [9].

Sieve analysis of breakthrough infections from a preventive vaccine efficacy trial compares HIV-1 sequences from vaccine-experienced and vaccine-naïve participants and allows attribution of vaccine-versus-placebo viral genetic signatures to vaccination [4–9]. These genetic signatures can reflect two types of vaccine pressure: (1) a post-acquisition effect where the vaccine accelerates early HIV-1 evolution before sequences are measured; and (2) a differential vaccine effect on the risk of HIV-1 acquisition, where vaccine efficacy varies depending on the genotype of the exposing HIV-1 [14–23]. The second type is important generally for any vaccine designed to prevent infection with a genetically diverse pathogen such as HIV-1, as the results help define the protective epitopes/loci of the pathogen and can guide next-generation vaccine design. Given the 3-monthly HIV-1 testing schedule in HVTN 505 and the rapid evolution of HIV-1 post-acquisition, it may not be possible to definitively interpret the sieve analysis results as reflecting effect (1) or (2). Nevertheless, if sieve analyses detect vaccine pressure it also identifies locations on the HIV-1 proteome that were potentially targeted by vaccine-induced responses [19–23], and further research may be pursued to discriminate between the two types of effects.

We examined whether the HVTN 505 vaccine exerted pressure on breakthrough viruses, and if this pressure could be attributed to antibody (Ab) or cytotoxic T lymphocyte (CTL) responses. This pressure could manifest itself through a reduced frequency of infections with multiple founder variants in vaccinees compared to placebo recipients, reduced intra-individual diversity of breakthrough sequences in vaccinees compared to placebo recipients, or greater distances of vaccine recipient sequences than placebo recipient sequences from the vaccine insert sequences. The latter two results could be evidenced at different levels of the HIV-1 genome or proteome, such as at whole proteins (“global sieve effects”) or at individual amino acid (AA) sites or short AA stretches (“local sieve effects”). Locally, vaccine-induced pressure exerted through specific Ab or CTL responses could lead to vaccine-versus-placebo differences at antibody contact or allosteric sites, or potential T cell epitopes.

As in the Step and RV144 trials, local sieve effects were found in HVTN 505. In addition, unique to HVTN 505, global sieve effects in Env-gp120 were also observed.

## Materials and methods

### HVTN 505 trial

The HVTN 505 trial (ClinicalTrials.gov Identifier: NCT00865566, start date May 2009) enrolled 2504 Ad5-seronegative, fully circumcised men or transgender women in the US who have sex with men. Other eligibility criteria included being between the ages of 18 and 50 years old, having a history of unprotected anal intercourse with one or more male or male-to-female transgender partners or anal intercourse with two or more male or male-to-female transgender partners in the 6 months before randomization, being HIV-1 and HIV-2 seronegative, and having an alanine aminotransferase level no more than 2.5 times the upper limit of the normal range. A total of 2496 participants were randomized to receive the DNA/rAd5 vaccine (1251 participants) or placebo (1245 participants) at weeks 0, 4, 8, and 24. The original primary objective was to assess the effect of the DNA/rAd5 vaccine regimen on HIV-1 viral load setpoint. After study initiation, the protocol was updated to also assess the efficacy of the DNA/rAd5 vaccine regimen on prevention of HIV-1 acquisition. The final analysis was completed early in April 2013 due to reaching an interim monitoring guideline for lack of vaccine efficacy.

### Ethics statement

All participants gave written informed consent. The study was approved by the institutional review board (IRB) at the Fred Hutchinson Cancer Research Center in Seattle, which acted as a central IRB through agreements with the Annandale, Aurora—University of Colorado Hospital CRS, Atlanta–Hope Clinic, Cleveland, East Midtown, New York–NYBC, New York–Physicians & Surgeons, NYBC–Bronx, Orlando, Philadelphia, San Francisco, and Union Square sites. For the other study sites, the study was approved by local IRBs (the National Institutes of Allergy and Infectious Diseases IRB at the Bethesda site, the Institutional Review Board for Human Use/University of Alabama at Birmingham at the Birmingham site, the Partners Human Research Committee at the Boston (Brigham) site, the Fenway Health Institutional Review Board at the Boston (Fenway) site, the Office for the Protection of Research Subjects at the Chicago site, the University of Texas Southwestern Medical Center Institutional Review Board at the Dallas site, the Fenway Health Institutional Review Board at the Fenway site, the Institutional Review Board of Human Subject Research at the Houston site, the AIDS Research Alliance Institutional Review Board at the Los Angeles site, the Office of the Human Research Protection Program Medical Institutional Review Board at the Los Angeles–Care Center site, the Vanderbilt University Institutional Review Board at the Nashville site, and the University of Rochester Internal Biosafety Committee at the Rochester site).

### HIV-1 vaccine and reference sequences

Vaccine insert and reference sequence accession numbers are given in S1 Supplementary Methods. Notably, the env sequence, in the subtype B Ad5 vector only, had a deletion of the region encoding amino acids 134–191 (HXB2 coordinates), encompassing the V1V2 loop [12].

### HIV-1 sequencing

HIV testing was performed at immunization months 0, 1, 2, 2.5, 6, 7, and 9 and then every 3 months through 24 months. The earliest RNA-positive sample was used for HIV-1 sequencing. Near full-length or overlapping half genome sequences were obtained from plasma samples by limiting dilution PCR and direct sequencing [24]. Sequences were assembled using Sequencher™, version 5.0 (Gene Codes Corporation, Ann Arbor MI), aligned using Geneious (Auckland, NZ) and manually edited; hypermutated sequences were identified using Hypermut [25] and removed before analysis. Sequences have been deposited in Genbank under accession numbers xxx…The alignments can be explored with an interactive web-based visualization and database tool (http://sieve.fredhutch.org/viz) [26].

### Estimated time since the onset of infection

Estimated time since the onset of infection was defined as the mid-point between the last RNA negative and first RNA positive sample, the latter usually being the draw date for HIV-1 sequencing; if the latter was not the draw date for HIV-1 sequencing, the time between the first positive sample and the draw date for HIV-1 sequencing was added onto the estimated time since onset of infection as calculated by the mid-point method described above. Spearman correlation between the estimated time since the onset of infection and mean pairwise diversity adjusted for treatment and multiplicity of founders was computed using the partial.Spearman method of the PResiduals package in R.

### Sequence analysis

All nucleotide sequences from an individual were compared to identify the *mindist*, i.e., the genome sequence closest to the individual’s consensus (near-full-length or two overlapping halves) genome. Nucleotide sequences were translated to amino acid sequences for protein-based analyses. Co-receptor, positive selection evaluation, potential N-linked glycosylation site identification, maximum-likelihood phylogenetic tree reconstruction, and founder multiplicity estimation are described in S1 Supplementary Methods.

### CTL epitope predictions

CTL epitopes were predicted in all breakthrough, vaccine insert, and HIV-1 reference sequences based on each individual’s human leukocyte antigen (HLA) type [27]; see S1 Supplementary Methods for further details.

### Site scanning methods

For all scanning methods, only sites passing variability and alignment quality filters, defined using treatment-blinded data, were included. A site was deemed sufficiently variable for the site scanning methods if 4 or more participants had a sequence matching the reference strain (vaccine insert or B.Anc) and 4 or more participants had a sequence mismatching the reference strain. An alignment quality filter was used to determine sites of sufficient confidence in the multiple sequence alignment. Because highly variable regions are poorly aligned and common descent cannot be established unequivocally, highly variable regions in the signal peptide and envelope variable loops were excluded. The pseudo F (PF) test statistic was used to test for a difference in *mindist* sequence distributions between the vaccine and placebo groups without using a reference sequence. For further details, see S1 Supplementary Methods.

Immunologically relevant AA subsets (e.g., 9-mers or mAb footprints, see S1 Supplementary Methods for more details) were scanned as described [24]. For a given subset of interest, the similarity score was the number of AA matches with the reference subset over all sites. A marginal mean model fit by generalized estimating equations (GEE) was used to compare similarity scores between treatment groups using all breakthrough sequences; this method accounts for within-individual correlation among multiple HIV-1 sequences and bias-corrects the GEE standard errors to account for the limited sample size [28].

### Machine learning sieve analysis

Supervised learning approaches were applied to predict the treatment assignments of participants based on HIV-1 sequence features corresponding to: (a) mAb contact sites in Env; (b) two Env peptide binding hotspots; (c) the V3 region of Env; (d) the combined CD4 binding site (CD4bs) antibody footprints; (e) the gp145 region of Env corresponding to the vaccine inserts; (f) each of the other insert proteins separately (Gag/Pol/Nef); (g) each of the remaining non-insert regions separately (including the region of Env not included in the insert); and (h) all of the proteins combined, insert and non-insert. Machine learning analysis details are given in S1 Supplementary Methods.

### Vaccine efficacy by distance

A “distance-specific hazard ratio” was defined as the ratio (vaccine/placebo) of the incidence of infection with a specific genotype of HIV-1, with genotype defined by the value of a Hamming distance between the aligned *mindist* sequence and the subtype B vaccine sequence [29]. These ratios were defined for Hamming distances based on three subsets of Env-gp120 sites (see Results). Methods for estimating the distance-specific hazard ratio with a 95% confidence interval, and for a Wald test of whether the distance-specific hazard ratio curve varies with distance, are described in [30].

### Multiplicity adjustment

Adjustment for multiple hypothesis testing considered both false discovery rate (FDR) adjustment using the Benjamini-Hochberg procedure [31] and family-wise error rate (FWER) adjustment using the Holm-Bonferroni procedure [32]; multiplicity adjusted P-values were computed using the p.adjust method of the R computing language. FDR adjusted P-values are reported as Q-values. Adjustment was performed for the set of P-values generated by each test. For gene or protein level tests this included one P-value for each HIV-1 gene or protein. For AA site- and set-scanning, multiplicity adjustment was performed separately for each gene/reference or protein/reference combination.

All P-values reported are 2-sided. For hypothesis generation, results with unadjusted P-value < 0.05 are reported, along with Q-values and FWER-adjusted P-values. Results with Q-values < 0.20 were considered statistically significant.

## Results

**Fig 1:**
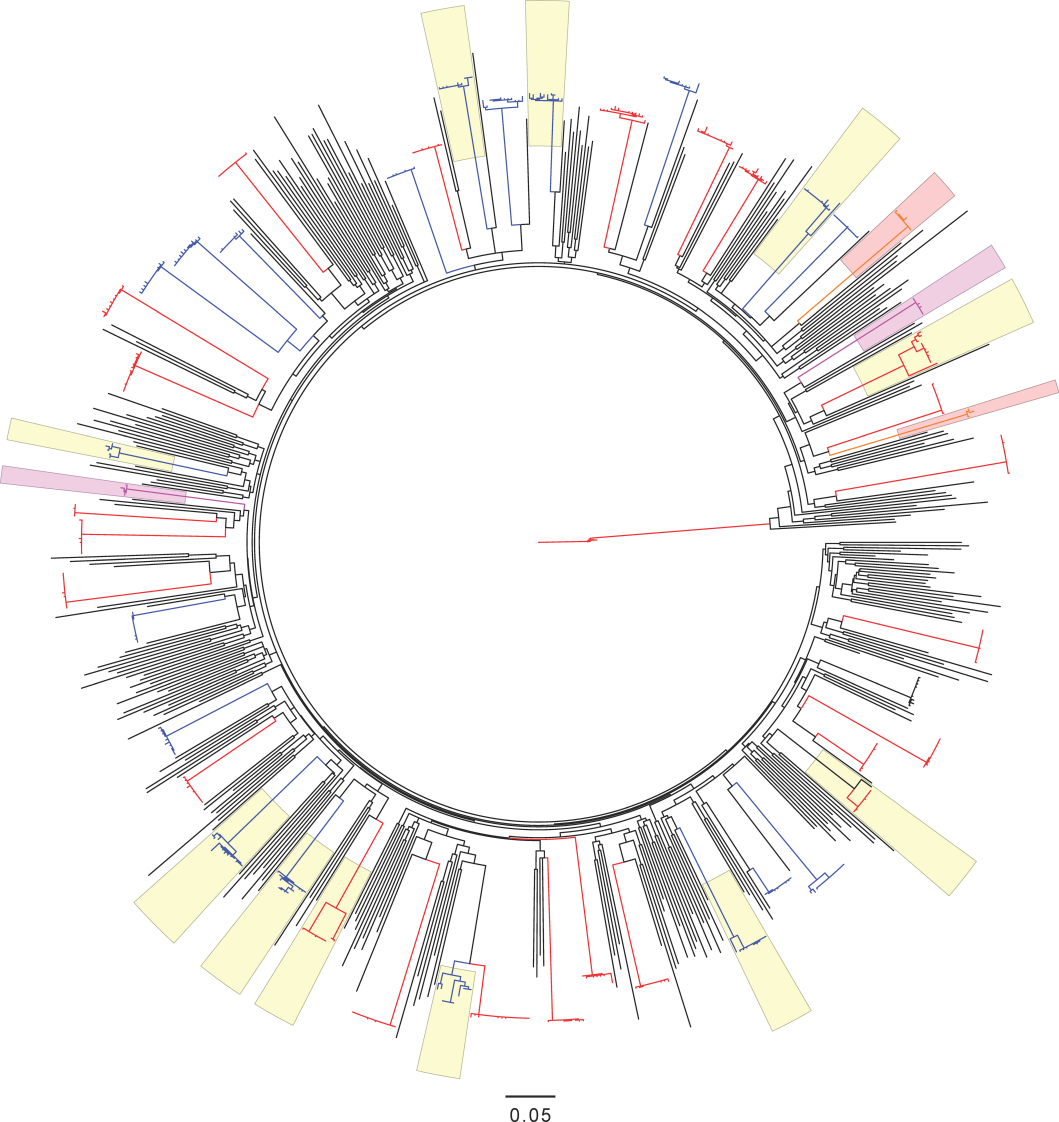
Maximum likelihood phylogenetic tree including 774 *envelope gp120* nucleotide sequences. Sequences from HVTN 505 vaccine (in red) and placebo (in blue) recipients (n = 486) are depicted along with 288 HIV-1 subtype B sequences from the US and sampled since 2005. Sequences from two vaccine recipients who were each dually-infected (with two independent strains) are highlighted with orange and magenta shading. Subjects with multiple founder variants are highlighted with yellow shading.

A total of 480 HIV-1 near-full-length genome equivalents were obtained from 47 of the 48 HIV-1 infected participants (27 vaccine and 20 placebo recipients; insufficient numbers of sequences were obtained from one recipient) from plasma samples collected a median of 90 days after the last negative RNA test. For all but two subjects, plasma samples were collected at HIV-1 diagnosis and 8 participants had not yet seroconverted (S1 Table). All participants were infected with HIV-1 subtype B and all sequences were predicted to use the CCR5 co-receptor alone for entry except for seven of ten sequences from vaccine recipient 505–1569, which were predicted to use both CCR5 and CXCR4 [33, 34]. Phylogenetic analyses showed that this and another vaccine recipient (505–1569 and 505–2227) had each been infected by two unrelated strains − with an average mean pairwise diversity of 11.1%, versus 0.4% among participants infected from a single source partner. Both dual infections were confirmed by sequencing samples collected twelve days later (data not shown). Env-gp120 variable loops did not significantly differ between the vaccine and placebo groups in terms of length, net charge, or potential N-linked glycans. However, there was on average 1 extra cysteine in the vaccine group (mean = 22.6 (vaccine) vs 21.7 (placebo); P = 0.0003), with these AA substitutions found at 12 sites across Env-gp160 (S2 Table, S1 Fig). Phylogenetic analysis of Env-gp120 nucleotide sequences from HVTN 505 participants and independent subtype B sequences (288 US subtype B sequences collected since 2005) showed no obvious vaccine-versus-placebo clustering (Fig 1).

### Global sieve effects: Lower intra-host diversity and greater divergence from the Env-gp120 vaccine insert among vaccine recipient sequences

**Fig 2:**
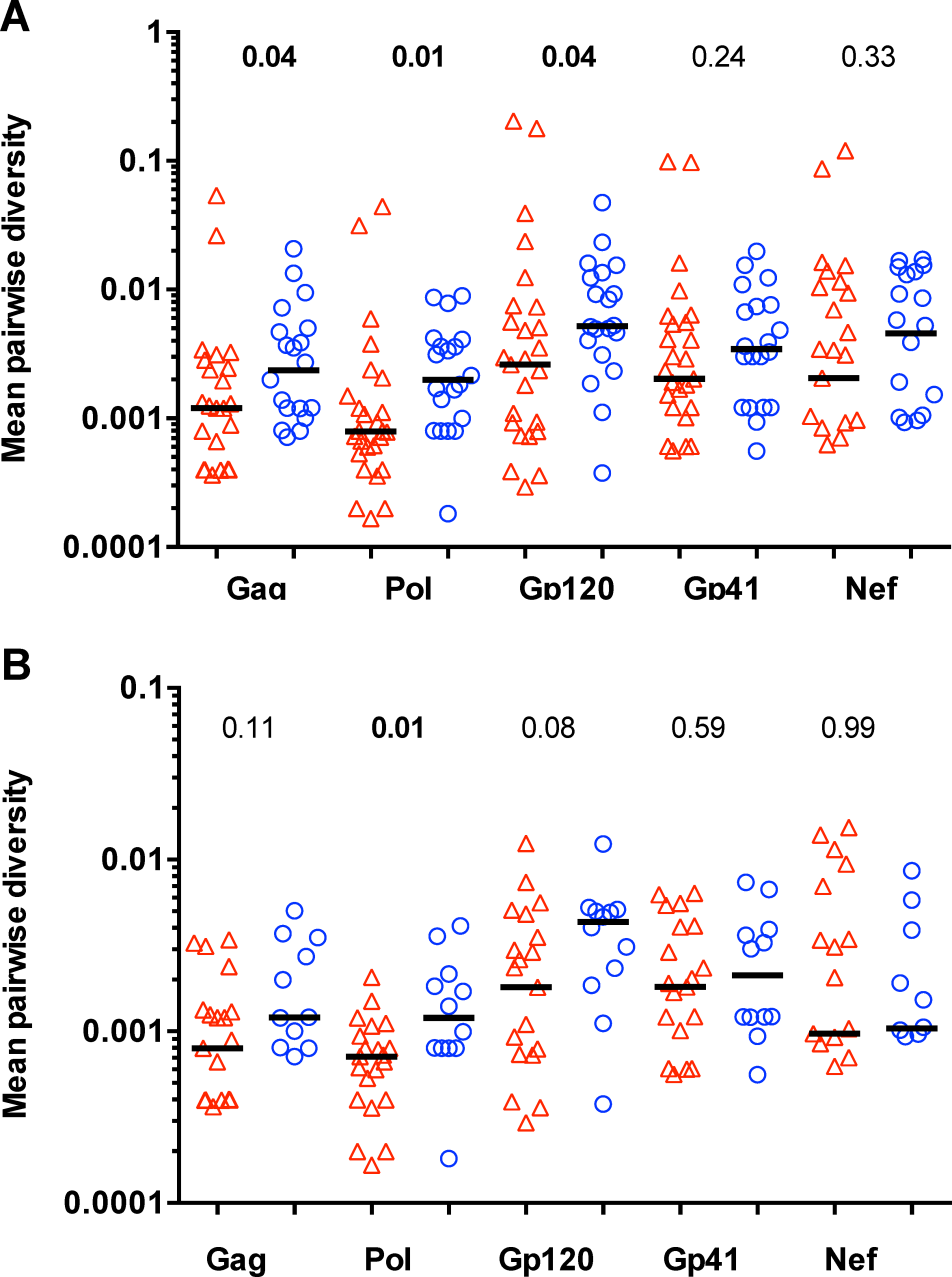
Intra-host mean pairwise diversity among amino acid sequences. Intra-host mean pairwise diversity values were calculated among all the sequences from a given participant separately for each protein corresponding to a vaccine insert, with vaccine recipients in red and placebo recipients in blue. The upper panel included all participants; the lower panel included only participants with single HIV-1 founder viruses. Two-sided Mann-Whitney p-values comparing the vaccine and placebo groups are shown above the panels.

Though not statistically significant, the frequency of infections with multiple founder variants was two-fold lower in the vaccine vs. placebo recipients– 5 of 26 (19%) in the vaccine group compared to 8 of 20 (40%) in the placebo group (Fisher’s exact test, P-value = 0.19; Fig 1). Intra-host amino acid sequence diversity was lower in vaccine recipients than in placebo recipients among Env-gp120, Gag, and Pol sequences (all included in the vaccine) as well as Vif, which was not included in the vaccine (P ≤ 0.04, Q-values ≤ 0.09) (Fig 2). This difference was wider when the two dually-infected vaccine recipients were excluded (P ≤ 0.01) (S3 Table). No significant differences were found in the other HIV-1 proteins, including Env-gp41 and Nef (which were included in the vaccine) and Rev, Tat, Vpr, Vpu. Fig 2 shows that results were comparable whether or not individuals infected with multiple founders were included. No analyses were restricted to the subset of participants with multiple founders as the limited number of subjects and sequences from each founder together with the absence of critical vaccine/placebo patterns segregating the founders precluded formal analysis.

**Fig 3:**
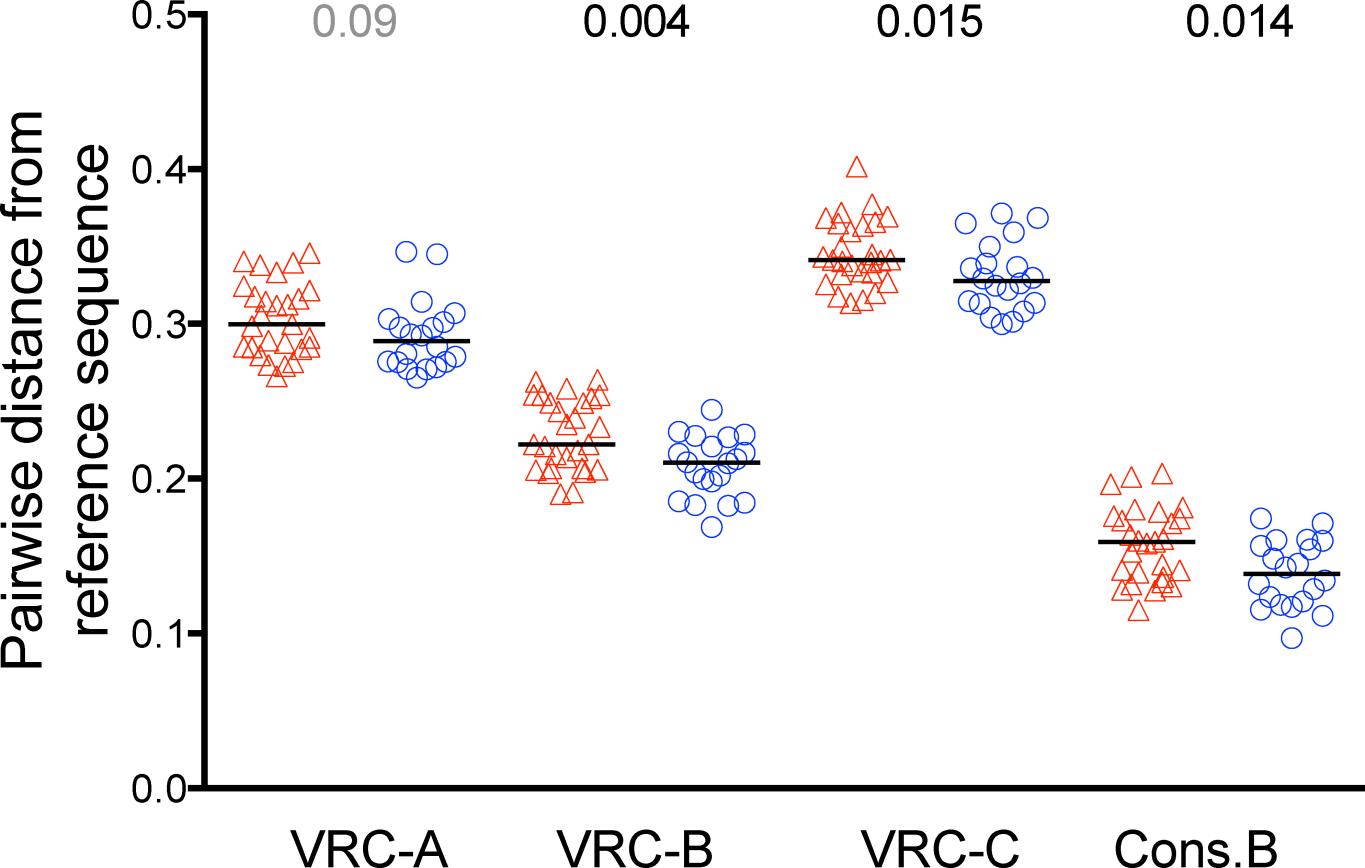
Pairwise distances between breakthrough sequences and each Env vaccine sequence or Env Consensus B. Participant-specific mean distances of breakthrough sequences to the subtype A, B, and C Env vaccine sequences and the Consensus B sequence (labeled VRC-A, VRC-B, VRC-C and Cons.B, respectively). Distances from breakthrough sequences from vaccine recipients are in red and placebo recipients in blue. Distances are based on Env-gp120 sequences with the hypervariable segments deleted (HXB2 positions from signal peptide 10–16; V1 loop 132–152; V2 loop 186–191; V3 loop 309–310; V4 loop 392–413; and V5 loop 460–464). Two-sided Mann-Whitney p-values comparing the vaccine and placebo groups are shown above the panels.

We next assessed vaccine pressure on HIV-1 by comparing pairwise distances of breakthrough sequences from the vaccine insert sequence(s) between the treatment groups. Greater divergence from the vaccine Env-gp120 sequences was observed in vaccine recipients. This difference was significant for the subtype B immunogen–the same subtype as the infecting sequences (mean 0.307 in the vaccine group and 0.285 in the placebo group, P = 0.01, Q-value = 0.12)–and non-significant when compared to the more distant subtype A and C Env-gp120 immunogens (S4 Table). After removing variable segments this difference was smaller in magnitude but slightly more significant (mean 0.222 in the vaccine group and 0.210 in the placebo group, P = 0.004, S6 Table, Fig 3). Similar results were obtained for tree-based distances but were only significant for the subtype B vaccine after removing variable segments (S5 and S6 Tables).

### Local sieve effects: Specific AA sites and linear peptide sequences in breakthrough viruses differentiated treatment groups

Local scanning sieve methods were used to identify the specific AA sites or sets of AA sites underlying the global sieve effect signal found in Env-gp120. First, five AA site-scanning methods [Gilbert, Wu, Jobes (GWJ) [35]; Expected Gilbert, Wu, Jobes (EGWJ) [15]; Model-Based Sieve (MBS) [21]; Physico-Chemical Property (PCP) [36]; Quasi-Earth Mover’s Distance (QEMD) [15] were used to compare each site to the subtype B vaccine insert. Two signature Env-gp120 sites were identified: site 133 was enriched for charged residues among placebo group sequences by PCP (P = 0.00015, Q-value = 0.19) and site 429 showed more mismatches against the vaccine insert in the vaccine group than in the placebo group by MBS (P = 0.0014, Q-value = 0.15) (S7 Table). In addition, analysis of within subject positive selection in Env-gp120 identified eight Env-gp120 sites under positive selection only in the vaccine group: sites 147, 183, 236, 290, 315, 354, 388, and 442 (S8 Table).

A k-mer scanning method was used to identify signature 9-mer peptides in Env-gp120. Twenty-two overlapping 9-mers had significant treatment group differences in their pairwise distance from the subtype B vaccine insert (Table 1). These 9-mers specified six unique linear signature regions corresponding to AA positions 27–37, 86–94, 192–207, 365–373, 425–437, and 464–479 (P < 0.005, Q-values < 0.18) (Table 1; S2, S3, S4, S5, S6 and S7 Figs). Notably, the last four regions overlapped CD4 binding site contact residues [37], all with greater divergences in the vaccine group. Since these 9-mers also corresponded to potential CTL epitopes, we performed sieve analyses of CTL epitope repertoires defined *in silico* based on each participant’s HLA genotype. No significant sieve effects associated with CTL epitopes were found (S9, S10, S11, S12, S13 and S14 Tables; S8 Fig).

**Table 1:** k-mer signature regions.

Protein	Label	HXB2 start	Difference	P-value	Q-value	Adjusted P-value*
Env	a	27	0.97	0.0043	0.18	1
		28	0.93	0.0052	0.18	1
		29	0.87	0.0037	0.18	1
	b	86	-0.56	0.0052	0.18	1
	c	192	0.64	0.0019	0.18	1
		193	0.86	0.0001	0.04	0.04
		194	0.66	0.0034	0.18	1
		195	0.64	0.0032	0.18	1
		199	0.4	0.006	0.18	1
	d	365	0.67	0.0029	0.18	1
	e	425	0.56	0.0038	0.18	1
		426	0.58	0.0042	0.18	1
		427	0.46	0.0038	0.18	1
		428	0.46	0.0038	0.18	1
		429	0.48	0.0022	0.18	1
	f	464	0.56	0.0046	0.18	1
		465	0.61	0.0025	0.18	1
		466	0.79	0.0005	0.11	0.3
		467	0.78	0.0005	0.11	0.32
		469	0.76	0.0065	0.19	1
		470	0.79	0.0059	0.18	1
		471	0.79	0.0058	0.18	1
Pol	a	230	-0.4	0.001	0.17	0.98
		231	-0.4	0.0012	0.17	1
		232	-0.4	0.0012	0.17	1
		235	-0.42	0.0015	0.17	1
		236	-0.42	0.0015	0.17	1
		237	-0.44	0.0012	0.17	1
		238	-0.44	0.0011	0.17	1
	b	353	0.49	0.0014	0.17	1
	c	867	0.34	0.0017	0.17	1
		868	0.33	0.002	0.17	1
		869	0.33	0.002	0.17	1

**Fig 4:**
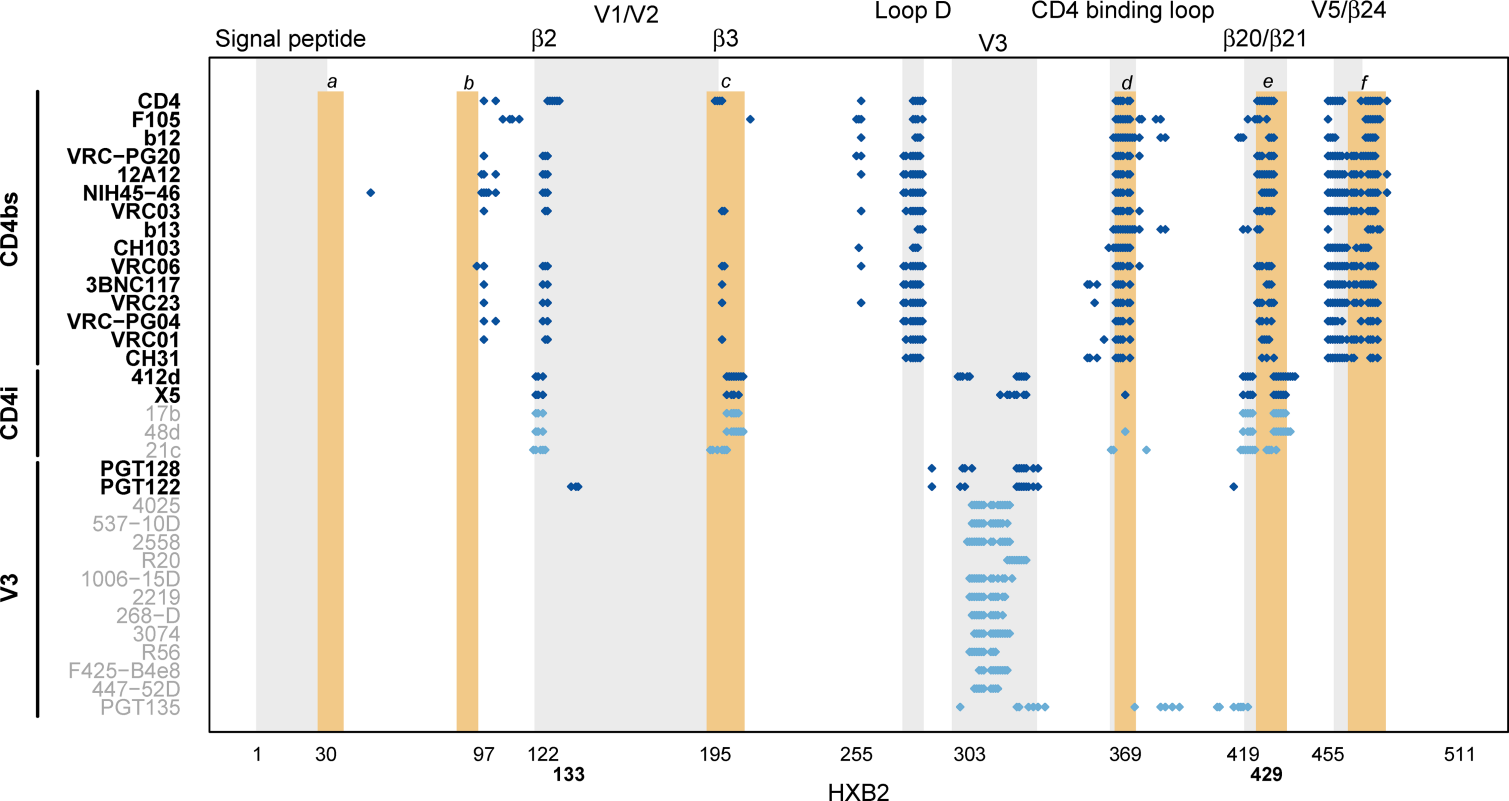
Overlap between HIV-1 Env-gp120 k-mer, AA site and mAb footprint signatures identified with different scanning methods. Positive sieve effects, i.e., greater divergences of sequences from vaccine recipients than from placebo recipients from the vaccine strain, were detected for all 34 CD4bs, CD4i and V3 footprints (S15 Table). The figure represents signatures detected with a Q-value ≤ 0.2 across Env-gp120 (gray bands identify the HIV-1 features labeled at the top of the figure): six k-mer regions are shown as orange bands (labeled a-f as in Table 1); two individual sites, 133 and 429, are labeled in bold on the x-axis; and the 19 of 34 CD4bs, CD4i, and V3 mAb footprints with a Q-value ≤ 0.2 are indicated by dark blue points and labeled on the y-axis in bold font. The 15 mAb footprints that are not statistically significant signatures are indicated by light blue points and labeled on the y-axis in gray. Footprint site sets were grouped by mAb class (CD4bs, CD4i, and V3) and ranked within class by P-value. Footprint site sets for mAb classes where no significant effect was identified are not shown (Quarternary, Env-gp41, and Glycan; see S15 Table).

**Fig 5:**
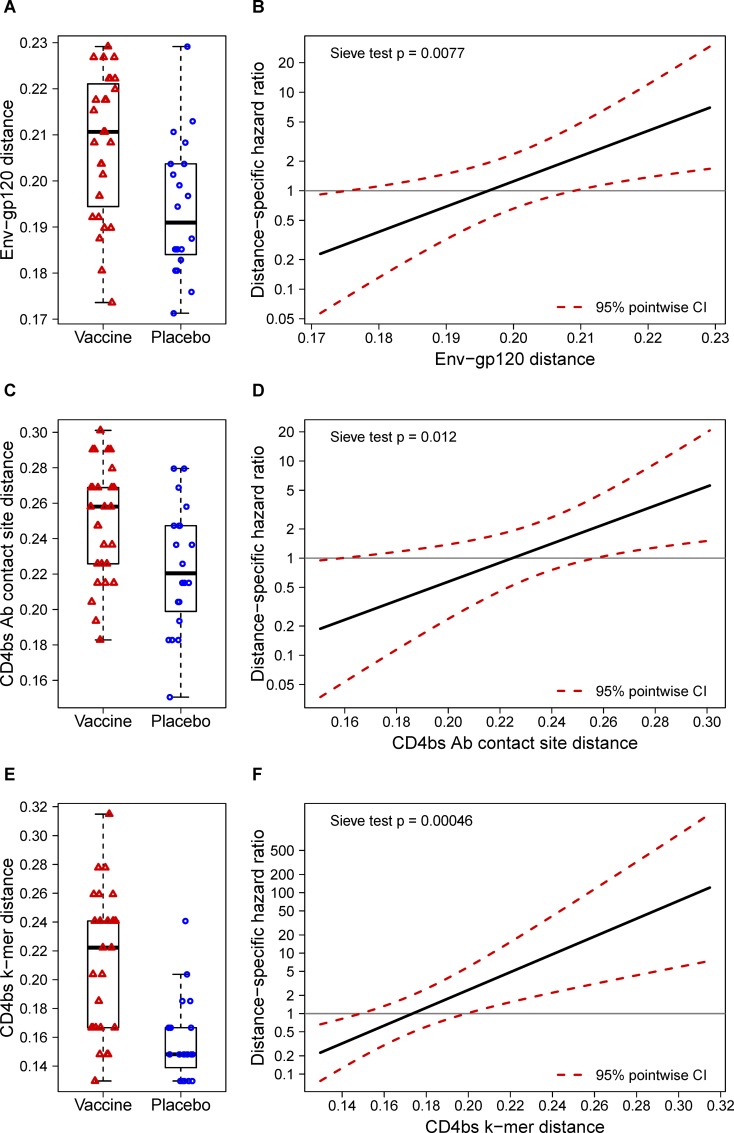
Distance-specific vaccine efficacy. Boxplots of hamming distances of *mindist* sequences to the subtype B vaccine strain by treatment group for three sets of Env-gp120 sites: **(A)** all Env-gp120 sites that could be aligned with confidence (n = 432); **(C)** the 93 CD4bs antibody contact sites; and **(E)** 54 sites corresponding to the 4 k-mers overlapping the CD4bs where significant sieve effects were found (Table 1). The mid-line of the box denotes the median and the ends of the box denote the 25^th^ and 75^th^ percentiles. The whiskers that extend from the top and bottom of the box extend to the most extreme data points that are no more than 1.5 times the interquartile range or if no value meets this criterion, to the data extremes. **(B, D, F)** Distance-specific hazard ratio based on the corresponding distances shown in panels A, C, and E, respectively. The solid black lines show the estimated hazard ratio as a function of distance, and the dashed red lines represent a 95% point-wise confidence interval (CI) around that estimate. The ‘Sieve test’ 2-sided p-value reports the result of the test of [30] for whether the hazard ratio varies with distance.

Six k-mers in Env (labeled a-f as in Figs 4 and 5) and three k-mers in Pol (labeled a-c) with a sieve effect Q-value ≤ 0.20. HBX2 start is the first position of each 9-mer. Difference corresponds to the estimated mean difference in per sequence Hamming distance to the subtype B vaccine strain between the vaccine vs. placebo group breakthrough sequences; a positive (negative) value indicates greater (lesser) distance to the vaccine strain in the vaccine group sequences.

*Family wise error rate adjusted by the Holm-Bonferroni procedure done separately for each gene.

Finally, the same k-mer scanning method was used to evaluate 44 sets of known mAb contact sites [15], 19 of which showed significant vaccine/placebo differences (Fig 4; S15 Table). CD4 binding site (CD4bs) antibodies bind to conserved epitopes in gp120 near the CD4-binding site and can compete with the CD4 receptor for binding to gp120 [38], whereas CD4-induced (CD4i) antibodies bind to conserved epitopes on the inner domain of gp120 that are normally hidden but are exposed by CD4 binding [39, 40]. All CD4bs (n = 15) mAb footprints and two of the five CD4i mAb footprints had significantly greater divergences from the subtype B vaccine insert in the vaccine group, as did 2 of the 14 V3 mAb contact site sets (PGT128 and PGT122, Q-value ≤ 0.2). The other 3 CD4i footprints and 12 V3 mAb contact site sets also had greater divergences from the vaccine insert in the vaccine group, although these signatures were not statistically significant. There was no evidence for a sieve effect in the remaining mAb classes (Quaternary-, Env-gp41-, and Glycan-targeted; P-values > 0.3) (S15 Table). Application of the phylogenetic divergence analysis presented above to the mAb contact site sets found significantly larger tree-based distances in the vaccine group for the combined CD4bs contact sites (mean 0.305 in the vaccine group and 0.271 in the placebo group, P = 0.041; P = 0.075 for pairwise distances) and no significant results for other subsets of mAb contact sites (CD4i-, V3-, Quaternary-, Env-gp41-, and Glycan-targeted; P ≥ 0.16). Since a previous analysis determined that antibodies induced by the DNA/rAd5 vaccine regimen are not broadly neutralizing [41], we investigated the antibody-dependent cellular phagocytosis (ADCP) activity of antibodies from participants in the HVTN505 trial. As shown in S9 Fig, the DNA/rAd5 vaccine regimen induced positive ADCP responses to ConSgp140-coated beads in an estimated 92.5% of vaccinees at 4 weeks post-final vaccination.

Site-specific and k-mer scanning methods were also used to test for AA distribution differences between treatment groups in the remainder of the HIV-1 proteome. The reference sequence for comparisons was the subtype B vaccine insert sequence for Gag, Pol, and Nef and the Ancestral B (B.Anc) sequence for proteins not included in the vaccine (Rev, Tat, Vif, Vpr, and Vpu). Three of the site-specific scanning methods identified Pol 238 as a signature site (GWJ, EGWJ, and QEMD; P = 0.0006, 0.0002, and 0.0006, respectively; Q-values = 0.11, 0.05, and 0.11, respectively; S7 Table). The k-mer scanning methods identified 11 9-mers, all in Pol, that significantly differed between the vaccine and placebo groups when compared to the subtype B vaccine insert (Table 1). After accounting for 9-mer overlap, three unique Pol linear signature regions remained: 230–246, 353–361, and 867–877 (P < 0.005, Q-values < 0.17) (Table 1). The presence of the Pol 238 signature site in the 230–246 linear signature region may explain the identification of this linear signature region. When site subsets identified in previous sieve analyses of the Step and RV144 trials were assessed in the HVTN 505 sequences, no sites showed significant treatment group differences (GWJ: P ≥ 0.18; EGWJ: P ≥ 0.10). The pseudo F test found significant sequence differences only for Vif (P = 0.002, FWER-adjusted P = 0.02). Machine learning analyses considering Gag, Pol, and Nef were not able to classify sequences by vaccine vs. placebo group above the pre-set performance threshold [cross-validated area under the receiver operating characteristic (ROC) curve (AUC) value > 0.7] (S16 Table).

### Env-gp120 vaccine similarity associated with genotype-specific vaccine efficacy

While the HVTN 505 trial had no overall vaccine efficacy, it is possible that vaccine efficacy calculated separately against different HIV-1 genotypes was positive for some genotypes (vaccine benefit to lower HIV-1 risk) and negative (vaccine detriment to increase HIV-1 risk) for other genotypes. Both global and local sieve effects were found in Env-gp120, raising the hypothesis that the vaccine preferentially blocked infection of HIV-1 Env-gp120 variants more similar to the vaccine sequence. A hazard ratio (vaccine/placebo) of HIV-1 infection was estimated by stratifying breakthrough sequences based on their distance to the subtype B insert calculated for three sets of Env-gp120 sites: i) all Env-gp120 sites aligned with confidence (n = 432, 64.6% of sites); ii) CD4bs antibody contact sites (n = 93); and iii) sites corresponding to the 4 linear signature regions overlapping the CD4bs where significant sieve effects were found (n = 54; Table 1). The hazard ratio significantly increased with the distance to the subtype B insert for each set (Env-gp120: P = 0.0077; CD4bs antibody contact sites, P = 0.012; CD4bs-related linear signature regions, P = 0.00046; Fig 5). The estimated hazard ratio was approximately 0.20 for HIV-1 sequences that were most similar to the subtype B vaccine insert (which corresponds to 80% vaccine efficacy against these sequences), approximately 1.0 for HIV-1 sequences with intermediate distances (zero vaccine efficacy), and exceeded 2.0 (doubling of risk by vaccination) for HIV-1 sequences that were most divergent from the vaccine (Fig 5). If the measured Env-gp120 distances reflect the distances of the transmitted viruses (which cannot be empirically verified from HVTN 505 data as noted in the Introduction), then these findings suggest that the vaccine may have selectively blocked HIV-1 infection when Env-gp120, especially in the CD4bs region, was close to the vaccine sequence.

## Discussion

**Fig 6:**
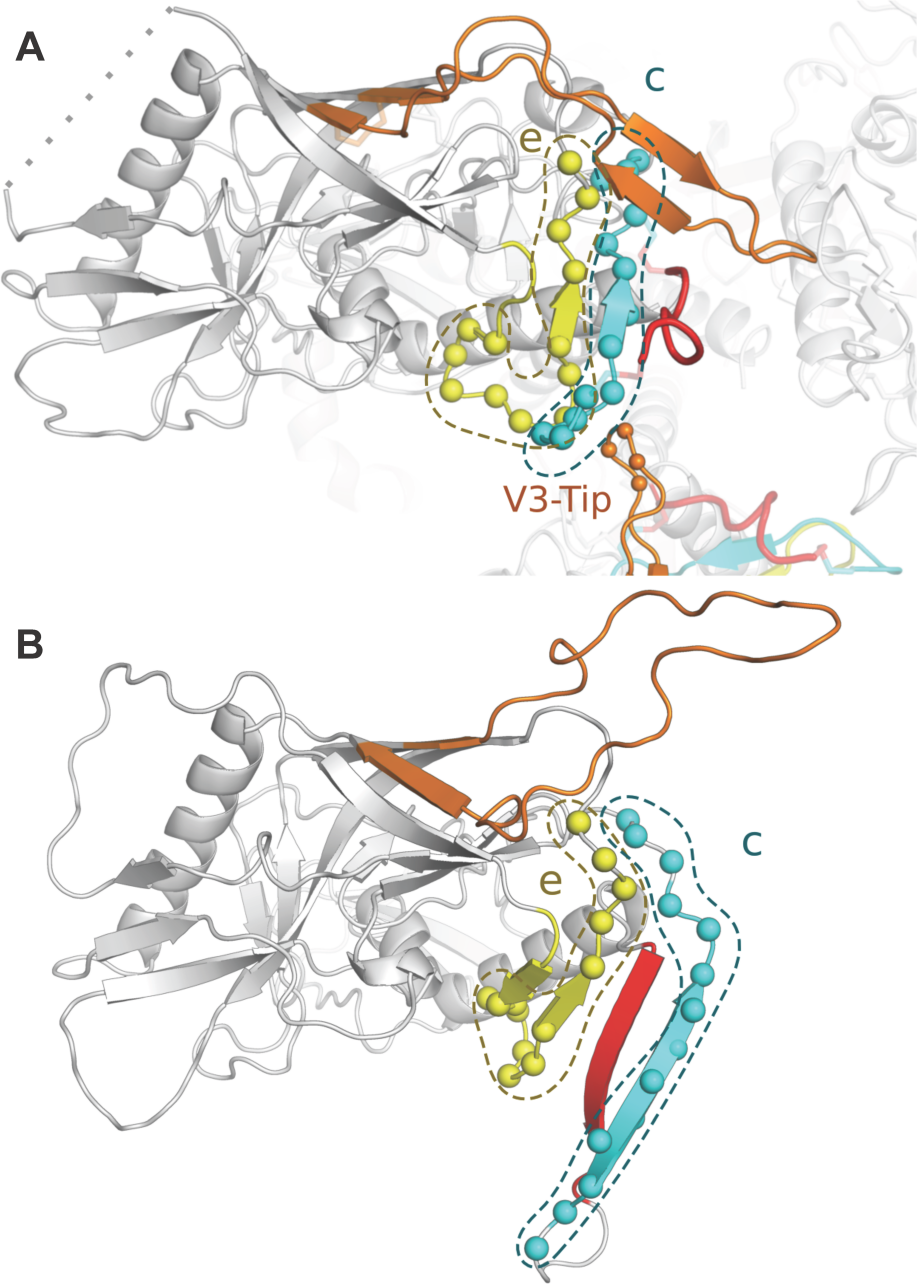
Involvement of two signature k-mers within gp120 in the formation of the bridging sheet and exposure of the V3 loop. **(A)** The pre-fusion closed state (structure 4TVP [45]) and **(B)** the CD4-bound state (structure 2B4C [46]). The V3 loops from two gp120 monomers are in orange and 4 spheres represent its tip (GPGQ/R residues 312–315). The two signature segments are shown in turquoise (AA192-207) and in yellow (AA425-437) labeled c and e, respectively, as in Table 1 and Fig 4. Segments c and e map to the bridging sheet corresponding to sites AA119-126, AA196-205, and AA422-436. In the CD4-bound state, the bridging sheet is formed and the V3 loop is released and becomes more accessible to V3 antibodies.

We analyzed on average 10 full-length genome sequences per individual from 47 participants (27 vaccine and 20 placebo recipients) infected with HIV-1 during the HVTN 505 vaccine efficacy trial with samples obtained a median 90 days since the last HIV-1 RNA negative time point. Similar Sanger sequencing strategies have demonstrated the utility of this approach to studying early HIV-1 infections [15, 24, 42–44]. We found both global and local sieve effects of vaccination in HVTN 505, whereas previous studies found no effect (Phambili) or only local effects (Step and RV144 trials). The global sieve effects corresponded to significantly lower Env-gp120 intra-host diversity and greater divergence from the subtype B Env-gp120 vaccine insert in vaccine group sequences compared to placebo group sequences. The local sieve effects suggested antibody-mediated sieving rather than pressure on CTL epitope repertoires, since signatures mapped to defined CD4bs-, CD4i-, and V3-antibody footprints but not to predicted CTL epitopes in each individual. Furthermore, 4 of the 6 linear signature regions identified overlapped the CD4bs, including two (AA 192–207 and AA 425–437) that overlapped two of the beta strands forming part of the bridging sheet (β3 and β21) (Fig 6). In addition, local sieve effects were seen for all 15 CD4bs mAb contact site footprints and for 2 of the 5 CD4i antibody footprints. Since CD4 binding triggers bridging sheet formation, these signatures could affect the conformational transition between the pre-fusion closed and CD4-bound states (Fig 6).

Since the sieve effects were found predominantly in Env-gp120, our findings most likely reflect selective pressure on the breakthrough viruses in the vaccine group that is focused on regions of Env-gp120 that are functionally relevant to viral infectivity. The local signatures suggest antibody-mediated immune pressure. Considering the data presented in S9 Fig, ADCP focused on Env-gp120 could have exerted selective pressure on the breakthrough viruses in the vaccine group.

However, other evidence suggests that antibodies may not fully explain the Env-gp120 sieve signatures. First, a study by Williams et al. showed that the dominant antibody response induced by this vaccine was to Env-gp41 [47] yet our study found no significant vaccine-versus-placebo differences in Env-gp41. The lack of signatures in Env-gp41 suggests that the dominant antibody response was ineffective, which is consistent with the limited functional response of Env-gp41 antibodies [47]. Further evidence pointing to ineffective antibody responses comes from peptide microarrays that identified two vaccine reactivity hotspots (V3 AA304-318 and Env-gp41 AA581-593) but focused sieve analyses in these two regions did not reveal any sieve signatures. Although we found two V3-antibody footprint signatures, the location of these two footprints did not coincide with the V3 hotspot, thus these signatures may not represent antibody pressure. Immunogenicity analyses of phase 1 and 2 trials of the same VRC DNA/rAd5 vaccine evaluated here revealed β-chemokine-mediated viral inhibition [48] which has the potential to disrupt co-receptor binding and therefore provides an alternative relationship to the sieve signatures.

Several other vaccine-versus-placebo sieve effect signals were detected. While some are expected to be false positive results, most are likely genuine based on passing multiplicity adjustment, although definitive interpretations for the mechanism(s) behind them are still missing. For example, a larger number of cysteines were found in the vaccine versus placebo group sequences. Cysteine count may affect Env trafficking through the endoplasmic reticulum or its proper folding [49] and is potentially related to infectivity. In other examples, strong signatures were found in which sequences from the vaccine group were more likely to match the vaccine than sequences in the placebo group, the opposite of what is typically expected [15]. A signature site with one of the lowest p-values, Pol 238 (P = 0.0002, Holm-Bonferroni FWER-adjusted P = 0.05), showed this pattern, but we have no explanation for a requirement for conservation at this site. Also, a 9-mer signature in C1, AA 86–94 (P = 0.0052, Q-Value = 0.18) was found that overlaps 3 sites within the interface between Env-gp120 and Env-gp41 [50]. One interpretation for these diverse signatures is that HIV-1 may have many different pathways for escaping the pressure induced by this DNA/rAd5 vaccine, with the different signatures potentially corresponding to different escape pathways.

The sieve effect signatures identified here differ from those identified in RV144 [15, 21] and Step [24], which may not be surprising given the different vaccine regimens, study populations, and in the case of RV144, circulating HIV-1 subtypes. In contrast to the Gag-Pol-Nef vaccine tested in Step, both the RV144 and HVTN 505 vaccine regimens included Env protein inserts designed to elicit humoral as well as cellular immune responses; however, non-CTL immune responses (e.g., antibodies or viral inhibition) to Env appeared to dominate since neither regimen showed evidence of marked CTL pressure across the HIV-1 proteome [51] and sieve signatures were found predominantly in Env. The lack of evident HVTN 505 vaccine-induced pressure on CTL epitopes is also consistent with the less frequent and lower CD8+ T cell responses in HVTN 505 [9] compared to the Step study [52], the latter of which did reveal evidence of CTL pressure. Hence, it remains unclear whether it is beneficial to include constituents designed to elicit CTL responses such as Gag, Pol, or Nef along with Env to elicit antibodies; new vaccine modalities may be needed to induce both T cell- and antibody-mediated responses.

Our results showing Env-gp120 sieve effects in HVTN 505 are intriguing as they correspond to the strongest signal identified to date in the four HIV-1 vaccine efficacy trials evaluated (HVTN 505, Step [24], Phambili [44], and RV144 [15, 21]). They also describe what sieve effects that selectively prevent HIV-1 acquisition would theoretically entail among breakthrough sequences, with both lower intra-host diversity and greater divergence from the vaccine in vaccine recipient sequences compared to placebo recipient sequences [53].

Given the lack of overall vaccine efficacy, the vaccine-elicited immune response may have had no effect on acquisition of HIV-1 for any genetic variants, and the sieve effects are due to anamnestic responses to the vaccine that accelerated early HIV-1 evolution, especially in the CD4bs and CD4i mAb contact site footprints. An alternative explanation is that the vaccine did block infections with HIV-1s most similar at these contact sites to the vaccine yet increased risk of acquisition with HIV-1s that were divergent at these sites from the vaccine, leading to an overall lack of efficacy in the trial as a whole. Increased risk of HIV-1 acquisition has been indicated for the Ad5-based vaccine tested in the Step and Phambili trials [6, 54, 55] and we can surmise that protective and antagonistic forces could have influenced the risk of infection after DNA/rAd5 vaccination. Vaccine regimens that activate cellular immunity have been suggested to generate protective responses while also increasing the number of target cells at infection sites [56–58]; in particular, Ad5-specific CD4+ T cells expanded by DNA/rAd5 vaccination have been shown to be highly susceptible to HIV infection [58].

One possible explanation is that in vaccinated individuals, DNA/rAd5 vaccination can provide protection against HIV acquisition if the exposing strain is sufficiently similar to the vaccine strain. In such a scenario, this protection “outweighs” the general vaccine-enhanced risk potentially conferred by some factor related to vaccination (such as vaccine-expanded Ad5-specific CD4+ T cells, as discussed above). In contrast, in the scenario where the exposing HIV strain is sufficiently divergent from the vaccine strain, the vaccine-induced immune responses do not confer protection. This, combined with the potentially increased “starting level” of risk due to, for example, vaccine-expanded Ad5-specific CD4+ T cells, leaves the vaccine recipient at increased risk compared with an unvaccinated individual. The idea that some vaccine-increased risk of HIV-1 infection could have occurred in HVTN 505 was earlier suggested by Janes et al. (2017), who found that CD8+ Env polyfunctionality was a remarkably strong inverse correlate of HIV-1 risk in HVTN 505 vaccine recipients, suggesting that vaccine recipients with low CD8+ Env polyfunctionality could have had higher risk compared to if they had been unvaccinated, whereas those with high CD8+ Env polyfunctionality could have had partial beneficial vaccine-protection [59]. While it is likely that a balance of vaccine-induced protection with vaccine-increased risk would depend jointly on immune responses and on the degree of CD4bs/i genetic match of exposing HIV-1s, the number of HIV-1 infected cases was too small to conduct an analysis seeking to identify this bivariate dependency.

A limitation of our study is that it is not possible to definitively determine whether the observed sieve effects reflect post-acquisition vaccine-induced pressure on HIV-1 evolution or whether they reflect an effect of vaccination to differentially block acquisition with different exposing HIV-1 sequences. HIV-1 sequencing was performed within a few months of HIV-1 infection: the median time from sequencing to the last negative HIV test was 90 days and eight individuals had not yet seroconverted by the time of sequencing. This level of precision about the timing of infection does not allow an inference that the sequences sampled at diagnosis are identical to the viruses which established infection, although it has been shown that HIV-1 evolution is very limited in the first six weeks of infection [42]. Relatedly, the degree of infection timing uncertainty complicates the identification of distinct founder populations. Another limitation of our study is that longitudinal HIV-1 sequence data were not available, thus precluding any analyses of sieve effect durability. Despite these limitations, the strong sieving seen in Env-gp120 warrants further studies as these results could be directly applicable to the design of next-generation candidate vaccines. In addition, it would be of interest to design future studies with objective to experimentally prove that certain amino acid signatures were responsible for beneficial vaccine efficacy and that others were associated with a lack of vaccine efficacy, and for the latter to experimentally test the causes of vaccine-increased risk.

## Meetings

Rolland M*, deCamp A*, Hall BM, Tovanabutra S, McElrath J, Hammer SM, Sobieszczyk ME, Gilbert PB, Kim JH, Mullins JI and the HVTN505 sieve analysis team.

HVTN505 breakthrough sequences showed HIV vaccine‐associated differences in Env‐gp120. CROI, Seattle, WA 2015. *Contributed equally.

deCamp A, Rolland M, Edlefsen P, Sanders‐Buell E, Hall B, Magaret C, Fiore‐Gartland A, Juraska M, Graham B, Roederer M, Michael N, Robb M, McElrath MJ, Tovanabutra S, Sobieszczyk M, Hammer S, Kim J, Mullins J, Gilbert PB and the HVTN505 sieve analysis team. Sieve Pressure toward the CD4 Binding Site of Env in HVTN 505 Breakthrough Infections. HIV Research for Prevention (HIVR4P), Chicago, Illinois (October 2016).

## Supporting information

S1 Table Mean time since infection and antibody testing. Numbers of participants with primary endpoint HIV-1 infection included in the sieve analysis, mean time between HIV-1 infection and sampling for HIV-1 sequencing, and antibody testing.
(PDF)

S2 Table Non-conserved cysteine residue frequencies in mindist sequences. Subtype B viruses typically possess 18 cysteine residues forming 9 disulfide bridges in gp120 and 2 cysteine residues in gp41 forming an additional disulfide bridge for a total of 20 conserved cysteine residues. These residues are also conserved in the mindist sequences for both treatment groups. Treatment group differences are due to sites of non-conserved cysteine residues in four regions of gp160: 1) gp41 cytoplasmic tail (CT), 2) gp41 transmembrane domain (TM), 3) the V1 variable loop (V1), and 4) the signal peptide (SP). At every site with a non-conserved cysteine residue, the frequency is higher in the vaccine group. The non-conserved cysteine residues in V1 are from two vaccine recipient mindist sequences each with one extra pair of cysteines in that region. P-values comparing frequencies by treatment group are non-significant for any given site; the cysteine counts are only significant when aggregated across sites (see S1 Fig).
(PDF)

S3 Table Comparison of intra-host diversity measures across treatment groups. Intra-host mean diversity measures were computed based on pairwise amino acid distances between all sequences from a given subject. Comparisons between vaccine and placebo groups were done using a Wilcoxon rank sum test (Mann-Whitney test) with exact 2-sided p-value. Multiplicity adjusted Q-values were only computed for the analysis including the dually-infected vaccinees.
(PDF)

S4 Table Comparison of pairwise distance measures across treatment. Mean divergence measures were calculated based on pairwise amino acid distances between the vaccine inserts or HIV-1 references and all sequences from a given subject. Comparisons between vaccine and placebo groups were done using a Wilcoxon rank sum test (Mann-Whitney test) with exact 2-sided P-value. Multiplicity adjustment was performed on a subset of tests with Q-value listed as NA if the associated P-value was not included in the adjustment procedure. For proteins included in the vaccine construct, all P-values for each protein/vaccine insert combination were included. For proteins not included in the vaccine construct, only the P-values from the Anc.B based on distances were included. For gp41, which is only partially contained within the vaccine, the P-value from the Anc.B distance was included and the others were excluded.
(PDF)

S5 Table Comparison of tree-based distance measures across treatment. Mean divergence measures were calculated based on tree-based amino acid distances between the vaccine inserts or HIV-1 references and all sequences from a given subject. Comparisons between vaccine and placebo groups were done using a Wilcoxon rank sum test (Mann-Whitney test) with exact 2-sided p-value.
(PDF)

S6 Table Comparison of pairwise and tree-based distance measures across treatment groups using Env-gp120 alignments without variable segments. Distances correspond to the tree-based amino acid distance between the vaccine inserts or HIV-1 references and the breakthrough sequences from a given subject. Comparisons between vaccine and placebo groups were done using Mann-Whitney tests.
(PDF)

S7 Table Site scanning signatures. Site and method for each site scanning result with a Q-value ≤ 0.2. N is the number of AA sites included for each multiple comparison. The Direction (+ for more or–for fewer mismatches in vaccine than placebo sequences) of the effect is with respect to the subtype B vaccine insert for Env, the vaccine insert for Pol and Nef and Ancestral B for Vif and Vpu. For Q-value and FWER adjustment, multiplicity adjustment was done at the gene level except where noted. One site, Pol 238, passes FWER adjustment (in bold) at the 0.05 level. ** Site/property combinations.
(PDF)

S8 Table HIV-1 Env sites under positive selection in the vaccine group only. Eight sites were identified in Env-gp120 using three methods.
(PDF)

S9 Table Percentage of CTL epitopes predicted among breakthrough sequences that were matched to HIV-1 reference sequences. Epitopes predicted in breakthrough sequences were matched against epitopes derived from reference sequences when there were no more than 3 mutations between the 9mers.
(PDF)

S10 Table Comparison of binding affinity measures for predicted CTL epitopes from vaccine and placebo recipients. Epitopes predicted to be strong and weak binders were matched against vaccine inserts or HIV-1 reference sequences and the predicted binding affinity of breakthrough virus-derived epitopes were compared to those of vaccine- and reference-derived epitopes. The distribution of summary values determined for each subject was compared between vaccine and placebo groups using Mann-Whitney tests.
(PDF)

S11 Table Comparison of evolutionary distances for predicted CTL epitopes from vaccine and placebo recipients. Epitopes predicted to be strong and weak binders were matched against vaccine inserts or HIV-1 reference sequences and evolutionary distances were computed between breakthrough virus-derived epitopes and vaccine or reference-derived epitopes. The distribution of summary values determined for each subject was compared between vaccine and placebo groups using Mann-Whitney tests.
(PDF)

S12 Table Comparison of binding affinity measures for predicted CTL epitopes (strong binders only) from vaccine and placebo recipients. Epitopes predicted to be strong binders were matched against vaccine inserts or reference sequences and the predicted binding affinity of breakthrough virus-derived epitopes were compared to those of vaccine- or reference-derived epitopes. The distribution of summary values determined for each subject was compared between vaccine and placebo groups using Mann-Whitney tests.
(PDF)

S13 Table Comparison of evolutionary distances for predicted CTL epitopes (strong binders only) from vaccine and placebo recipients. Epitopes predicted to be strong binders were matched against vaccine inserts or HIV-1 reference sequences and evolutionary distances were computed between breakthrough virus-derived epitopes and vaccine- or reference-derived epitopes. The distribution of summary values determined for each subject was compared between vaccine and placebo groups using Mann-Whitney tests.
(PDF)

S14 Table Comparison between the vaccine and placebo groups for 19 Env-gp120 CTL epitopes. Only epitopes that were identified in at least three vaccine and three placebo recipients were considered for comparison through Mann-Whitney tests. Mean values corresponding to all subjects in each group are reported; for a given subject, the binding affinity or evolutionary distance corresponds to the comparison of the breakthrough virus-derived epitope to the corresponding epitope in the subtype B vaccine.
(PDF)

S15 Table Monoclonal antibody contact set scanning. Results of the mAb contact set scanning analysis grouped by monoclonal antibody class (CD4bs, CD4i, and V3, Quarternary, gp41 MPER, gp41 NHR, gp41 cluster II, and Glycan) and ranked within class by P-value. The effect size is measured in additional mismatch rate in the vaccine group per contact site where a positive (negative) value indicates more (fewer) mismatches at contact site residues in breakthrough sequences for the vaccine group as compared to the placebo group and n is the number of cantact sites. For results with a Q-value ≤ 0.2 the mAb name is in bold.
(PDF)

S16 Table Results of machine learning sieve analysis. Results for the area under the ROC curve (AUC) and classification accuracy (ACC) on held-out data for the four machine learning methods applied over the different regions of the HIV-1 genome. The “region” column indicates the region of the protein for which AA sites were included in the analysis. A null result with no classification capacity is reflected by AUC ≤ 0.5 and ACC ≤ 0.574 (= 27/47).
(PDF)

S17 Table Monoclonal antibody contact site sets. (XLSX)

S1 Fig Mindist sequence cysteine counts. (A) Cysteine counts of gp160 mindist sequences by treatment group. P-values compare counts by treatment using a two-sample t-test. (B) A total of twenty cysteines in gp160 were found in all mindist sequences consisting of 9 pairs in gp120 and 1 pair in gp41. At least one additional cysteine was found in each of the mindist sequences in one or more of the following regions; gp41 cytoplasmic tail (CT), gp41 transmembrane domain (TM), the V1 variable loop (V1), and the signal peptide (SP). Each additional cysteine is represented by a bar of height one color coded by region. When additional cysteine residues were observed they came as a pair in V1 (2 vaccine participant mindist sequences, blue bars), either a pair or singleton in CT and SP (red and purple bars), and as a singleton in TM (2 vaccine participant mindist sequences, green bars).
(PDF)

S2 Fig Amino acid distributions for K-mer region Env 27–37. The amino acid (AA) distribution for linear signature region Env-gp120 AA27-37 by treatment group for all breakthrough sequences. Each row displays the AA distribution for one site relative to the VRC-B vaccine strain AA. Sequences for each participant are represented by a bar of equal height with the reference sequence AA residue, in black, shown above the midline. Within a bar, colors depict the fraction of the participant's sequences with that AA residue (or insertion or deletion, indicated by a dash). The widths of the bars are scaled so that the total width for each treatment group is the same. The Y-axis label indicates the HXB2 position.
(PDF)

S3 Fig Amino acid distributions for 9-mer Env 86–94. The amino acid (AA) distribution for linear signature region Env-gp120 AA86-94 by treatment group for all breakthrough sequences. Each row displays the AA distribution for one site relative to the VRC-B vaccine strain AA. Sequences for each participant are represented by a bar of equal height with the reference sequence AA residue, in black, shown above the midline. Within a bar, colors depict the fraction of the participant's sequences with that AA residue (or insertion or deletion, indicated by a dash). The widths of the bars are scaled so that the total width for each treatment group is the same. The Y-axis label indicates the HXB2 position.
(PDF)

S4 Fig Amino acid distributions for K-mer region Env 192–207. The amino acid (AA) distribution for linear signature region Env-gp120 AA192-207 by treatment group for all breakthrough sequences. Each row displays the AA distribution for one site relative to the VRC-B vaccine strain AA. Sequences for each participant are represented by a bar of equal height with the reference sequence AA residue, in black, shown above the midline. Within a bar, colors depict the fraction of the participant's sequences with that AA residue (or insertion or deletion, indicated by a dash). The widths of the bars are scaled so that the total width for each treatment group is the same. The Y-axis label indicates the HXB2 position.
(PDF)

S5 Fig Amino acid distributions for 9-mer Env 365–373. The amino acid (AA) distribution for linear signature region Env-gp120 AA365-373 by treatment group for all breakthrough sequences. Each row displays the AA distribution for one site relative to the VRC-B vaccine strain AA. Sequences for each participant are represented by a bar of equal height with the reference sequence AA residue, in black, shown above the midline. Within a bar, colors depict the fraction of the participant's sequences with that AA residue (or insertion or deletion, indicated by a dash). The widths of the bars are scaled so that the total width for each treatment group is the same. The Y-axis label indicates the HXB2 position.
(PDF)

S6 Fig Amino acid distributions for K-mer region Env 425–437. The amino acid (AA) distribution for linear signature region Env-gp120 AA425-437 by treatment group for all breakthrough sequences. Each row displays the AA distribution for one site relative to the VRC-B vaccine strain AA. Sequences for each participant are represented by a bar of equal height with the reference sequence AA residue, in black, shown above the midline. Within a bar, colors depict the fraction of the participant's sequences with that AA residue (or insertion or deletion, indicated by a dash). The widths of the bars are scaled so that the total width for each treatment group is the same. The Y-axis label indicates the HXB2 position.
(PDF)

S7 Fig Amino acid distributions for K-mer region Env 464–478. The amino acid (AA) distribution for linear signature region Env-gp120 AA464-478 by treatment group for all breakthrough sequences. Each row displays the AA distribution for one site relative to the VRC-B vaccine strain AA. Sequences for each participant are represented by a bar of equal height with the reference sequence AA residue, in black, shown above the midline. Within a bar, colors depict the fraction of the participant's sequences with that AA residue (or insertion or deletion, indicated by a dash). The widths of the bars are scaled so that the total width for each treatment group is the same. The Y-axis label indicates the HXB2 position.
(PDF)

S8 Fig Percent epitope mismatch analysis. Percent epitope mismatch based on (A) netMHCpan and (B) ADT. For Env, predicted weak binders to all 9-mers from the three vaccine insert sequences (VRC-A, VRC-B, and VRC-C) combined were used to determine the percent epitope mismatch. Similarly, percent epitope mismatch was computed using all of the non-insert genes of HIV-1 combined. Mismatch percent for vaccine recipients in red and placebo recipients in blue. P-values are from a choplump Wilcoxon test.
(PDF)

S9 Fig **Antibody-dependent cellular phagocytosis (ADCP) activity of antibodies induced by the DNA/rAd5 vaccine regimen at (A) baseline and (B) 4 weeks post-4th vaccination.** Neutravidin fluorescent beads were coated with a biotinylated HIV-1 antigen (ConSgp140), then incubated with monoclonal antibodies (positive control CH31 and negative control CH65) or IgG purified from participant serum samples. THP-1 cells (pre-treated with anti-human CD4 to reduce CD4-Env mediated virus internalization) were incubated with the antibody/bead mixture, then paraformaldehyde-fixed before analysis by flow cytometry. A phagocytic score was determined based on the ratio of experimental sample to PBS control. For further details, see S1 Supplementary Methods. Data from responders are shown in red and non-responders in blue, with box plots based on data from responders superimposed on the distribution. The mid-line of the box denotes the median and the ends of the box denote the 25th and 75th percentiles. The whiskers that extend from the top and bottom of the box extend to the most extreme data points that are no more than 1.5 times the interquartile range (i.e., height of the box) or if no value meets this criterion, to the data extremes.
(PDF)

S1 Supplementary Methods Additional information on HIV-1 sequences, sequence analysis, CTL epitope predictions, site scanning methods, machine learning highly multivariable sieve analysis, antibody-dependent cellular phagocytosis (ADCP), and references. (DOCX)
