# Supplementary material for: Sieve analysis of breakthrough HIV-1 sequences in HVTN 505 identifies vaccine pressure targeting the CD4 binding site of Env-gp120
Source: PLoS One. 2017 Nov 17;12(11):e0185959. doi: 10.1371/journal.pone.0185959 (PMC5693417; doi:10.1371/journal.pone.0185959)
Supplement: S1 Table — Numbers of participants with primary endpoint HIV-1 infection included in the sieve analysis, mean time between HIV-1 infection and sampling for HIV-1 sequencing, and antibody testing. (PDF) [file pone.0185959.s001.pdf]

Table S1. Mean time since infection and antibody testing.
Numbers of participants with primary endpoint HIV-1 infection included in the sieve analysis, mean time between HIV-1 infection and sampling for HIV-1 sequencing, and antibody testing.

Treatment Group	Number of infected ppts (per protocol)	Mean time since infection in days[#]	WB[§]		ELISA		Time between study entry and diagnosis		
			Pos.	Neg. or Ind.	Pos.	Neg. or Ind.	12 months	18 months	24 months
Placebo	20 (18)	43	16	3	18	2	8	9	10
Vaccine	27 (24)	49.5	23	0	21	6	12	5	3

[#] Time since infection is defined as half the time between the last RNA negative and first RNA positive samples plus the time from first positive sample to the draw date.

[§] WB = Western blot. Five samples do not have a WB test since the draw date is prior to the diagnosis date.
