# Supplementary material for: Sieve analysis of breakthrough HIV-1 sequences in HVTN 505 identifies vaccine pressure targeting the CD4 binding site of Env-gp120
Source: PLoS One. 2017 Nov 17;12(11):e0185959. doi: 10.1371/journal.pone.0185959 (PMC5693417; doi:10.1371/journal.pone.0185959)
Supplement: S2 Table — Subtype B viruses typically possess 18 cysteine residues forming 9 disulfide bridges in gp120 and 2 cysteine residues in gp41 forming an additional disulfide bridge for a total of 20 conserved cysteine residues. These residues are also conserved in the mindist sequences for both treatment groups. Treatment group differences are due to sites of non-conserved cysteine residues in four regions of gp160: 1) gp41 cytoplasmic tail (CT), 2) gp41 transmembrane domain (TM), 3) the V1 variable loop (V1), and 4) the signal peptide (SP). At every site with a non-conserved cysteine residue, the frequency is higher in the vaccine group. The non-conserved cysteine residues in V1 are from two vaccine recipient mindist sequences each with one extra pair of cysteines in that region. P-values comparing frequencies by treatment group are non-significant for any given site; the cysteine counts are only significant when aggregated across sites (see S1 Fig). (PDF) [file pone.0185959.s002.pdf]

Table S2. Non-conserved cysteine residue frequencies in *mindist* sequences.

Subtype B viruses typically possess 18 cysteine residues forming 9 disulfide bridges in gp120 and 2 cysteine residues in gp41 forming an additional disulfide bridge for a total of 20 conserved cysteine residues. These residues are also conserved in the mindist sequences for both treatment groups. Treatment group differences are due to sites of non-conserved cysteine residues in four regions of gp160: 1) gp41 cytoplasmic tail (CT), 2) gp41 transmembrane domain (TM), 3) the V1 variable loop (V1), and 4) the signal peptide (SP). At every site with a non-conserved cysteine residue, the frequency is higher in the vaccine group. The non-conserved cysteine residues in V1 are from two vaccine recipient mindist sequences each with one extra pair of cysteines in that region. P-values comparing frequencies by treatment group are non-significant ($p > 0.05$) for any given site; the cysteine counts are only significant when aggregated across sites (see Figure S4).

Counts and Frequency						
HXB2 position	Region	Placebo		Vaccine		P-value
10	SP	3/20	15%	10/27	37.0%	0.11
13	SP	0/20	0%	1/27	3.7%	1
28	SP	19/20	95%	26/27	96.3%	1
29d	SP	0/20	0%	1/27	3.7%	1
133b	V1	0/20	0%	1/27	3.7%	1
133m	V1	0/20	0%	2/27	7.4%	0.5
143	V1	0/20	0%	1/27	3.7%	1
699	TM	0/20	0%	2/27	7.4%	0.5
764	CT	9/20	45%	18/27	66.7%	0.23
768	CT	0/20	0%	1/27	3.7%	1
796	CT	0/20	0%	3/27	11.1%	0.25
837	CT	3/20	15%	5/27	18.5%	1
