# Supplementary material for: Sieve analysis of breakthrough HIV-1 sequences in HVTN 505 identifies vaccine pressure targeting the CD4 binding site of Env-gp120
Source: PLoS One. 2017 Nov 17;12(11):e0185959. doi: 10.1371/journal.pone.0185959 (PMC5693417; doi:10.1371/journal.pone.0185959)
Supplement: S3 Table — Intra-host mean diversity measures were computed based on pairwise amino acid distances between all sequences from a given subject. Comparisons between vaccine and placebo groups were done using a Wilcoxon rank sum test (Mann-Whitney test) with exact 2-sided p-value. Multiplicity adjusted Q-values were only computed for the analysis including the dually-infected vaccinees. (PDF) [file pone.0185959.s003.pdf]

Table S3. Comparison of intra-host diversity measures across treatment groups.

Intra-host mean diversity measures were computed based on pairwise amino acid distances between all sequences from a given subject. Comparisons between vaccine and placebo groups were done using a Wilcoxon rank sum test (Mann-Whitney test) with exact 2-sided p-value. Multiplicity adjustmented Q-values were only computed for the analysis including the daully-infected vaccinees.

	**gp120**		**gp41**		**Gag**		**Pol**		**Nef**	
	**Vaccine**	**Placebo**	**Vaccine**	**Placebo**	**Vaccine**	**Placebo**	**Vaccine**	**Placebo**	**Vaccine**	**Placebo**
n	27	20	27	20	26	20	26	20	27	20
Median	0.003	0.005	0.002	0.003	0.001	0.002	0.00079	0.00199	0.002	0.005
Mean	0.019	0.010	0.010	0.006	0.004	0.004	0.00398	0.00303	0.012	0.007
p-value	**0.035**		0.241		**0.036**		**0.006**		0.331	
q-value	**0.090**		0.402		**0.090**		**0.060**		0.473	

	**Rev**		**Tat**		**Vif**		**Vpr**		**Vpu**	
	**Vaccine**	**Placebo**	**Vaccine**	**Placebo**	**Vaccine**	**Placebo**	**Vaccine**	**Placebo**	**Vaccine**	**Placebo**
n	27	20	27	20	26	20	26	20	27	20
Median	0.003	0.004	0.002	0.002	0.001	0.004	0.001	0.002	0.002	0.005
Mean	0.014	0.007	0.014	0.006	0.007	0.007	0.008	0.005	0.016	0.007
p-value	0.600		0.882		**0.036**		0.541		0.194	
q-value	0.667		0.882		**0.090**		0.667		0.388	

Excluding two dually-infected vaccinees

	**gp120**		**gp41**		**Gag**		**Pol**		**Nef**	
	**Vaccine**	**Placebo**	**Vaccine**	**Placebo**	**Vaccine**	**Placebo**	**Vaccine**	**Placebo**	**Vaccine**	**Placebo**
n	25	20	25	20	24	20	24	20	25	20
Median	0.002	0.005	0.002	0.003	0.001	0.002	0.00076	0.00199	0.001	0.005
Mean	0.005	0.010	0.003	0.006	0.001	0.004	0.00116	0.00303	0.004	0.007
p-value	**0.006**		0.088		**0.006**		**0.001**		0.135	

	**Rev**		**Tat**		**Vif**		**Vpr**		**Vpu**	
	**Vaccine**	**Placebo**	**Vaccine**	**Placebo**	**Vaccine**	**Placebo**	**Vaccine**	**Placebo**	**Vaccine**	**Placebo**
n	25	20	25	20	24	20	24	20	25	20
Median	0.002	0.004	0.002	0.002	0.001	0.004	0.000	0.002	0.002	0.005
Mean	0.005	0.007	0.003	0.006	0.002	0.007	0.002	0.005	0.004	0.007
p-value	0.308		0.765		**0.006**		0.246		0.064	
