# Supplementary material for: Sieve analysis of breakthrough HIV-1 sequences in HVTN 505 identifies vaccine pressure targeting the CD4 binding site of Env-gp120
Source: PLoS One. 2017 Nov 17;12(11):e0185959. doi: 10.1371/journal.pone.0185959 (PMC5693417; doi:10.1371/journal.pone.0185959)
Supplement: S4 Table — Mean divergence measures were calculated based on pairwise amino acid distances between the vaccine inserts or HIV-1 references and all sequences from a given subject. Comparisons between vaccine and placebo groups were done using a Wilcoxon rank sum test (Mann-Whitney test) with exact 2-sided P-value. Multiplicity adjustment was performed on a subset of tests with Q-value listed as NA if the associated P-value was not included in the adjustment procedure. For proteins included in the vaccine construct, all P-values for each protein/vaccine insert combination were included. For proteins not included in the vaccine construct, only the P-values from the Anc.B based on distances were included. For gp41, which is only partially contained within the vaccine, the P-value from the Anc.B distance was included and the others were excluded. (PDF) [file pone.0185959.s004.pdf]

Table S4. Comparison of pairwise distance measures across treatment.

Mean divergence measures were calculated based on pairwise amino acid distances between the vaccine inserts or HIV-1 references and all sequences from a given subject. Comparisons between vaccine and placebo groups were done using a Wilcoxon rank sum test (Mann-Whitney test) with exact 2-sided P-value. Multiplicity adjustment was performed on a subset of tests with Q-value listed as NA if the associated P-value was not included in the adjustment procedure. For proteins included in the vaccine construct, all P-values for each protein/vaccine insert combination were included. For proteins not included in the vaccine construct, only the P-values from the Anc.B based on distances were included. For gp41, which is only partially contained within the vaccine, the P-value from the Anc.B distance was included and the others were excluded.

AA pw gp120

	VRC-A		VRC-B		VRC-C		Cons.B		Anc.B		MRCA		HXB2	
	**Vaccine**	**Placebo**	**Vaccine**	**Placebo**	**Vaccine**	**Placebo**	**Vaccine**	**Placebo**	**Vaccine**	**Placebo**	**Vaccine**	**Placebo**	**Vaccine**	**Placebo**
**n**	27	20	27	20	27	20	27	20	27	20	27	20	27	20
**Median**	0.37	0.356	0.299	0.288	0.418	0.405	0.232	0.209	0.235	0.214	0.241	0.236	0.29	0.28
**Mean**	0.373	0.364	0.307	0.285	0.418	0.405	0.229	0.205	0.231	0.213	0.247	0.238	0.297	0.276
**P value**	0.1037		**0.0098**		0.0825		**0.0049**		**0.0431**		0.287		**0.0135**	
**Q value**	0.311		**0.118**		0.311		NA		NA		NA		NA	

AA pw gp41

	VRC-A		VRC-B		VRC-C		Cons.B		Anc.B		MRCA		HXB2	
	**Vaccine**	**Placebo**	**Vaccine**	**Placebo**	**Vaccine**	**Placebo**	**Vaccine**	**Placebo**	**Vaccine**	**Placebo**	**Vaccine**	**Placebo**	**Vaccine**	**Placebo**
**n**	27	20	27	20	27	20	27	20	27	20	27	20	27	20
**Median**	1.73	1.7	0.549	0.547	0.622	0.603	0.135	0.14	0.14	0.138	0.161	0.155	0.172	0.177
**Mean**	1.72	1.71	0.551	0.55	0.619	0.601	0.143	0.139	0.143	0.139	0.161	0.155	0.181	0.183
**P value**	0.7185		0.783		0.0787		0.8323		0.6714		0.5957		0.5985	
**Q value**	NA		NA		NA		NA		0.743		NA		NA	

AA pw Gag

	VRC-B		Cons.B		Anc.B		HXB2	
	**Vaccine**	**Placebo**	**Vaccine**	**Placebo**	**Vaccine**	**Placebo**	**Vaccine**	**Placebo**
**n**	26	20	26	20	26	20	26	20
**Median**	0.0804	0.0817	0.071	0.0735	0.0757	0.0797	0.0876	0.0866
**Mean**	0.0814	0.0842	0.0723	0.0754	0.0747	0.0816	0.0878	0.0894
**P value**	0.6812		0.6812		0.1352		0.8137	
**Q value**	0.743		NA		NA		NA	

AA pw Pol

	VRC-B		Cons.B		Anc.B		HXB2	
	Vaccine	Placebo	Vaccine	Placebo	Vaccine	Placebo	Vaccine	Placebo
**n**	26	20	26	20	26	20	26	20
**Median**	0.0591	0.0616	0.0495	0.0516	0.0521	0.0549	0.0607	0.06
**Mean**	0.0606	0.063	0.0492	0.0505	0.0519	0.0539	0.0604	0.0601
**P value**	0.4924		0.6494		0.4202		0.8964	
**Q value**	0.743		NA		NA		NA	

AA pw Nef

	VRC-B		Cons.B		Anc.B		HXB2	
	Vaccine	Placebo	Vaccine	Placebo	Vaccine	Placebo	Vaccine	Placebo
**n**	27	20	27	20	27	20	27	20
**Median**	0.22	0.193	0.166	0.152	0.174	0.16	0.217	0.205
**Mean**	0.214	0.195	0.161	0.154	0.177	0.159	0.222	0.204
**P value**	0.0864		0.2774		0.1523		0.1345	
**Q value**	0.311		NA		NA		NA	

AA pw Rev

	Cons.B		Anc.B		HXB2	
	Vaccine	Placebo	Vaccine	Placebo	Vaccine	Placebo
**n**	27	20	27	20	27	20
**Median**	0.154	0.166	0.155	0.169	0.181	0.198
**Mean**	0.16	0.159	0.163	0.164	0.183	0.191
**P value**	0.9362		0.6134		0.4092	
**Q value**	NA		0.743		NA	

AA pw Tat

	Cons.B		Anc.B		HXB2	
	Vaccine	Placebo	Vaccine	Placebo	Vaccine	Placebo
**n**	27	20	27	20	27	20
**Median**	0.173	0.167	0.187	0.184	0.178	0.201
**Mean**	0.172	0.17	0.187	0.189	0.191	0.201
**P value**	0.8857		0.9531		0.5549	
	NA		0.953		NA	

AA pw Vif

	Cons.B		Anc.B		HXB2	
	Vaccine	Placebo	Vaccine	Placebo	Vaccine	Placebo
**n**	26	20	26	20	26	20
**Median**	0.107	0.123	0.116	0.123	0.13	0.14
**Mean**	0.111	0.12	0.113	0.119	0.129	0.138
**P value**	0.2373		0.433		0.2943	
**Q vlaue**	NA		0.743		NA	

AA pw Vpr

	Cons.B		Anc.B		HXB2	
	Vaccine	Placebo	Vaccine	Placebo	Vaccine	Placebo
**n**	26	20	26	20	26	20
**Median**	0.0926	0.091	0.0953	0.0909	0.118	0.116
**Mean**	0.0964	0.0923	0.0964	0.086	0.12	0.118
**P value**	0.777		0.303		0.8619	
	NA		0.727		NA	

AA pw Vpu

	Cons.B		Anc.B		HXB2	
	Vaccine	Placebo	Vaccine	Placebo	Vaccine	Placebo
**n**	27	20	27	20	27	20
**Median**	0.166	0.156	0.162	0.166	0.29	0.287
**Mean**	0.178	0.16	0.179	0.168	0.286	0.281
**P value**	0.3068		0.5241		0.8489	
**Q value**	NA		0.743		NA	
