# Supplementary material for: Sieve analysis of breakthrough HIV-1 sequences in HVTN 505 identifies vaccine pressure targeting the CD4 binding site of Env-gp120
Source: PLoS One. 2017 Nov 17;12(11):e0185959. doi: 10.1371/journal.pone.0185959 (PMC5693417; doi:10.1371/journal.pone.0185959)
Supplement: S5 Table — Mean divergence measures were calculated based on tree-based amino acid distances between the vaccine inserts or HIV-1 references and all sequences from a given subject. Comparisons between vaccine and placebo groups were done using a Wilcoxon rank sum test (Mann-Whitney test) with exact 2-sided p-value. (PDF) [file pone.0185959.s005.pdf]

Table S5. Comparison of tree-based distance measures across treatment.

Mean divergence measures were calculated based on tree-based amino acid distances between the vaccine inserts or HIV-1 references and all sequences from a given subject. Comparisons between vaccine and placebo groups were done using a Wilcoxon rank sum test (Mann-Whitney test) with exact 2-sided p-value.

AA tb gp120

	VRC-A		VRC-B		VRC-C		Cons.B		Anc.B		MRCA		HXB2	
	Vaccine	Placebo	Vaccine	Placebo	Vaccine	Placebo	Vaccine	Placebo	Vaccine	Placebo	Vaccine	Placebo	Vaccine	Placebo
**n**	27	20	27	20	27	20	27	20	27	20	27	20	27	20
**Median**	0.914	0.89	0.682	0.657	1.12	1.1	0.557	0.532	0.576	0.551	0.478	0.454	0.67	0.645
**Mean**	0.92	0.906	0.682	0.663	1.12	1.11	0.556	0.538	0.576	0.558	0.486	0.47	0.67	0.652
**P value**	0.395		0.3383		0.4701		0.3383		0.3383		0.3276		0.3383	

AA tb gp41

	VRC-A		VRC-B		VRC-C		Cons.B		Anc.B		MRCA		HXB2	
	Vaccine	Placebo	Vaccine	Placebo	Vaccine	Placebo	Vaccine	Placebo	Vaccine	Placebo	Vaccine	Placebo	Vaccine	Placebo
**n**	27	20	27	20	27	20	27	20	27	20	27	20	27	20
**Median**	4.6	4.6	1.5	1.5	1.87	1.87	0.274	0.287	0.286	0.299	0.337	0.337	0.38	0.393
**Mean**	4.61	4.6	1.51	1.5	1.88	1.87	0.287	0.29	0.299	0.302	0.34	0.34	0.388	0.396
**P value**	0.6407		0.6407		0.6407		0.7216		0.7376		0.7058		0.6436	

AA tb Gag

	VRC-B		Cons.B		Anc.B		HXB2	
	Vaccine	Placebo	Vaccine	Placebo	Vaccine	Placebo	Vaccine	Placebo
**n**	26	20	26	20	26	20	26	20
**Median**	0.114	0.121	0.0996	0.107	0.115	0.122	0.123	0.13
**Mean**	0.114	0.123	0.101	0.109	0.116	0.124	0.124	0.132
**P value**	0.2647		0.3368		0.2647		0.2647	

AA tb Pol

	VRC-B		Cons.B		Anc.B		HXB2	
	Vaccine	Placebo	Vaccine	Placebo	Vaccine	Placebo	Vaccine	Placebo
**n**	26	20	26	20	26	20	26	20
**Median**	0.0903	0.0924	0.0728	0.0749	0.0804	0.0825	0.0996	0.102
**Mean**	0.0899	0.0926	0.0723	0.0751	0.0799	0.0827	0.0973	0.102
**P value**	0.446		0.446		0.446		0.3151	

AA tb Nef

	VRC-B		Cons.B		Anc.B		HXB2	
	Vaccine	Placebo	Vaccine	Placebo	Vaccine	Placebo	Vaccine	Placebo
**n**	27	20	27	20	27	20	27	20
**Median**	0.407	0.425	0.305	0.314	0.324	0.333	0.426	0.444
**Mean**	0.409	0.418	0.298	0.302	0.317	0.321	0.427	0.437
**P value**	0.7862		0.987		0.987		0.7862	

AA tb Rev

	Cons.B		Anc.B		HXB2	
	Vaccine	Placebo	Vaccine	Placebo	Vaccine	Placebo
**n**	27	20	27	20	27	20
**Median**	0.234	0.249	0.256	0.27	0.359	0.385
**Mean**	0.236	0.24	0.257	0.262	0.359	0.36
**P value**	0.6134		0.6134		0.6436	

AA tb Tat

	Cons.B		Anc.B		HXB2	
	Vaccine	Placebo	Vaccine	Placebo	Vaccine	Placebo
**n**	27	20	27	20	27	20
**Median**	0.277	0.272	0.318	0.313	0.307	0.303
**Mean**	0.285	0.28	0.326	0.321	0.329	0.318
**P value**	0.9835		0.9835		0.783	

AA tb Vif

	Cons.B		Anc.B		HXB2	
	Vaccine	Placebo	Vaccine	Placebo	Vaccine	Placebo
**n**	26	20	26	20	26	20
**Median**	0.168	0.169	0.17	0.174	0.217	0.217
**Mean**	0.168	0.175	0.168	0.176	0.215	0.223
**P value**	0.8307		0.6181		0.8137	

AA tb Vpr

	Cons.B		Anc.B		HXB2	
	Vaccine	Placebo	Vaccine	Placebo	Vaccine	Placebo
**n**	26	20	26	20	26	20
**Median**	0.185	0.154	0.267	0.239	0.228	0.198
**Mean**	0.186	0.153	0.255	0.221	0.229	0.196
**P value**	0.0633		0.1404		0.0633	

AA tb Vpu

	Cons.B		Anc.B		HXB2	
	Vaccine	Placebo	Vaccine	Placebo	Vaccine	Placebo
**n**	27	20	27	20	27	20
**Median**	0.335	0.354	0.378	0.397	0.633	0.603
**Mean**	0.381	0.336	0.424	0.377	0.63	0.596
**P value**	0.4967		0.4701		0.3171	
