# Supplementary material for: Sieve analysis of breakthrough HIV-1 sequences in HVTN 505 identifies vaccine pressure targeting the CD4 binding site of Env-gp120
Source: PLoS One. 2017 Nov 17;12(11):e0185959. doi: 10.1371/journal.pone.0185959 (PMC5693417; doi:10.1371/journal.pone.0185959)
Supplement: S6 Table — Distances correspond to the tree-based amino acid distance between the vaccine inserts or HIV-1 references and the breakthrough sequences from a given subject. Comparisons between vaccine and placebo groups were done using Mann-Whitney tests. (PDF) [file pone.0185959.s006.pdf]

Table S6. Comparison pairwise and tree-based distance measures across treatment groups using Env-gp120 alignments without variable segments.

Distances correspond to the tree-based amino acid distance between the vaccine inserts or HIV-1 references and the breakthrough sequences from a given subject. Comparisons between vaccine and placebo groups were done using Mann-Whitney tests.

AA pw gp120 - Variable loops deleted

	**VRC-A**		**VRC-B**		**VRC-C**		**Cons.B.04**		**Anc.B**		**MRCA**		**HXB2**	
	**Vaccine**	**Placebo**	**Vaccine**	**Placebo**	**Vaccine**	**Placebo**	**Vaccine**	**Placebo**	**Vaccine**	**Placebo**	**Vaccine**	**Placebo**	**Vaccine**	**Placebo**
**n**	27	20	27	20	27	20	27	20	27	20	27	20	27	20
**Median**	0.300	0.289	0.222	0.210	0.341	0.328	0.159	0.138	0.161	0.144	0.264	0.249	0.210	0.192
**Mean**	0.303	0.292	0.229	0.208	0.346	0.331	0.158	0.139	0.160	0.148	0.267	0.249	0.214	0.196
**P value**	0.091		**0.004**		**0.015**		**0.014**		0.075		**0.007**		**0.023**	

AA tb gp120 - Variable loops deleted

	**VRC-A**		**VRC-B**		**VRC-C**		**Cons.B.04**		**Anc.B**		**MRCA**		**HXB2**	
	**Vaccine**	**Placebo**	**Vaccine**	**Placebo**	**Vaccine**	**Placebo**	**Vaccine**	**Placebo**	**Vaccine**	**Placebo**	**Vaccine**	**Placebo**	**Vaccine**	**Placebo**
**n**	27	20	27	20	27	20	27	20	27	20	27	20	27	20
**Median**	0.607	0.571	0.402	0.359	0.738	0.701	0.272	0.229	0.293	0.249	0.539	0.502	0.389	0.346
**Mean**	0.615	0.573	0.405	0.370	0.746	0.703	0.275	0.240	0.295	0.261	0.546	0.504	0.392	0.357
**P value**	**0.005**		**0.025**		**0.005**		**0.025**		**0.025**		**0.005**		**0.025**	
