# Supplementary material for: Sieve analysis of breakthrough HIV-1 sequences in HVTN 505 identifies vaccine pressure targeting the CD4 binding site of Env-gp120
Source: PLoS One. 2017 Nov 17;12(11):e0185959. doi: 10.1371/journal.pone.0185959 (PMC5693417; doi:10.1371/journal.pone.0185959)
Supplement: S7 Table — Site and method for each site scanning result with a Q-value ≤ 0.2. N is the number of AA sites included for each multiple comparison. The Direction (+ for more or–for fewer mismatches in vaccine than placebo sequences) of the effect is with respect to the subtype B vaccine insert for Env, the vaccine insert for Pol and Nef and Ancestral B for Vif and Vpu. For Q-value and FWER adjustment, multiplicity adjustment was done at the gene level except where noted. One site, Pol 238, passes FWER adjustment (in bold) at the 0.05 level. ** Site/property combinations. (PDF) [file pone.0185959.s007.pdf]

Table S7. Site scanning signatures.

Site and method for each site scanning result with a Q-value ≤ 0.2. N is the number of AA sites included for each multiple comparison. The Direction (+ for more or – for fewer mismatches in vaccine than placebo sequences) of the effect is with respect to the subtype B vaccine insert for Env, the vaccine insert for Pol and Nef and Ancestral B for Vif and Vpu. For Q-value and FWER adjustment, multiplicity adjustment was done at the gene level except where noted. One site, Pol 238, passes FWER adjustment (in bold) at the 0.05 level. ** Site/property combinations.

Site	Method	N	Direction	P-value	Q-value	Adjusted P-value*
Env 133	PCP (charged)	316**	+	0.00015	0.19	0.19
Env 429[$]	MBS	98	+	0.0014	0.15	0.15
Pol 238	GWJ	180	−	0.0006	0.11	0.11
Pol 238	EGWJ	245	−	0.0002	0.05	**0.05**
Pol 238	QEMD	180	−	0.0006	0.11	0.11
Nef 83	EGWJ	94	+	0.0014	0.13	0.13
Vif 122	MBS	65	+	0.001	0.07	0.07
Vpu 69[#]	QEMD	65	−	0.0018	0.08	0.08
Vpu 69a[#]	QEMD	65	−	0.013	0.15	0.55
Vpu 69b[#]	QEMD	65	−	0.013	0.15	0.55
Vpu 69c[#]	QEMD	65	−	0.013	0.15	0.55

[#]Vpu69-69c signatures are due to insertions in 6 placebo recipient breakthrough *mindist* sequences of between 1 and 4 amino acids.

[$]Multiplicity adjustment is by gene except for Env 429, which is over the set of mAb contact sites.
