# Supplementary material for: Sieve analysis of breakthrough HIV-1 sequences in HVTN 505 identifies vaccine pressure targeting the CD4 binding site of Env-gp120
Source: PLoS One. 2017 Nov 17;12(11):e0185959. doi: 10.1371/journal.pone.0185959 (PMC5693417; doi:10.1371/journal.pone.0185959)
Supplement: S8 Table — Eight sites were identified in Env-gp120 using three methods. (PDF) [file pone.0185959.s008.pdf]

Table S1. Mean time since infection and antibody testing.

Numbers of participants with primary endpoint HIV-1 infection included in the sieve analysis, mean time between HIV-1 infection and sampling for HIV-1 sequencing, and antibody testing.

Treatment Group	Number of infected ppts (per protocol)	Mean time since infection in days[#]	WB[§]		ELISA		Time between study entry and diagnosis		
			Pos.	Neg. or Ind.	Pos.	Neg. or Ind.	12 months	18 months	24 months
Placebo	20 (18)	43	16	3	18	2	8	9	10
Vaccine	27 (24)	49.5	23	0	21	6	12	5	3

[#] Time since infection is defined as half the time between the last RNA negative and first RNA positive samples plus the time from first positive sample to the draw date.

[§] WB = Western blot. Five samples do not have a WB test since the draw date is prior to the diagnosis date.

Table S2. Non-conserved cysteine residue frequencies in *mindist* sequences.

Subtype B viruses typically possess 18 cysteine residues forming 9 disulfide bridges in gp120 and 2 cysteine residues in gp41 forming an additional disulfide bridge for a total of 20 conserved cysteine residues. These residues are also conserved in the mindist sequences for both treatment groups. Treatment group differences are due to sites of non-conserved cysteine residues in four regions of gp160: 1) gp41 cytoplasmic tail (CT), 2) gp41 transmembrane domain (TM), 3) the V1 variable loop (V1), and 4) the signal peptide (SP). At every site with a non-conserved cysteine residue, the frequency is higher in the vaccine group. The non-conserved cysteine residues in V1 are from two vaccine recipient mindist sequences each with one extra pair of cysteines in that region. P-values comparing frequencies by treatment group are non-significant ($p > 0.05$) for any given site; the cysteine counts are only significant when aggregated across sites (see Figure S4).

Counts and Frequency						
HXB2 position	Region	Placebo		Vaccine		P-value
10	SP	3/20	15%	10/27	37.0%	0.11
13	SP	0/20	0%	1/27	3.7%	1
28	SP	19/20	95%	26/27	96.3%	1
29d	SP	0/20	0%	1/27	3.7%	1
133b	V1	0/20	0%	1/27	3.7%	1
133m	V1	0/20	0%	2/27	7.4%	0.5
143	V1	0/20	0%	1/27	3.7%	1
699	TM	0/20	0%	2/27	7.4%	0.5
764	CT	9/20	45%	18/27	66.7%	0.23
768	CT	0/20	0%	1/27	3.7%	1
796	CT	0/20	0%	3/27	11.1%	0.25
837	CT	3/20	15%	5/27	18.5%	1

Table S3. Comparison of intra-host diversity measures across treatment groups.

Intra-host mean diversity measures were computed based on pairwise amino acid distances between all sequences from a given subject. Comparisons between vaccine and placebo groups were done using a Wilcoxon rank sum test (Mann-Whitney test) with exact 2-sided p-value. Multiplicity adjustmented Q-values were only computed for the analysis including the daully-infected vaccinees.

	**gp120**		**gp41**		**Gag**		**Pol**		**Nef**	
	**Vaccine**	**Placebo**	**Vaccine**	**Placebo**	**Vaccine**	**Placebo**	**Vaccine**	**Placebo**	**Vaccine**	**Placebo**
n	27	20	27	20	26	20	26	20	27	20
Median	0.003	0.005	0.002	0.003	0.001	0.002	0.00079	0.00199	0.002	0.005
Mean	0.019	0.010	0.010	0.006	0.004	0.004	0.00398	0.00303	0.012	0.007
p-value	**0.035**		0.241		**0.036**		**0.006**		0.331	
q-value	**0.090**		0.402		**0.090**		**0.060**		0.473	

	**Rev**		**Tat**		**Vif**		**Vpr**		**Vpu**	
	**Vaccine**	**Placebo**	**Vaccine**	**Placebo**	**Vaccine**	**Placebo**	**Vaccine**	**Placebo**	**Vaccine**	**Placebo**
n	27	20	27	20	26	20	26	20	27	20
Median	0.003	0.004	0.002	0.002	0.001	0.004	0.001	0.002	0.002	0.005
Mean	0.014	0.007	0.014	0.006	0.007	0.007	0.008	0.005	0.016	0.007
p-value	0.600		0.882		**0.036**		0.541		0.194	
q-value	0.667		0.882		**0.090**		0.667		0.388	

Excluding two dually-infected vaccinees

	**gp120**		**gp41**		**Gag**		**Pol**		**Nef**	
	**Vaccine**	**Placebo**	**Vaccine**	**Placebo**	**Vaccine**	**Placebo**	**Vaccine**	**Placebo**	**Vaccine**	**Placebo**
n	25	20	25	20	24	20	24	20	25	20
Median	0.002	0.005	0.002	0.003	0.001	0.002	0.00076	0.00199	0.001	0.005
Mean	0.005	0.010	0.003	0.006	0.001	0.004	0.00116	0.00303	0.004	0.007
p-value	**0.006**		0.088		**0.006**		**0.001**		0.135	

	**Rev**		**Tat**		**Vif**		**Vpr**		**Vpu**	
	**Vaccine**	**Placebo**	**Vaccine**	**Placebo**	**Vaccine**	**Placebo**	**Vaccine**	**Placebo**	**Vaccine**	**Placebo**
n	25	20	25	20	24	20	24	20	25	20
Median	0.002	0.004	0.002	0.002	0.001	0.004	0.000	0.002	0.002	0.005
Mean	0.005	0.007	0.003	0.006	0.002	0.007	0.002	0.005	0.004	0.007
p-value	0.308		0.765		**0.006**		0.246		0.064	

Table S4. Comparison of pairwise distance measures across treatment.

Mean divergence measures were calculated based on pairwise amino acid distances between the vaccine inserts or HIV-1 references and all sequences from a given subject. Comparisons between vaccine and placebo groups were done using a Wilcoxon rank sum test (Mann-Whitney test) with exact 2-sided P-value. Multiplicity adjustment was performed on a subset of tests with Q-value listed as NA if the associated P-value was not included in the adjustment procedure. For proteins included in the vaccine construct, all P-values for each protein/vaccine insert combination were included. For proteins not included in the vaccine construct, only the P-values from the Anc.B based on distances were included. For gp41, which is only partially contained within the vaccine, the P-value from the Anc.B distance was included and the others were excluded.

AA pw gp120

	VRC-A		VRC-B		VRC-C		Cons.B		Anc.B		MRCA		HXB2	
	Vaccine	Placebo	Vaccine	Placebo	Vaccine	Placebo	Vaccine	Placebo	Vaccine	Placebo	Vaccine	Placebo	Vaccine	Placebo
**n**	27	20	27	20	27	20	27	20	27	20	27	20	27	20
**Median**	0.37	0.356	0.299	0.288	0.418	0.405	0.232	0.209	0.235	0.214	0.241	0.236	0.29	0.28
**Mean**	0.373	0.364	0.307	0.285	0.418	0.405	0.229	0.205	0.231	0.213	0.247	0.238	0.297	0.276
**P value**	0.1037		**0.0098**		0.0825		**0.0049**		**0.0431**		0.287		**0.0135**	
**Q value**	0.311		**0.118**		0.311		NA		NA		NA		NA	

AA pw gp41

	VRC-A		VRC-B		VRC-C		Cons.B		Anc.B		MRCA		HXB2	
	Vaccine	Placebo	Vaccine	Placebo	Vaccine	Placebo	Vaccine	Placebo	Vaccine	Placebo	Vaccine	Placebo	Vaccine	Placebo
**n**	27	20	27	20	27	20	27	20	27	20	27	20	27	20
**Median**	1.73	1.7	0.549	0.547	0.622	0.603	0.135	0.14	0.14	0.138	0.161	0.155	0.172	0.177
**Mean**	1.72	1.71	0.551	0.55	0.619	0.601	0.143	0.139	0.143	0.139	0.161	0.155	0.181	0.183
**P value**	0.7185		0.783		0.0787		0.8323		0.6714		0.5957		0.5985	
**Q value**	NA		NA		NA		NA		0.743		NA		NA	

AA pw Gag

	VRC-B		Cons.B		Anc.B		HXB2	
	Vaccine	Placebo	Vaccine	Placebo	Vaccine	Placebo	Vaccine	Placebo
**n**	26	20	26	20	26	20	26	20
**Median**	0.0804	0.0817	0.071	0.0735	0.0757	0.0797	0.0876	0.0866
**Mean**	0.0814	0.0842	0.0723	0.0754	0.0747	0.0816	0.0878	0.0894
**P value**	0.6812		0.6812		0.1352		0.8137	
**Q value**	0.743		NA		NA		NA	

AA pw Pol

	VRC-B		Cons.B		Anc.B		HXB2	
	Vaccine	Placebo	Vaccine	Placebo	Vaccine	Placebo	Vaccine	Placebo
**n**	26	20	26	20	26	20	26	20
**Median**	0.0591	0.0616	0.0495	0.0516	0.0521	0.0549	0.0607	0.06
**Mean**	0.0606	0.063	0.0492	0.0505	0.0519	0.0539	0.0604	0.0601
**P value**	0.4924		0.6494		0.4202		0.8964	
**Q value**	0.743		NA		NA		NA	

AA pw Nef

	VRC-B		Cons.B		Anc.B		HXB2	
	Vaccine	Placebo	Vaccine	Placebo	Vaccine	Placebo	Vaccine	Placebo
**n**	27	20	27	20	27	20	27	20
**Median**	0.22	0.193	0.166	0.152	0.174	0.16	0.217	0.205
**Mean**	0.214	0.195	0.161	0.154	0.177	0.159	0.222	0.204
**P value**	0.0864		0.2774		0.1523		0.1345	
**Q value**	0.311		NA		NA		NA	

AA pw Rev

	Cons.B		Anc.B		HXB2	
	Vaccine	Placebo	Vaccine	Placebo	Vaccine	Placebo
**n**	27	20	27	20	27	20
**Median**	0.154	0.166	0.155	0.169	0.181	0.198
**Mean**	0.16	0.159	0.163	0.164	0.183	0.191
**P value**	0.9362		0.6134		0.4092	
**Q value**	NA		0.743		NA	

AA pw Tat

	Cons.B		Anc.B		HXB2	
	Vaccine	Placebo	Vaccine	Placebo	Vaccine	Placebo
**n**	27	20	27	20	27	20
**Median**	0.173	0.167	0.187	0.184	0.178	0.201
**Mean**	0.172	0.17	0.187	0.189	0.191	0.201
**P value**	0.8857		0.9531		0.5549	
	NA		0.953		NA	

AA pw Vif

	Cons.B		Anc.B		HXB2	
	Vaccine	Placebo	Vaccine	Placebo	Vaccine	Placebo
**n**	26	20	26	20	26	20
**Median**	0.107	0.123	0.116	0.123	0.13	0.14
**Mean**	0.111	0.12	0.113	0.119	0.129	0.138
**P value**	0.2373		0.433		0.2943	
**Q vlaue**	NA		0.743		NA	

AA pw Vpr

	Cons.B		Anc.B		HXB2	
	Vaccine	Placebo	Vaccine	Placebo	Vaccine	Placebo
**n**	26	20	26	20	26	20
**Median**	0.0926	0.091	0.0953	0.0909	0.118	0.116
**Mean**	0.0964	0.0923	0.0964	0.086	0.12	0.118
**P value**	0.777		0.303		0.8619	
	NA		0.727		NA	

AA pw Vpu

	Cons.B		Anc.B		HXB2	
	Vaccine	Placebo	Vaccine	Placebo	Vaccine	Placebo
**n**	27	20	27	20	27	20
**Median**	0.166	0.156	0.162	0.166	0.29	0.287
**Mean**	0.178	0.16	0.179	0.168	0.286	0.281
**P value**	0.3068		0.5241		0.8489	
**Q value**	NA		0.743		NA	

Table S5. Comparison of tree-based distance measures across treatment.

Mean divergence measures were calculated based on tree-based amino acid distances between the vaccine inserts or HIV-1 references and all sequences from a given subject. Comparisons between vaccine and placebo groups were done using a Wilcoxon rank sum test (Mann-Whitney test) with exact 2-sided p-value.

AA tb gp120

	VRC-A		VRC-B		VRC-C		Cons.B		Anc.B		MRCA		HXB2	
	Vaccine	Placebo	Vaccine	Placebo	Vaccine	Placebo	Vaccine	Placebo	Vaccine	Placebo	Vaccine	Placebo	Vaccine	Placebo
**n**	27	20	27	20	27	20	27	20	27	20	27	20	27	20
**Median**	0.914	0.89	0.682	0.657	1.12	1.1	0.557	0.532	0.576	0.551	0.478	0.454	0.67	0.645
**Mean**	0.92	0.906	0.682	0.663	1.12	1.11	0.556	0.538	0.576	0.558	0.486	0.47	0.67	0.652
**P value**	0.395		0.3383		0.4701		0.3383		0.3383		0.3276		0.3383	

AA tb gp41

	VRC-A		VRC-B		VRC-C		Cons.B		Anc.B		MRCA		HXB2	
	Vaccine	Placebo	Vaccine	Placebo	Vaccine	Placebo	Vaccine	Placebo	Vaccine	Placebo	Vaccine	Placebo	Vaccine	Placebo
**n**	27	20	27	20	27	20	27	20	27	20	27	20	27	20
**Median**	4.6	4.6	1.5	1.5	1.87	1.87	0.274	0.287	0.286	0.299	0.337	0.337	0.38	0.393
**Mean**	4.61	4.6	1.51	1.5	1.88	1.87	0.287	0.29	0.299	0.302	0.34	0.34	0.388	0.396
**P value**	0.6407		0.6407		0.6407		0.7216		0.7376		0.7058		0.6436	

AA tb Gag

	VRC-B		Cons.B		Anc.B		HXB2	
	Vaccine	Placebo	Vaccine	Placebo	Vaccine	Placebo	Vaccine	Placebo
**n**	26	20	26	20	26	20	26	20
**Median**	0.114	0.121	0.0996	0.107	0.115	0.122	0.123	0.13
**Mean**	0.114	0.123	0.101	0.109	0.116	0.124	0.124	0.132
**P value**	0.2647		0.3368		0.2647		0.2647	

AA tb Pol

	VRC-B		Cons.B		Anc.B		HXB2	
	Vaccine	Placebo	Vaccine	Placebo	Vaccine	Placebo	Vaccine	Placebo
**n**	26	20	26	20	26	20	26	20
**Median**	0.0903	0.0924	0.0728	0.0749	0.0804	0.0825	0.0996	0.102
**Mean**	0.0899	0.0926	0.0723	0.0751	0.0799	0.0827	0.0973	0.102
**P value**	0.446		0.446		0.446		0.3151	

AA tb Nef

	VRC-B		Cons.B		Anc.B		HXB2	
	Vaccine	Placebo	Vaccine	Placebo	Vaccine	Placebo	Vaccine	Placebo
**n**	27	20	27	20	27	20	27	20
**Median**	0.407	0.425	0.305	0.314	0.324	0.333	0.426	0.444
**Mean**	0.409	0.418	0.298	0.302	0.317	0.321	0.427	0.437
**P value**	0.7862		0.987		0.987		0.7862	

AA tb Rev

	Cons.B		Anc.B		HXB2	
	Vaccine	Placebo	Vaccine	Placebo	Vaccine	Placebo
**n**	27	20	27	20	27	20
**Median**	0.234	0.249	0.256	0.27	0.359	0.385
**Mean**	0.236	0.24	0.257	0.262	0.359	0.36
**P value**	0.6134		0.6134		0.6436	

AA tb Tat

	Cons.B		Anc.B		HXB2	
	Vaccine	Placebo	Vaccine	Placebo	Vaccine	Placebo
**n**	27	20	27	20	27	20
**Median**	0.277	0.272	0.318	0.313	0.307	0.303
**Mean**	0.285	0.28	0.326	0.321	0.329	0.318
**P value**	0.9835		0.9835		0.783	

AA tb Vif

	Cons.B		Anc.B		HXB2	
	Vaccine	Placebo	Vaccine	Placebo	Vaccine	Placebo
**n**	26	20	26	20	26	20
**Median**	0.168	0.169	0.17	0.174	0.217	0.217
**Mean**	0.168	0.175	0.168	0.176	0.215	0.223
**P value**	0.8307		0.6181		0.8137	

AA tb Vpr

	Cons.B		Anc.B		HXB2	
	Vaccine	Placebo	Vaccine	Placebo	Vaccine	Placebo
**n**	26	20	26	20	26	20
**Median**	0.185	0.154	0.267	0.239	0.228	0.198
**Mean**	0.186	0.153	0.255	0.221	0.229	0.196
**P value**	0.0633		0.1404		0.0633	

AA tb Vpu

	Cons.B		Anc.B		HXB2	
	Vaccine	Placebo	Vaccine	Placebo	Vaccine	Placebo
**n**	27	20	27	20	27	20
**Median**	0.335	0.354	0.378	0.397	0.633	0.603
**Mean**	0.381	0.336	0.424	0.377	0.63	0.596
**P value**	0.4967		0.4701		0.3171	

Table S6. Comparison pairwise and tree-based distance measures across treatment groups using Env-gp120 alignments without variable segments.

Distances correspond to the tree-based amino acid distance between the vaccine inserts or HIV-1 references and the breakthrough sequences from a given subject. Comparisons between vaccine and placebo groups were done using Mann-Whitney tests.

AA pw gp120 - Variable loops deleted

	VRC-A		VRC-B		VRC-C		Cons.B.04		Anc.B		MRCA		HXB2	
	Vaccine	Placebo	Vaccine	Placebo	Vaccine	Placebo	Vaccine	Placebo	Vaccine	Placebo	Vaccine	Placebo	Vaccine	Placebo
**n**	27	20	27	20	27	20	27	20	27	20	27	20	27	20
**Median**	0.300	0.289	0.222	0.210	0.341	0.328	0.159	0.138	0.161	0.144	0.264	0.249	0.210	0.192
**Mean**	0.303	0.292	0.229	0.208	0.346	0.331	0.158	0.139	0.160	0.148	0.267	0.249	0.214	0.196
**P value**	0.091		**0.004**		**0.015**		**0.014**		0.075		**0.007**		**0.023**	

AA tb gp120 - Variable loops deleted

	VRC-A		VRC-B		VRC-C		Cons.B.04		Anc.B		MRCA		HXB2	
	Vaccine	Placebo	Vaccine	Placebo	Vaccine	Placebo	Vaccine	Placebo	Vaccine	Placebo	Vaccine	Placebo	Vaccine	Placebo
**n**	27	20	27	20	27	20	27	20	27	20	27	20	27	20
**Median**	0.607	0.571	0.402	0.359	0.738	0.701	0.272	0.229	0.293	0.249	0.539	0.502	0.389	0.346
**Mean**	0.615	0.573	0.405	0.370	0.746	0.703	0.275	0.240	0.295	0.261	0.546	0.504	0.392	0.357
**P value**	**0.005**		**0.025**		**0.005**		**0.025**		**0.025**		**0.005**		**0.025**	

Table S7. Site scanning signatures.

Site and method for each site scanning result with a Q-value ≤ 0.2. N is the number of AA sites included for each multiple comparison. The Direction (+ for more or – for fewer mismatches in vaccine than placebo sequences) of the effect is with respect to the subtype B vaccine insert for Env, the vaccine insert for Pol and Nef and Ancestral B for Vif and Vpu. For Q-value and FWER adjustment, multiplicity adjustment was done at the gene level except where noted. One site, Pol 238, passes FWER adjustment (in bold) at the 0.05 level. ** Site/property combinations.

Site	Method	N	Direction	P-value	Q-value	Adjusted P-value*
Env 133	PCP (charged)	316**	+	0.00015	0.19	0.19
Env 429[$]	MBS	98	+	0.0014	0.15	0.15
Pol 238	GWJ	180	−	0.0006	0.11	0.11
Pol 238	EGWJ	245	−	0.0002	0.05	**0.05**
Pol 238	QEMD	180	−	0.0006	0.11	0.11
Nef 83	EGWJ	94	+	0.0014	0.13	0.13
Vif 122	MBS	65	+	0.001	0.07	0.07
Vpu 69[#]	QEMD	65	−	0.0018	0.08	0.08
Vpu 69a[#]	QEMD	65	−	0.013	0.15	0.55
Vpu 69b[#]	QEMD	65	−	0.013	0.15	0.55
Vpu 69c[#]	QEMD	65	−	0.013	0.15	0.55

[#]Vpu69-69c signatures are due to insertions in 6 placebo recipient breakthrough *mindist* sequences of between 1 and 4 amino acids.

[$]Multiplicity adjustment is by gene except for Env 429, which is over the set of mAb contact sites.

Table S8. HIV-1 Env sites under positive selection in the vaccine group only.
Eight sites were identified in Env-gp120 using three methods.

					SLAC		IFEL		MEME		
**Position**	**HXB2**	**VRC-B**	**Vaccine**	**Placebo**	**dN-dS**	**p-value**	**dN-dS**	**p-value**	**Log(L)**	**p-value**	**q-value**
147	M	M	K	T	38.44	0.049	6.29	0.000	-138.11	0.000	0.015
183	P	P	P	P	36.30	0.017	0.60	0.003	-49.47	0.002	0.065
236	T	K	T	T	45.99	0.011	0.76	0.002	-67.05	0.004	0.093
290	T	E	E	E	47.16	0.033	0.72	0.039	-74.34	0.007	0.141
315	R	R	R	R	42.41	0.032	0.73	0.010	-68.88	0.007	0.132
354	G	G	E	E	53.71	0.034	1.51	0.033	-94.55	0.000	0.035
388	T	T	T	T	35.18	0.014	0.48	0.007	-50.55	0.010	0.158
442	Q	Q	Q	Q	65.22	0.013	1.94	0.012	-106.19	0.006	0.122

Table S9. Percentage of CTL epitopes predicted among breakthrough sequences that were matched to HIV-1 reference sequences.
Epitopes predicted in breakthrough sequences were matched against epitopes derived from reference sequences when there were no more than 3 mutations between the 9mers.

Protein	Reference	Subtype	Matched epitopes (%)
Gag	VRC4401	B	84.52
	B_Anc	B	83.68
	B_Con	B	85.44
	HXB2	B	84.52
Pol	VRC4409	B	86.97
	B_Anc	B	89.00
	B_Con	B	89.30
	HXB2	B	87.69
Env-gp120	ad5_gp140a	A	45.27
	ad5_gp140b	B	50.19
	ad5_gp140c	C	42.74
	VRC5736_GP145A	A	45.27
	VRC5737_GP145B	B	55.76
	VRC5738_GP145C	C	42.74
	B_Anc	B	63.52
	B_Con	B	65.13
	HXB2	B	58.13
Nef	VRC4404	B	63.38
	B_Anc	B	69.46
	B_Con	B	70.66
	HXB2	B	60.00
Rev	B_Anc	B	59.10
	B_Con	B	59.13
	HXB2	B	52.03
Tat	B_Anc	B	59.46
	B_Con	B	57.52
	HXB2	B	58.25
Vif	B_Anc	B	80.17

Protein	Reference	Subtype	Matched epitopes (%)
Env-gp120	ad5_gp140c	C	42.74
Env-gp120	VRC5738_GP145C	C	42.74
Vpu	HXB2	B	43.82
Env-gp120	ad5_gp140a	A	45.27
Env-gp120	VRC5736_GP145A	A	45.27
Env-gp120	ad5_gp140b	B	50.19
Rev	HXB2	B	52.03
Env-gp120	VRC5737_GP145B	B	55.76
Tat	B_Con	B	57.52
Env-gp120	HXB2	B	58.13
Tat	HXB2	B	58.25
Rev	B_Anc	B	59.10
Rev	B_Con	B	59.13
Tat	B_Anc	B	59.46
Nef	HXB2	B	60.00
Nef	VRC4404	B	63.38
Env-gp120	B_Anc	B	63.52
Env-gp120	B_Con	B	65.13
Vpu	B_Con	B	66.96
Vpu	B_Anc	B	67.18
Nef	B_Anc	B	69.46
Nef	B_Con	B	70.66
Vpr	HXB2	B	73.83
Vif	HXB2	B	77.02
Vif	B_Anc	B	80.17
Vif	B_Con	B	81.67
Vpr	B_Con	B	83.24
Gag	B_Anc	B	83.68
Gag	VRC4401	B	84.52
Gag	HXB2	B	84.52
Gag	B_Con	B	85.44
Vpr	B_Anc	B	85.85
Pol	VRC4409	B	86.97
Pol	HXB2	B	87.69

	B_Con	B	81.67
	HXB2	B	77.02
Vpr	B_Anc	B	85.85
	B_Con	B	83.24
	HXB2	B	73.83
Vpu	B_Anc	B	67.18
	B_Con	B	66.96
	HXB2	B	43.82

Pol	B_Anc	B	89.00
Pol	B_Con	B	89.30

Table S10. Comparison of binding affinity measures for predicted CTL epitopes from vaccine and placebo recipients. Epitopes predicted to be strong and weak binders were matched against vaccine inserts or HIV-1 reference sequences and the predicted binding affinity of breakthrough virus-derived epitopes were compared to those of vaccine- and reference-derived epitopes. The distribution of summary values determined for each subject was compared between vaccine and placebo groups using Mann-Whitney tests.

BINDING AFFINITY - STRONG AND WEAK BINDERS

Env-gp120

	**VRC-A**		**VRC-B**		**VRC-C**		**Cons.B**		**Anc.B**		**HXB2**	
	**Vaccine**	**Placebo**	**Vaccine**	**Placebo**	**Vaccine**	**Placebo**	**Vaccine**	**Placebo**	**Vaccine**	**Placebo**	**Vaccine**	**Placebo**
**n**	25	18	25	18	25	18	25	18	25	18	25	18
**Median**	1.117	1.311	1.179	1.413	1.198	1.220	1.215	1.182	1.488	1.279	1.211	1.326
**Mean**	1.261	1.537	1.208	1.396	1.404	1.317	1.349	1.418	1.630	1.913	1.223	1.417
**P value**	0.056		0.118		0.966		0.813		0.809		0.234	

Env-gp41

	**VRC-A**		**VRC-B**		**VRC-C**		**Cons.B**		**Anc.B**		**HXB2**	
	**Vaccine**	**Placebo**	**Vaccine**	**Placebo**	**Vaccine**	**Placebo**	**Vaccine**	**Placebo**	**Vaccine**	**Placebo**	**Vaccine**	**Placebo**
**n**	25	18	25	18	25	18	25	18	25	18	25	18
**Median**	0.980	1.468	1.320	1.605	1.198	1.100	1.570	1.206	1.389	1.196	1.330	1.339
**Mean**	1.260	1.810	1.456	1.634	2.054	2.159	1.519	1.431	1.391	1.305	1.403	1.379
**P value**	**0.019**		0.345		0.981		0.150		0.319		0.753	

Gag

	**VRC-B**		**Cons.B**		**Anc.B**		**HXB2**	
	**Vaccine**	**Placebo**	**Vaccine**	**Placebo**	**Vaccine**	**Placebo**	**Vaccine**	**Placebo**
**n**	24	18	24	18	24	18	24	18
**Median**	1.114	1.115	1.096	1.089	1.097	1.062	1.098	1.083
**Mean**	1.253	1.132	1.225	1.110	1.208	1.077	1.248	1.113
**P value**	0.367		0.452		0.228		0.452	

Pol

	**VRC-B**		**Cons.B**		**Anc.B**		**HXB2**	
	**Vaccine**	**Placebo**	**Vaccine**	**Placebo**	**Vaccine**	**Placebo**	**Vaccine**	**Placebo**
**n**	24	18	24	18	24	18	24	18
**Median**	1.137	1.173	1.099	1.107	1.136	1.110	1.104	1.099
**Mean**	1.194	1.242	1.162	1.193	1.171	1.192	1.183	1.218
**P value**	0.249		0.693		0.862		0.905	

Nef

	VRC-B Vaccine	Placebo	Cons.B Vaccine	Placebo	Anc.B Vaccine	Placebo	HXB2 Vaccine	Placebo
**n**	25	18	25	18	25	18	25	18
**Median**	1.339	1.099	1.174	1.010	1.354	1.088	1.364	1.138
**Mean**	1.730	1.585	1.509	1.278	1.670	1.282	1.747	1.573
**P value**	0.560		**0.049**		**0.039**		0.497	

Rev

	Cons.B Vaccine	Placebo	Anc.B Vaccine	Placebo	HXB2 Vaccine	Placebo
**n**	25	16	25	17	25	16
**Median**	1.578	1.508	1.632	1.531	1.250	1.173
**Mean**	3.241	2.082	2.974	2.151	3.372	2.161
**P value**	0.922		0.934		0.752	

Tat

	Cons.B Vaccine	Placebo	Anc.B Vaccine	Placebo	HXB2 Vaccine	Placebo
**n**	17	9	17	9	17	9
**Median**	1.000	1.000	1.000	1.000	1.446	1.362
**Mean**	1.240	1.036	1.424	1.163	1.760	1.286
**P value**	0.676		0.735		0.269	

Vif

	Cons.B Vaccine	Placebo	Anc.B Vaccine	Placebo	HXB2 Vaccine	Placebo
**n**	24	18	24	18	24	18
**Median**	1.130	1.129	1.141	1.126	1.365	1.199
**Mean**	1.679	1.371	1.656	1.353	1.792	1.435
**P value**	0.965		0.822		0.269	

Vpr

	Cons.B Vaccine	Placebo	Anc.B Vaccine	Placebo	HXB2 Vaccine	Placebo
**n**	24	18	24	18	24	18
**Median**	1.061	1.002	1.101	1.002	0.994	0.993
**Mean**	1.171	1.114	1.226	1.113	1.078	1.107
**P value**	0.315		0.191		0.921	

Vpu

	Cons.B Vaccine	Placebo	Anc.B Vaccine	Placebo	HXB2 Vaccine	Placebo
**n**	25	18	25	18	25	18
**Median**	1.312	1.186	1.291	1.149	1.112	1.080
**Mean**	2.101	2.414	2.080	3.022	3.698	1.219
**P value**	0.295		0.645		0.476	

Table S11. Comparison of evolutionary distances for predicted CTL epitopes from vaccine and placebo recipients. Epitopes predicted to be strong and weak binders were matched against vaccine inserts or HIV-1 reference sequences and evolutionary distances were computed between breakthrough virus-derived epitopes and vaccine or reference-derived epitopes. The distribution of summary values determined for each subject was compared between vaccine and placebo groups using Mann-Whitney tests.

EVOLUTIONARY DISTANCES - STRONG AND WEAK BINDERS

Env-gp120

	VRC-A		VRC-B		VRC-C		Cons.B		Anc.B		HXB2	
	Vaccine	Placebo	Vaccine	Placebo	Vaccine	Placebo	Vaccine	Placebo	Vaccine	Placebo	Vaccine	Placebo
n	25	18	25	18	25	18	25	18	25	18	25	18
Median	0.253	0.229	0.170	0.176	0.236	0.253	0.142	0.154	0.147	0.149	0.155	0.155
Mean	0.244	0.233	0.179	0.185	0.237	0.255	0.146	0.144	0.155	0.150	0.161	0.158
P value	0.295		0.580		0.206		0.832		0.716		0.716	

Env-gp41

	VRC-A		VRC-B		VRC-C		Cons.B		Anc.B		HXB2	
	Vaccine	Placebo	Vaccine	Placebo	Vaccine	Placebo	Vaccine	Placebo	Vaccine	Placebo	Vaccine	Placebo
n	25	18	25	18	25	18	25	18	25	18	25	18
Median	0.230	0.282	0.228	0.229	0.296	0.220	0.160	0.165	0.172	0.156	0.185	0.167
Mean	0.247	0.297	0.223	0.228	0.277	0.226	0.164	0.169	0.158	0.157	0.177	0.180
P value	0.066		> 0.999		**0.014**		0.889		0.771		0.885	

Gag

	VRC-B		Cons.B		Anc.B		HXB2	
	Vaccine	Placebo	Vaccine	Placebo	Vaccine	Placebo	Vaccine	Placebo
n	24	18	24	18	24	18	24	18
Median	0.109	0.101	0.091	0.099	0.100	0.118	0.110	0.100
Mean	0.108	0.109	0.097	0.101	0.099	0.114	0.115	0.110
P value	0.925		0.807		0.091		0.746	

Pol

	VRC-B		Cons.B		Anc.B		HXB2	
	Vaccine	Placebo	Vaccine	Placebo	Vaccine	Placebo	Vaccine	Placebo
n	24	18	24	18	24	18	24	18
Median	0.087	0.073	0.068	0.061	0.071	0.068	0.082	0.067
Mean	0.087	0.077	0.070	0.064	0.072	0.067	0.085	0.071
P value	0.090		0.137		0.158		**0.016**	

Nef

	VRC-B		Cons.B		Anc.B		HXB2	
	Vaccine	Placebo	Vaccine	Placebo	Vaccine	Placebo	Vaccine	Placebo
**n**	25	18	25	18	25	18	25	18
**Median**	0.231	0.216	0.125	0.125	0.147	0.124	0.260	0.226
**Mean**	0.238	0.206	0.137	0.138	0.163	0.137	0.258	0.219
**P value**	0.123		0.885		0.188		0.069	

Rev

	Cons.B		Anc.B		HXB2	
	Vaccine	Placebo	Vaccine	Placebo	Vaccine	Placebo
**n**	25	16	25	17	25	16
**Median**	0.231	0.226	0.232	0.218	0.341	0.258
**Mean**	0.262	0.242	0.257	0.231	0.306	0.306
**P value**	0.580		0.505		0.874	

Tat

	Cons.B		Anc.B		HXB2	
	Vaccine	Placebo	Vaccine	Placebo	Vaccine	Placebo
**n**	17	9	17	9	17	9
**Median**	0.100	0.000	0.024	0.000	0.283	0.202
**Mean**	0.102	0.076	0.099	0.091	0.261	0.228
**P value**	0.557		0.452		0.301	

Vif

	Cons.B		Anc.B		HXB2	
	Vaccine	Placebo	Vaccine	Placebo	Vaccine	Placebo
**n**	24	18	24	18	24	18
**Median**	0.118	0.143	0.129	0.140	0.144	0.145
**Mean**	0.127	0.131	0.127	0.129	0.143	0.146
**P value**	0.768		0.768		0.885	

Vpr

	Cons.B		Anc.B		HXB2	
	Vaccine	Placebo	Vaccine	Placebo	Vaccine	Placebo
**n**	24	18	24	18	24	18
**Median**	0.102	0.083	0.112	0.087	0.125	0.091
**Mean**	0.119	0.084	0.124	0.096	0.131	0.098
**P value**	**0.037**		0.209		**0.040**	

Vpu

	Cons.B		Anc.B		HXB2	
	Vaccine	Placebo	Vaccine	Placebo	Vaccine	Placebo
**n**	25	18	25	18	25	18
**Median**	0.231	0.232	0.228	0.227	0.323	0.210
**Mean**	0.230	0.197	0.229	0.225	0.299	0.218
**P value**	0.528		0.847		0.057	

Table S12. Comparison of binding affinity measures for predicted CTL epitopes (strong binders only) from vaccine and placebo recipients.

Epitopes predicted to be strong binders were matched against vaccine inserts or reference sequences and the predicted binding affinity of breakthrough virus-derived epitopes were compared to those of vaccine- or reference-derived epitopes. The distribution of summary values determined for each subject was compared between vaccine and placebo groups using Mann-Whitney tests.

BINDING AFFINITY - STRONG BINDERS

Env-gp120

	VRC-A		VRC-B		VRC-C		Cons.B		Anc.B		HXB2	
	Vaccine	Placebo	Vaccine	Placebo	Vaccine	Placebo	Vaccine	Placebo	Vaccine	Placebo	Vaccine	Placebo
n	25	18	25	18	25	18	25	18	25	18	25	18
Median	0.758	0.770	0.860	0.840	0.862	0.965	0.956	0.909	0.960	1.035	0.868	0.901
Mean	0.805	0.868	0.881	1.018	0.890	1.016	0.941	1.051	1.049	1.181	0.896	1.037
P value	0.516		0.947		0.345		0.735		0.398		0.966	

Env-gp41

	VRC-A		VRC-B		VRC-C		Cons.B		Anc.B		HXB2	
	Vaccine	Placebo	Vaccine	Placebo	Vaccine	Placebo	Vaccine	Placebo	Vaccine	Placebo	Vaccine	Placebo
n	25	18	25	18	24	18	25	18	25	18	25	18
Median	0.692	0.774	0.840	0.937	0.938	0.875	0.904	0.986	0.905	0.991	0.875	0.939
Mean	0.708	0.854	0.880	1.008	0.923	0.904	0.953	1.020	0.939	1.053	0.902	0.977
P value	0.350		0.485		0.875		0.455		0.275		0.265	

Gag

	VRC-B		Cons.B		Anc.B		HXB2	
	Vaccine	Placebo	Vaccine	Placebo	Vaccine	Placebo	Vaccine	Placebo
n	24	18	24	18	24	18	24	18
Median	1.025	1.030	0.998	0.998	0.916	0.969	0.990	0.996
Mean	1.108	1.078	1.075	1.044	1.038	0.995	1.078	1.035
P value	0.826		0.693		0.620		> 0.999	

Pol

	VRC-B		Cons.B		Anc.B		HXB2	
	Vaccine	Placebo	Vaccine	Placebo	Vaccine	Placebo	Vaccine	Placebo
n	24	18	24	18	24	18	24	18
Median	0.934	0.942	0.943	0.967	0.951	0.969	0.970	0.954
Mean	0.953	0.984	0.960	0.991	0.965	0.993	0.977	0.985
P value	0.885		> 0.999		0.945		0.565	

Nef

	VRC-B		Cons.B		Anc.B		HXB2	
	Vaccine	Placebo	Vaccine	Placebo	Vaccine	Placebo	Vaccine	Placebo
**n**	24	18	24	17	24	18	24	17
**Median**	0.801	0.993	1.000	0.895	1.022	0.940	0.793	0.982
**Mean**	0.876	0.947	1.006	0.872	1.127	0.875	0.867	0.926
**P value**	0.283		**0.006**		**0.004**		0.394	

Rev

	Cons.B		Anc.B		HXB2	
	Vaccine	Placebo	Vaccine	Placebo	Vaccine	Placebo
**n**	18	8	18	8	17	7
**Median**	0.635	0.708	0.510	0.817	0.723	0.937
**Mean**	0.777	1.730	0.828	1.871	0.893	1.236
**P value**	0.196		0.126		0.126	

Tat

	Cons.B		Anc.B		HXB2	
	Vaccine	Placebo	Vaccine	Placebo	Vaccine	Placebo
**n**	3	2	3	2	2	2
**Median**	1.000	0.557	1.000	1.000	1.801	0.695
**Mean**	0.684	0.557	0.823	1.000	1.801	0.695
**P value**	> 0.999		> 0.999		0.667	

Vif

	Cons.B		Anc.B		HXB2	
	Vaccine	Placebo	Vaccine	Placebo	Vaccine	Placebo
**n**	24	18	24	18	24	18
**Median**	0.839	0.919	0.835	0.924	0.930	0.912
**Mean**	0.847	0.927	0.809	0.926	0.906	0.957
**P value**	0.383		0.125		0.469	

Vpr

	Cons.B		Anc.B		HXB2	
	Vaccine	Placebo	Vaccine	Placebo	Vaccine	Placebo
**n**	22	16	22	16	22	16
**Median**	0.821	0.855	1.004	0.954	0.856	0.849
**Mean**	0.963	0.833	1.161	0.965	0.955	0.828
**P value**	0.844		0.685		0.731	

Vpu

	Cons.B		Anc.B		HXB2	
	Vaccine	Placebo	Vaccine	Placebo	Vaccine	Placebo
**n**	22	14	21	14	16	9
**Median**	0.615	0.942	0.572	0.806	0.481	0.894
**Mean**	0.877	0.841	0.847	0.720	0.819	0.905
**P value**	0.516		0.727		0.427	

Table S13. Comparison of evolutionary distances for predicted CTL epitopes (strong binders only) from vaccine and placebo recipients.

Epitopes predicted to be strong binders were matched against vaccine inserts or HIV-1 reference sequences and evolutionary distances were computed between breakthrough virus-derived epitopes and vaccine- or reference-derived epitopes. The distribution of summary values determined for each subject was compared between vaccine and placebo groups using Mann-Whitney tests.

EVOLUTIONARY DISTANCES - STRONG BINDERS

Env-gp120

	VRC-A		VRC-B		VRC-C		Cons.B		Anc.B		HXB2	
	Vaccine	Placebo	Vaccine	Placebo	Vaccine	Placebo	Vaccine	Placebo	Vaccine	Placebo	Vaccine	Placebo
**n**	25	18	25	18	25	18	25	18	25	18	25	18
**Median**	0.262	0.259	0.210	0.210	0.270	0.251	0.197	0.173	0.191	0.170	0.228	0.197
**Mean**	0.273	0.249	0.221	0.223	0.275	0.271	0.194	0.172	0.195	0.167	0.219	0.204
**P value**	0.356		0.966		0.809		0.382		0.233		0.466	

Env-gp41

	VRC-A		VRC-B		VRC-C		Cons.B		Anc.B		HXB2	
	Vaccine	Placebo	Vaccine	Placebo	Vaccine	Placebo	Vaccine	Placebo	Vaccine	Placebo	Vaccine	Placebo
**n**	25	18	25	18	24	18	25	18	25	18	25	18
**Median**	0.270	0.254	0.194	0.207	0.271	0.216	0.156	0.174	0.167	0.167	0.188	0.183
**Mean**	0.266	0.278	0.252	0.212	0.250	0.224	0.160	0.175	0.163	0.169	0.189	0.190
**P value**	0.860		0.692		0.567		0.500		0.908		0.885	

Gag

	VRC-B		Cons.B		Anc.B		HXB2	
	Vaccine	Placebo	Vaccine	Placebo	Vaccine	Placebo	Vaccine	Placebo
**n**	24	18	24	18	24	18	24	18
**Median**	0.139	0.133	0.113	0.119	0.141	0.146	0.161	0.133
**Mean**	0.142	0.170	0.128	0.148	0.141	0.151	0.157	0.172
**P value**	0.730		0.674		0.885		0.862	

Pol

	VRC-B		Cons.B		Anc.B		HXB2	
	Vaccine	Placebo	Vaccine	Placebo	Vaccine	Placebo	Vaccine	Placebo
**n**	24	18	24	18	24	18	24	18
**Median**	0.104	0.077	0.073	0.070	0.079	0.069	0.081	0.072
**Mean**	0.104	0.088	0.077	0.075	0.083	0.080	0.087	0.078
**P value**	0.061		0.862		0.708		0.532	

Nef

	VRC-B		Cons.B		Anc.B		HXB2	
	Vaccine	Placebo	Vaccine	Placebo	Vaccine	Placebo	Vaccine	Placebo
**n**	24	18	24	17	24	18	24	17
**Median**	0.269	0.222	0.146	0.126	0.181	0.128	0.275	0.229
**Mean**	0.259	0.209	0.145	0.144	0.172	0.151	0.268	0.216
**P value**	0.137		0.922		0.985		0.149	

Rev

	Cons.B		Anc.B		HXB2	
	Vaccine	Placebo	Vaccine	Placebo	Vaccine	Placebo
**n**	18	8	18	8	17	7
**Median**	0.280	0.319	0.301	0.312	0.365	0.408
**Mean**	0.281	0.336	0.291	0.324	0.336	0.376
**P value**	0.495		0.523		0.619	

Tat

	Cons.B		Anc.B		HXB2	
	Vaccine	Placebo	Vaccine	Placebo	Vaccine	Placebo
**n**	3	2	3	2	2	2
**Median**	0.000	0.080	0.000	0.000	0.203	0.338
**Mean**	0.139	0.080	0.131	0.000	0.203	0.338
**P value**	> 0.999		> 0.999		> 0.999	

Vif

	Cons.B		Anc.B		HXB2	
	Vaccine	Placebo	Vaccine	Placebo	Vaccine	Placebo
**n**	24	18	24	18	24	18
**Median**	0.136	0.103	0.150	0.103	0.121	0.102
**Mean**	0.144	0.114	0.151	0.115	0.133	0.112
**P value**	0.315		0.182		0.533	

Vpr

	Cons.B		Anc.B		HXB2	
	Vaccine	Placebo	Vaccine	Placebo	Vaccine	Placebo
**n**	22	16	22	16	22	16
**Median**	0.212	0.203	0.219	0.233	0.216	0.230
**Mean**	0.211	0.215	0.215	0.244	0.231	0.228
**P value**	0.820		0.372		0.843	

Vpu

	Cons.B		Anc.B		HXB2	
	Vaccine	Placebo	Vaccine	Placebo	Vaccine	Placebo
**n**	22	14	21	14	16	9
**Median**	0.273	0.253	0.310	0.284	0.365	0.222
**Mean**	0.292	0.260	0.285	0.299	0.361	0.296
**P value**	0.548		0.829		0.335	

Table S14. Comparison between the vaccine and placebo groups for 19 Env-gp120 CTL epitopes. Only epitopes that were identified in at least three vaccine and three placebo recipients were considered for comparison through Mann-Whitney tests. Mean values corresponding to all subjects in each group are reported; for a given subject, the binding affinity or evolutionary distance corresponds to the comparison of the breakthrough virus-derived epitope to the corresponding epitope in the subtype B vaccine.

			**N. subjects**		**Binding affinity**			**Evolutionary distance**		
**Position**	**HLA**	**Epitope**	**Vaccine**	**Placebo**	**Vaccine**	**Placebo**	**p-value**	**Vaccine**	**Placebo**	**p-value**
18	A*02:01	TMLLGMLMI	15	6	0.720	0.672	0.953	0.167	0.213	0.775
**35**	**A*02:01**	**VTVYYGVPV**	**12**	**7**	**1.000**	**0.823**	**0.282**	**0.000**	**0.096**	**0.036**
66	A*02:01	NVWATHACV	12	5	1.149	1.000	> 0.999	0.007	0.000	> 0.999
102	A*02:01	QMHEDIISL	13	8	1.309	1.598	0.919	0.120	0.175	0.345
120	A*02:01	KLTPLCVSL	13	7	1.711	2.602	0.597	0.184	0.169	> 0.999
175	C*06:02	FYKLDIIPI	4	4	0.671	0.971	0.057	0.505	0.480	0.743
191	A*02:01	SLTSCNTSV	10	8	1.732	0.964	0.446	0.442	0.387	0.823
250	C*06:02	IRPVVSTQL	3	3	1.000	1.000	> 0.999	0.000	0.000	> 0.999
307	A*01:01	HIGPGRAFY	6	3	0.795	0.965	0.191	0.091	0.437	0.179
372	A*24:02	SFNCGGEFF	3	4	1.025	1.057	0.486	0.041	0.093	0.486
373	A*01:01	FNCGGEFFY	7	4	0.968	0.849	0.746	0.032	0.066	0.746
379	C*06:02	FFYCNSTQL	7	6	0.636	1.130	0.586	0.152	0.259	0.159
**379**	**C*07:01**	**FFYCNSTQL**	**11**	**4**	**0.817**	**0.427**	**0.018**	**0.259**	**0.288**	**0.686**
380	A*24:02	FYCNSTQLF	3	6	1.671	2.430	0.798	0.290	0.189	0.286
380	C*06:02	FYCNSTQLF	7	5	0.965	0.770	0.303	0.152	0.277	0.165
415	C*06:02	CRIKQIINM	5	4	0.980	1.241	0.651	0.111	0.313	0.389
442	A*01:01	CSSNITGLL	5	5	1.188	1.065	> 0.999	0.073	0.137	0.722
443	A*01:01	SSNITGLLL	4	3	1.000	1.000	> 0.999	0.000	0.000	> 0.999
472	C*06:02	MRDNWRSEL	4	6	1.000	1.083	> 0.999	0.000	0.030	> 0.999

Table S15. Monoclonal antibody contact set scanning.

Results of the mAb contact set scanning analysis grouped by monoclonal antibody class (CD4bs, CD4i, and V3, Quarternary, gp41 MPER, gp41 NHR, gp41 cluster II, and Glycan) and ranked within class by P-value. The effect size is measured in additional mismatch rate in the vaccine group per contact site where a positive (negative) value indicates more (fewer) mismatches at contact site residues in breakthrough sequences for the vaccine group as compared to the placebo group and **n** is the number of cantact sites. For results with a Q-value ≤ 0.2 the mAb name is in bold.

	mAb	Effect	n	P-value	Q-value
**CD4bs**	**CD4**	4.9%	48	0.00079	0.035
	**F105**	5.2%	36	0.00289	0.039
	**b12**	5.8%	32	0.00311	0.039
	**VRC-PG20**	4.2%	44	0.00431	0.039
	**12A12**	3.9%	47	0.00448	0.039
	**NIH45-46**	3.5%	48	0.01209	0.072
	**VRC03**	3.7%	43	0.01257	0.072
	**b13**	5.9%	26	0.01479	0.072
	**CH103**	5.1%	27	0.01642	0.072
	**VRC06**	3.2%	46	0.02000	0.078
	**3BNC117**	3.6%	39	0.02210	0.078
	**VRC23**	3.5%	43	0.02298	0.078
	**VRC-PG04**	3.9%	32	0.02881	0.082
	**VRC01**	3.8%	37	0.02933	0.082
	**CH31**	4.4%	31	0.03382	0.086
**CD4i**	**412d**	2.0%	37	0.03514	0.086
	**X5**	2.5%	25	0.06689	0.155
	17b	2.0%	19	0.11406	0.251
	48d	1.7%	23	0.13082	0.274
	21c	1.9%	25	0.22526	0.411
**V3**	**PGT128**	5.3%	12	0.01483	0.072
	**PGT122**	4.9%	15	0.02997	0.082
	4025	4.0%	14	0.21402	0.411
	537-10D	3.9%	13	0.21777	0.411
	2558	3.3%	15	0.23380	0.411
	R20	3.2%	9	0.25528	0.432
	1006-15D	3.2%	13	0.30330	0.440
	2219	2.9%	13	0.30920	0.440
	268-D	3.2%	11	0.30943	0.440
	3074	3.3%	14	0.30967	0.440
	R56	2.1%	10	0.44015	0.605
	F425-B4e8	2.8%	11	0.46506	0.620
	447-52D	2.1%	9	0.54235	0.645
	PGT135	1.3%	19	0.59721	0.661
**Quaternary**	PG16	1.1%	13	0.72926	0.783
	PG9	0.2%	17	0.92635	0.948
**gp41 MPER**	10E8	2.0%	14	0.30750	0.440
	4E10	1.5%	10	0.52193	0.638
	Z13e1	1.8%	8	0.52193	0.638
	2F5	-2.0%	7	0.58217	0.661
	m66	0.1%	13	0.96003	0.960
**gp41 NHR**	D5	-0.9%	24	0.52192	0.638
**gp41 cluster II**	1281	0.5%	16	0.84981	0.890
**Glycan**	2G12	2.0%	6	0.60059	0.661

Table S16: Results of machine learning sieve analysis.

Results for the area under the ROC curve (AUC) and classification accuracy (ACC) on held-out data for the four machine learning methods applied over the different regions of the HIV-1 genome. The "region" column indicates the region of the protein for which AA sites were included in the analysis. A null result with no classification capacity is reflected by AUC ≤ 0.5 and ACC ≤ 0.574 (= 27/47).

Protein	Region	Measure	LASSO	LogitBoost	Naive Bayes	Random Forest
Env	Monoclonal Ab sites (p = 236 AA sites)	AUC	0.481	0.475	0.551	0.528
		ACC	0.616	0.610	0.624	0.624
	IgG Binding Hotspots (p = 44 AA sites)	AUC	0.464	0.481	0.524	0.511
		ACC	0.605	0.609	0.606	0.617
	V3 (p=77 AA sites)	AUC	0.482	0.514	0.505	**0.616**
		ACC	0.617	0.619	0.600	**0.661**
	CD4 binding site (p=93 AA sites)	AUC	0.504	0.494	0.538	0.487
		ACC	0.622	0.608	0.610	0.609
	gp145	AUC	0.490	0.514	0.532	0.525
		ACC	0.628	0.616	0.630	0.613
Gag	full protein	AUC	0.453	0.474	0.502	0.473
		ACC	0.596	0.607	0.603	0.598
Pol	full protein	AUC	0.537	0.541	0.541	0.521
		ACC	0.650	0.632	0.618	0.606
Nef	full protein	AUC	0.527	0.524	0.481	0.465
		ACC	0.641	0.626	0.604	0.604
Env	non insert	AUC	0.474	0.469	0.508	0.468
		ACC	0.620	0.608	0.605	0.607
Rev	full protein	AUC	0.513	0.529	0.520	0.530
		ACC	0.626	0.625	0.599	0.623
Tat	full protein	AUC	0.476	0.515	0.494	0.506
		ACC	0.616	0.621	0.608	0.615
Vif	full protein	AUC	0.496	0.524	0.549	0.599
		ACC	0.628	0.621	0.611	0.646
Vpr	full protein	AUC	0.468	0.477	0.537	0.548
		ACC	0.609	0.602	0.611	0.622
Vpu	full protein	AUC	0.449	0.433	0.488	0.389
		ACC	0.605	0.608	0.607	0.603
All Proteins	everything	AUC	0.513	0.497	0.533	0.511
		ACC	0.632	0.607	0.621	0.606
