# Supplementary material for: Sieve analysis of breakthrough HIV-1 sequences in HVTN 505 identifies vaccine pressure targeting the CD4 binding site of Env-gp120
Source: PLoS One. 2017 Nov 17;12(11):e0185959. doi: 10.1371/journal.pone.0185959 (PMC5693417; doi:10.1371/journal.pone.0185959)
Supplement: S9 Table — Epitopes predicted in breakthrough sequences were matched against epitopes derived from reference sequences when there were no more than 3 mutations between the 9mers. (PDF) [file pone.0185959.s009.pdf]

Table S9. Percentage of CTL epitopes predicted among breakthrough sequences that were matched to HIV-1 reference sequences. Epitopes predicted in breakthrough sequences were matched against epitopes derived from reference sequences when there were no more than 3 mutations between the 9mers.

Protein	Reference	Subtype	Matched epitopes (%)
Gag	VRC4401	B	84.52
	B_Anc	B	83.68
	B_Con	B	85.44
	HXB2	B	84.52
Pol	VRC4409	B	86.97
	B_Anc	B	89.00
	B_Con	B	89.30
	HXB2	B	87.69
Env-gp120	ad5_gp140a	A	45.27
	ad5_gp140b	B	50.19
	ad5_gp140c	C	42.74
	VRC5736_GP145A	A	45.27
	VRC5737_GP145B	B	55.76
	VRC5738_GP145C	C	42.74
	B_Anc	B	63.52
	B_Con	B	65.13
	HXB2	B	58.13
Nef	VRC4404	B	63.38
	B_Anc	B	69.46
	B_Con	B	70.66
	HXB2	B	60.00
Rev	B_Anc	B	59.10
	B_Con	B	59.13
	HXB2	B	52.03
Tat	B_Anc	B	59.46
	B_Con	B	57.52
	HXB2	B	58.25
Vif	B_Anc	B	80.17

Protein	Reference	Subtype	Matched epitopes (%)
Env-gp120	ad5_gp140c	C	42.74
Env-gp120	VRC5738_GP145C	C	42.74
Vpu	HXB2	B	43.82
Env-gp120	ad5_gp140a	A	45.27
Env-gp120	VRC5736_GP145A	A	45.27
Env-gp120	ad5_gp140b	B	50.19
Rev	HXB2	B	52.03
Env-gp120	VRC5737_GP145B	B	55.76
Tat	B_Con	B	57.52
Env-gp120	HXB2	B	58.13
Tat	HXB2	B	58.25
Rev	B_Anc	B	59.10
Rev	B_Con	B	59.13
Tat	B_Anc	B	59.46
Nef	HXB2	B	60.00
Nef	VRC4404	B	63.38
Env-gp120	B_Anc	B	63.52
Env-gp120	B_Con	B	65.13
Vpu	B_Con	B	66.96
Vpu	B_Anc	B	67.18
Nef	B_Anc	B	69.46
Nef	B_Con	B	70.66
Vpr	HXB2	B	73.83
Vif	HXB2	B	77.02
Vif	B_Anc	B	80.17
Vif	B_Con	B	81.67
Vpr	B_Con	B	83.24
Gag	B_Anc	B	83.68
Gag	VRC4401	B	84.52
Gag	HXB2	B	84.52
Gag	B_Con	B	85.44
Vpr	B_Anc	B	85.85
Pol	VRC4409	B	86.97
Pol	HXB2	B	87.69

	B_Con	B	81.67
	HXB2	B	77.02
Vpr	B_Anc	B	85.85
	B_Con	B	83.24
	HXB2	B	73.83
Vpu	B_Anc	B	67.18
	B_Con	B	66.96
	HXB2	B	43.82

Pol	B_Anc	B	89.00
Pol	B_Con	B	89.30
