# Supplementary material for: Sieve analysis of breakthrough HIV-1 sequences in HVTN 505 identifies vaccine pressure targeting the CD4 binding site of Env-gp120
Source: PLoS One. 2017 Nov 17;12(11):e0185959. doi: 10.1371/journal.pone.0185959 (PMC5693417; doi:10.1371/journal.pone.0185959)
Supplement: S10 Table — Epitopes predicted to be strong and weak binders were matched against vaccine inserts or HIV-1 reference sequences and the predicted binding affinity of breakthrough virus-derived epitopes were compared to those of vaccine- and reference-derived epitopes. The distribution of summary values determined for each subject was compared between vaccine and placebo groups using Mann-Whitney tests. (PDF) [file pone.0185959.s010.pdf]

Table S10. Comparison of binding affinity measures for predicted CTL epitopes from vaccine and placebo recipients. Epitopes predicted to be strong and weak binders were matched against vaccine inserts or HIV-1 reference sequences and the predicted binding affinity of breakthrough virus-derived epitopes were compared to those of vaccine- and reference-derived epitopes. The distribution of summary values determined for each subject was compared between vaccine and placebo groups using Mann-Whitney tests.

BINDING AFFINITY - STRONG AND WEAK BINDERS

Env-gp120

	VRC-A		VRC-B		VRC-C		Cons.B		Anc.B		HXB2	
	Vaccine	Placebo	Vaccine	Placebo	Vaccine	Placebo	Vaccine	Placebo	Vaccine	Placebo	Vaccine	Placebo
n	25	18	25	18	25	18	25	18	25	18	25	18
Median	1.117	1.311	1.179	1.413	1.198	1.220	1.215	1.182	1.488	1.279	1.211	1.326
Mean	1.261	1.537	1.208	1.396	1.404	1.317	1.349	1.418	1.630	1.913	1.223	1.417
P value	0.056		0.118		0.966		0.813		0.809		0.234	

Env-gp41

	VRC-A		VRC-B		VRC-C		Cons.B		Anc.B		HXB2	
	Vaccine	Placebo	Vaccine	Placebo	Vaccine	Placebo	Vaccine	Placebo	Vaccine	Placebo	Vaccine	Placebo
n	25	18	25	18	25	18	25	18	25	18	25	18
Median	0.980	1.468	1.320	1.605	1.198	1.100	1.570	1.206	1.389	1.196	1.330	1.339
Mean	1.260	1.810	1.456	1.634	2.054	2.159	1.519	1.431	1.391	1.305	1.403	1.379
P value	**0.019**		0.345		0.981		0.150		0.319		0.753	

Gag

	VRC-B		Cons.B		Anc.B		HXB2	
	Vaccine	Placebo	Vaccine	Placebo	Vaccine	Placebo	Vaccine	Placebo
n	24	18	24	18	24	18	24	18
Median	1.114	1.115	1.096	1.089	1.097	1.062	1.098	1.083
Mean	1.253	1.132	1.225	1.110	1.208	1.077	1.248	1.113
P value	0.367		0.452		0.228		0.452	

Pol

	VRC-B		Cons.B		Anc.B		HXB2	
	Vaccine	Placebo	Vaccine	Placebo	Vaccine	Placebo	Vaccine	Placebo
n	24	18	24	18	24	18	24	18
Median	1.137	1.173	1.099	1.107	1.136	1.110	1.104	1.099
Mean	1.194	1.242	1.162	1.193	1.171	1.192	1.183	1.218
P value	0.249		0.693		0.862		0.905	

Nef

	VRC-B		Cons.B		Anc.B		HXB2	
	Vaccine	Placebo	Vaccine	Placebo	Vaccine	Placebo	Vaccine	Placebo
**n**	25	18	25	18	25	18	25	18
**Median**	1.339	1.099	1.174	1.010	1.354	1.088	1.364	1.138
**Mean**	1.730	1.585	1.509	1.278	1.670	1.282	1.747	1.573
**P value**	0.560		**0.049**		**0.039**		0.497	

Rev

	Cons.B		Anc.B		HXB2	
	Vaccine	Placebo	Vaccine	Placebo	Vaccine	Placebo
**n**	25	16	25	17	25	16
**Median**	1.578	1.508	1.632	1.531	1.250	1.173
**Mean**	3.241	2.082	2.974	2.151	3.372	2.161
**P value**	0.922		0.934		0.752	

Tat

	Cons.B		Anc.B		HXB2	
	Vaccine	Placebo	Vaccine	Placebo	Vaccine	Placebo
**n**	17	9	17	9	17	9
**Median**	1.000	1.000	1.000	1.000	1.446	1.362
**Mean**	1.240	1.036	1.424	1.163	1.760	1.286
**P value**	0.676		0.735		0.269	

Vif

	Cons.B		Anc.B		HXB2	
	Vaccine	Placebo	Vaccine	Placebo	Vaccine	Placebo
**n**	24	18	24	18	24	18
**Median**	1.130	1.129	1.141	1.126	1.365	1.199
**Mean**	1.679	1.371	1.656	1.353	1.792	1.435
**P value**	0.965		0.822		0.269	

Vpr

	Cons.B		Anc.B		HXB2	
	Vaccine	Placebo	Vaccine	Placebo	Vaccine	Placebo
**n**	24	18	24	18	24	18
**Median**	1.061	1.002	1.101	1.002	0.994	0.993
**Mean**	1.171	1.114	1.226	1.113	1.078	1.107
**P value**	0.315		0.191		0.921	

Vpu

	Cons.B		Anc.B		HXB2	
	Vaccine	Placebo	Vaccine	Placebo	Vaccine	Placebo
**n**	25	18	25	18	25	18
**Median**	1.312	1.186	1.291	1.149	1.112	1.080
**Mean**	2.101	2.414	2.080	3.022	3.698	1.219
**P value**	0.295		0.645		0.476	
