# Supplementary material for: Sieve analysis of breakthrough HIV-1 sequences in HVTN 505 identifies vaccine pressure targeting the CD4 binding site of Env-gp120
Source: PLoS One. 2017 Nov 17;12(11):e0185959. doi: 10.1371/journal.pone.0185959 (PMC5693417; doi:10.1371/journal.pone.0185959)
Supplement: S11 Table — Epitopes predicted to be strong and weak binders were matched against vaccine inserts or HIV-1 reference sequences and evolutionary distances were computed between breakthrough virus-derived epitopes and vaccine or reference-derived epitopes. The distribution of summary values determined for each subject was compared between vaccine and placebo groups using Mann-Whitney tests. (PDF) [file pone.0185959.s011.pdf]

Table S11. Comparison of evolutionary distances for predicted CTL epitopes from vaccine and placebo recipients. Epitopes predicted to be strong and weak binders were matched against vaccine inserts or HIV-1 reference sequences and evolutionary distances were computed between breakthrough virus-derived epitopes and vaccine or reference-derived epitopes. The distribution of summary values determined for each subject was compared between vaccine and placebo groups using Mann-Whitney tests.

EVOLUTIONARY DISTANCES - STRONG AND WEAK BINDERS

Env-gp120

	VRC-A		VRC-B		VRC-C		Cons.B		Anc.B		HXB2	
	Vaccine	Placebo	Vaccine	Placebo	Vaccine	Placebo	Vaccine	Placebo	Vaccine	Placebo	Vaccine	Placebo
n	25	18	25	18	25	18	25	18	25	18	25	18
Median	0.253	0.229	0.170	0.176	0.236	0.253	0.142	0.154	0.147	0.149	0.155	0.155
Mean	0.244	0.233	0.179	0.185	0.237	0.255	0.146	0.144	0.155	0.150	0.161	0.158
P value	0.295		0.580		0.206		0.832		0.716		0.716	

Env-gp41

	VRC-A		VRC-B		VRC-C		Cons.B		Anc.B		HXB2	
	Vaccine	Placebo	Vaccine	Placebo	Vaccine	Placebo	Vaccine	Placebo	Vaccine	Placebo	Vaccine	Placebo
n	25	18	25	18	25	18	25	18	25	18	25	18
Median	0.230	0.282	0.228	0.229	0.296	0.220	0.160	0.165	0.172	0.156	0.185	0.167
Mean	0.247	0.297	0.223	0.228	0.277	0.226	0.164	0.169	0.158	0.157	0.177	0.180
P value	0.066		> 0.999		**0.014**		0.889		0.771		0.885	

Gag

	VRC-B		Cons.B		Anc.B		HXB2	
	Vaccine	Placebo	Vaccine	Placebo	Vaccine	Placebo	Vaccine	Placebo
n	24	18	24	18	24	18	24	18
Median	0.109	0.101	0.091	0.099	0.100	0.118	0.110	0.100
Mean	0.108	0.109	0.097	0.101	0.099	0.114	0.115	0.110
P value	0.925		0.807		0.091		0.746	

Pol

	VRC-B		Cons.B		Anc.B		HXB2	
	Vaccine	Placebo	Vaccine	Placebo	Vaccine	Placebo	Vaccine	Placebo
n	24	18	24	18	24	18	24	18
Median	0.087	0.073	0.068	0.061	0.071	0.068	0.082	0.067
Mean	0.087	0.077	0.070	0.064	0.072	0.067	0.085	0.071
P value	0.090		0.137		0.158		**0.016**	

Nef

	VRC-B		Cons.B		Anc.B		HXB2	
	Vaccine	Placebo	Vaccine	Placebo	Vaccine	Placebo	Vaccine	Placebo
**n**	25	18	25	18	25	18	25	18
**Median**	0.231	0.216	0.125	0.125	0.147	0.124	0.260	0.226
**Mean**	0.238	0.206	0.137	0.138	0.163	0.137	0.258	0.219
**P value**	0.123		0.885		0.188		0.069	

Rev

	Cons.B		Anc.B		HXB2	
	Vaccine	Placebo	Vaccine	Placebo	Vaccine	Placebo
**n**	25	16	25	17	25	16
**Median**	0.231	0.226	0.232	0.218	0.341	0.258
**Mean**	0.262	0.242	0.257	0.231	0.306	0.306
**P value**	0.580		0.505		0.874	

Tat

	Cons.B		Anc.B		HXB2	
	Vaccine	Placebo	Vaccine	Placebo	Vaccine	Placebo
**n**	17	9	17	9	17	9
**Median**	0.100	0.000	0.024	0.000	0.283	0.202
**Mean**	0.102	0.076	0.099	0.091	0.261	0.228
**P value**	0.557		0.452		0.301	

Vif

	Cons.B		Anc.B		HXB2	
	Vaccine	Placebo	Vaccine	Placebo	Vaccine	Placebo
**n**	24	18	24	18	24	18
**Median**	0.118	0.143	0.129	0.140	0.144	0.145
**Mean**	0.127	0.131	0.127	0.129	0.143	0.146
**P value**	0.768		0.768		0.885	

Vpr

	Cons.B		Anc.B		HXB2	
	Vaccine	Placebo	Vaccine	Placebo	Vaccine	Placebo
**n**	24	18	24	18	24	18
**Median**	0.102	0.083	0.112	0.087	0.125	0.091
**Mean**	0.119	0.084	0.124	0.096	0.131	0.098
**P value**	**0.037**		0.209		**0.040**	

Vpu

	Cons.B		Anc.B		HXB2	
	Vaccine	Placebo	Vaccine	Placebo	Vaccine	Placebo
**n**	25	18	25	18	25	18
**Median**	0.231	0.232	0.228	0.227	0.323	0.210
**Mean**	0.230	0.197	0.229	0.225	0.299	0.218
**P value**	0.528		0.847		0.057	
