# Supplementary material for: Sieve analysis of breakthrough HIV-1 sequences in HVTN 505 identifies vaccine pressure targeting the CD4 binding site of Env-gp120
Source: PLoS One. 2017 Nov 17;12(11):e0185959. doi: 10.1371/journal.pone.0185959 (PMC5693417; doi:10.1371/journal.pone.0185959)
Supplement: S12 Table — Epitopes predicted to be strong binders were matched against vaccine inserts or reference sequences and the predicted binding affinity of breakthrough virus-derived epitopes were compared to those of vaccine- or reference-derived epitopes. The distribution of summary values determined for each subject was compared between vaccine and placebo groups using Mann-Whitney tests. (PDF) [file pone.0185959.s012.pdf]

Table S12. Comparison of binding affinity measures for predicted CTL epitopes (strong binders only) from vaccine and placebo recipients.

Epitopes predicted to be strong binders were matched against vaccine inserts or reference sequences and the predicted binding affinity of breakthrough virus-derived epitopes were compared to those of vaccine- or reference-derived epitopes. The distribution of summary values determined for each subject was compared between vaccine and placebo groups using Mann-Whitney tests.

BINDING AFFINITY - STRONG BINDERS

Env-gp120

	VRC-A		VRC-B		VRC-C		Cons.B		Anc.B		HXB2	
	Vaccine	Placebo	Vaccine	Placebo	Vaccine	Placebo	Vaccine	Placebo	Vaccine	Placebo	Vaccine	Placebo
n	25	18	25	18	25	18	25	18	25	18	25	18
Median	0.758	0.770	0.860	0.840	0.862	0.965	0.956	0.909	0.960	1.035	0.868	0.901
Mean	0.805	0.868	0.881	1.018	0.890	1.016	0.941	1.051	1.049	1.181	0.896	1.037
P value	0.516		0.947		0.345		0.735		0.398		0.966	

Env-gp41

	VRC-A		VRC-B		VRC-C		Cons.B		Anc.B		HXB2	
	Vaccine	Placebo	Vaccine	Placebo	Vaccine	Placebo	Vaccine	Placebo	Vaccine	Placebo	Vaccine	Placebo
n	25	18	25	18	24	18	25	18	25	18	25	18
Median	0.692	0.774	0.840	0.937	0.938	0.875	0.904	0.986	0.905	0.991	0.875	0.939
Mean	0.708	0.854	0.880	1.008	0.923	0.904	0.953	1.020	0.939	1.053	0.902	0.977
P value	0.350		0.485		0.875		0.455		0.275		0.265	

Gag

	VRC-B		Cons.B		Anc.B		HXB2	
	Vaccine	Placebo	Vaccine	Placebo	Vaccine	Placebo	Vaccine	Placebo
n	24	18	24	18	24	18	24	18
Median	1.025	1.030	0.998	0.998	0.916	0.969	0.990	0.996
Mean	1.108	1.078	1.075	1.044	1.038	0.995	1.078	1.035
P value	0.826		0.693		0.620		> 0.999	

Pol

	VRC-B		Cons.B		Anc.B		HXB2	
	Vaccine	Placebo	Vaccine	Placebo	Vaccine	Placebo	Vaccine	Placebo
n	24	18	24	18	24	18	24	18
Median	0.934	0.942	0.943	0.967	0.951	0.969	0.970	0.954
Mean	0.953	0.984	0.960	0.991	0.965	0.993	0.977	0.985
P value	0.885		> 0.999		0.945		0.565	

Nef

	VRC-B		Cons.B		Anc.B		HXB2	
	Vaccine	Placebo	Vaccine	Placebo	Vaccine	Placebo	Vaccine	Placebo
**n**	24	18	24	17	24	18	24	17
**Median**	0.801	0.993	1.000	0.895	1.022	0.940	0.793	0.982
**Mean**	0.876	0.947	1.006	0.872	1.127	0.875	0.867	0.926
**P value**	0.283		**0.006**		**0.004**		0.394	

Rev

	Cons.B		Anc.B		HXB2	
	Vaccine	Placebo	Vaccine	Placebo	Vaccine	Placebo
**n**	18	8	18	8	17	7
**Median**	0.635	0.708	0.510	0.817	0.723	0.937
**Mean**	0.777	1.730	0.828	1.871	0.893	1.236
**P value**	0.196		0.126		0.126	

Tat

	Cons.B		Anc.B		HXB2	
	Vaccine	Placebo	Vaccine	Placebo	Vaccine	Placebo
**n**	3	2	3	2	2	2
**Median**	1.000	0.557	1.000	1.000	1.801	0.695
**Mean**	0.684	0.557	0.823	1.000	1.801	0.695
**P value**	> 0.999		> 0.999		0.667	

Vif

	Cons.B		Anc.B		HXB2	
	Vaccine	Placebo	Vaccine	Placebo	Vaccine	Placebo
**n**	24	18	24	18	24	18
**Median**	0.839	0.919	0.835	0.924	0.930	0.912
**Mean**	0.847	0.927	0.809	0.926	0.906	0.957
**P value**	0.383		0.125		0.469	

Vpr

	Cons.B		Anc.B		HXB2	
	Vaccine	Placebo	Vaccine	Placebo	Vaccine	Placebo
**n**	22	16	22	16	22	16
**Median**	0.821	0.855	1.004	0.954	0.856	0.849
**Mean**	0.963	0.833	1.161	0.965	0.955	0.828
**P value**	0.844		0.685		0.731	

Vpu

	Cons.B		Anc.B		HXB2	
	Vaccine	Placebo	Vaccine	Placebo	Vaccine	Placebo
**n**	22	14	21	14	16	9
**Median**	0.615	0.942	0.572	0.806	0.481	0.894
**Mean**	0.877	0.841	0.847	0.720	0.819	0.905
**P value**	0.516		0.727		0.427	
