# Supplementary material for: Sieve analysis of breakthrough HIV-1 sequences in HVTN 505 identifies vaccine pressure targeting the CD4 binding site of Env-gp120
Source: PLoS One. 2017 Nov 17;12(11):e0185959. doi: 10.1371/journal.pone.0185959 (PMC5693417; doi:10.1371/journal.pone.0185959)
Supplement: S13 Table — Epitopes predicted to be strong binders were matched against vaccine inserts or HIV-1 reference sequences and evolutionary distances were computed between breakthrough virus-derived epitopes and vaccine- or reference-derived epitopes. The distribution of summary values determined for each subject was compared between vaccine and placebo groups using Mann-Whitney tests. (PDF) [file pone.0185959.s013.pdf]

Table S13. Comparison of evolutionary distances for predicted CTL epitopes (strong binders only) from vaccine and placebo recipients.

Epitopes predicted to be strong binders were matched against vaccine inserts or HIV-1 reference sequences and evolutionary distances were computed between breakthrough virus-derived epitopes and vaccine- or reference-derived epitopes. The distribution of summary values determined for each subject was compared between vaccine and placebo groups using Mann-Whitney tests.

EVOLUTIONARY DISTANCES - STRONG BINDERS

Env-gp120

	VRC-A		VRC-B		VRC-C		Cons.B		Anc.B		HXB2	
	Vaccine	Placebo	Vaccine	Placebo	Vaccine	Placebo	Vaccine	Placebo	Vaccine	Placebo	Vaccine	Placebo
**n**	25	18	25	18	25	18	25	18	25	18	25	18
**Median**	0.262	0.259	0.210	0.210	0.270	0.251	0.197	0.173	0.191	0.170	0.228	0.197
**Mean**	0.273	0.249	0.221	0.223	0.275	0.271	0.194	0.172	0.195	0.167	0.219	0.204
**P value**	0.356		0.966		0.809		0.382		0.233		0.466	

Env-gp41

	VRC-A		VRC-B		VRC-C		Cons.B		Anc.B		HXB2	
	Vaccine	Placebo	Vaccine	Placebo	Vaccine	Placebo	Vaccine	Placebo	Vaccine	Placebo	Vaccine	Placebo
**n**	25	18	25	18	24	18	25	18	25	18	25	18
**Median**	0.270	0.254	0.194	0.207	0.271	0.216	0.156	0.174	0.167	0.167	0.188	0.183
**Mean**	0.266	0.278	0.252	0.212	0.250	0.224	0.160	0.175	0.163	0.169	0.189	0.190
**P value**	0.860		0.692		0.567		0.500		0.908		0.885	

Gag

	VRC-B		Cons.B		Anc.B		HXB2	
	Vaccine	Placebo	Vaccine	Placebo	Vaccine	Placebo	Vaccine	Placebo
**n**	24	18	24	18	24	18	24	18
**Median**	0.139	0.133	0.113	0.119	0.141	0.146	0.161	0.133
**Mean**	0.142	0.170	0.128	0.148	0.141	0.151	0.157	0.172
**P value**	0.730		0.674		0.885		0.862	

Pol

	VRC-B		Cons.B		Anc.B		HXB2	
	Vaccine	Placebo	Vaccine	Placebo	Vaccine	Placebo	Vaccine	Placebo
**n**	24	18	24	18	24	18	24	18
**Median**	0.104	0.077	0.073	0.070	0.079	0.069	0.081	0.072
**Mean**	0.104	0.088	0.077	0.075	0.083	0.080	0.087	0.078
**P value**	0.061		0.862		0.708		0.532	

Nef

	VRC-B		Cons.B		Anc.B		HXB2	
	Vaccine	Placebo	Vaccine	Placebo	Vaccine	Placebo	Vaccine	Placebo
**n**	24	18	24	17	24	18	24	17
**Median**	0.269	0.222	0.146	0.126	0.181	0.128	0.275	0.229
**Mean**	0.259	0.209	0.145	0.144	0.172	0.151	0.268	0.216
**P value**	0.137		0.922		0.985		0.149	

Rev

	Cons.B		Anc.B		HXB2	
	Vaccine	Placebo	Vaccine	Placebo	Vaccine	Placebo
**n**	18	8	18	8	17	7
**Median**	0.280	0.319	0.301	0.312	0.365	0.408
**Mean**	0.281	0.336	0.291	0.324	0.336	0.376
**P value**	0.495		0.523		0.619	

Tat

	Cons.B		Anc.B		HXB2	
	Vaccine	Placebo	Vaccine	Placebo	Vaccine	Placebo
**n**	3	2	3	2	2	2
**Median**	0.000	0.080	0.000	0.000	0.203	0.338
**Mean**	0.139	0.080	0.131	0.000	0.203	0.338
**P value**	> 0.999		> 0.999		> 0.999	

Vif

	Cons.B		Anc.B		HXB2	
	Vaccine	Placebo	Vaccine	Placebo	Vaccine	Placebo
**n**	24	18	24	18	24	18
**Median**	0.136	0.103	0.150	0.103	0.121	0.102
**Mean**	0.144	0.114	0.151	0.115	0.133	0.112
**P value**	0.315		0.182		0.533	

Vpr

	Cons.B		Anc.B		HXB2	
	Vaccine	Placebo	Vaccine	Placebo	Vaccine	Placebo
**n**	22	16	22	16	22	16
**Median**	0.212	0.203	0.219	0.233	0.216	0.230
**Mean**	0.211	0.215	0.215	0.244	0.231	0.228
**P value**	0.820		0.372		0.843	

Vpu

	Cons.B		Anc.B		HXB2	
	Vaccine	Placebo	Vaccine	Placebo	Vaccine	Placebo
**n**	22	14	21	14	16	9
**Median**	0.273	0.253	0.310	0.284	0.365	0.222
**Mean**	0.292	0.260	0.285	0.299	0.361	0.296
**P value**	0.548		0.829		0.335	
