# Supplementary material for: Sieve analysis of breakthrough HIV-1 sequences in HVTN 505 identifies vaccine pressure targeting the CD4 binding site of Env-gp120
Source: PLoS One. 2017 Nov 17;12(11):e0185959. doi: 10.1371/journal.pone.0185959 (PMC5693417; doi:10.1371/journal.pone.0185959)
Supplement: S14 Table — Only epitopes that were identified in at least three vaccine and three placebo recipients were considered for comparison through Mann-Whitney tests. Mean values corresponding to all subjects in each group are reported; for a given subject, the binding affinity or evolutionary distance corresponds to the comparison of the breakthrough virus-derived epitope to the corresponding epitope in the subtype B vaccine. (PDF) [file pone.0185959.s014.pdf]

Table S14. Comparison between the vaccine and placebo groups for 19 Env-gp120 CTL epitopes. Only epitopes that were identified in at least three vaccine and three placebo recipients were considered for comparison through Mann-Whitney tests. Mean values corresponding to all subjects in each group are reported; for a given subject, the binding affinity or evolutionary distance corresponds to the comparison of the breakthrough virus-derived epitope to the corresponding epitope in the subtype B vaccine.

			**N. subjects**		**Binding affinity**			**Evolutionary distance**		
**Position**	**HLA**	**Epitope**	**Vaccine**	**Placebo**	**Vaccine**	**Placebo**	**p-value**	**Vaccine**	**Placebo**	**p-value**
18	A*02:01	TMLLGMLMI	15	6	0.720	0.672	0.953	0.167	0.213	0.775
**35**	**A*02:01**	**VTVYYGVPV**	**12**	**7**	**1.000**	**0.823**	**0.282**	**0.000**	**0.096**	**0.036**
66	A*02:01	NVWATHACV	12	5	1.149	1.000	> 0.999	0.007	0.000	> 0.999
102	A*02:01	QMHEDIISL	13	8	1.309	1.598	0.919	0.120	0.175	0.345
120	A*02:01	KLTPLCVSL	13	7	1.711	2.602	0.597	0.184	0.169	> 0.999
175	C*06:02	FYKLDIIPI	4	4	0.671	0.971	0.057	0.505	0.480	0.743
191	A*02:01	SLTSCNTSV	10	8	1.732	0.964	0.446	0.442	0.387	0.823
250	C*06:02	IRPVVSTQL	3	3	1.000	1.000	> 0.999	0.000	0.000	> 0.999
307	A*01:01	HIGPGRAFY	6	3	0.795	0.965	0.191	0.091	0.437	0.179
372	A*24:02	SFNCGGEFF	3	4	1.025	1.057	0.486	0.041	0.093	0.486
373	A*01:01	FNCGGEFFY	7	4	0.968	0.849	0.746	0.032	0.066	0.746
379	C*06:02	FFYCNSTQL	7	6	0.636	1.130	0.586	0.152	0.259	0.159
**379**	**C*07:01**	**FFYCNSTQL**	**11**	**4**	**0.817**	**0.427**	**0.018**	**0.259**	**0.288**	**0.686**
380	A*24:02	FYCNSTQLF	3	6	1.671	2.430	0.798	0.290	0.189	0.286
380	C*06:02	FYCNSTQLF	7	5	0.965	0.770	0.303	0.152	0.277	0.165
415	C*06:02	CRIKQIINM	5	4	0.980	1.241	0.651	0.111	0.313	0.389
442	A*01:01	CSSNITGLL	5	5	1.188	1.065	> 0.999	0.073	0.137	0.722
443	A*01:01	SSNITGLLL	4	3	1.000	1.000	> 0.999	0.000	0.000	> 0.999
472	C*06:02	MRDNWRSEL	4	6	1.000	1.083	> 0.999	0.000	0.030	> 0.999
