# Supplementary material for: Sieve analysis of breakthrough HIV-1 sequences in HVTN 505 identifies vaccine pressure targeting the CD4 binding site of Env-gp120
Source: PLoS One. 2017 Nov 17;12(11):e0185959. doi: 10.1371/journal.pone.0185959 (PMC5693417; doi:10.1371/journal.pone.0185959)
Supplement: S15 Table — Results of the mAb contact set scanning analysis grouped by monoclonal antibody class (CD4bs, CD4i, and V3, Quarternary, gp41 MPER, gp41 NHR, gp41 cluster II, and Glycan) and ranked within class by P-value. The effect size is measured in additional mismatch rate in the vaccine group per contact site where a positive (negative) value indicates more (fewer) mismatches at contact site residues in breakthrough sequences for the vaccine group as compared to the placebo group and n is the number of cantact sites. For results with a Q-value ≤ 0.2 the mAb name is in bold. (PDF) [file pone.0185959.s015.pdf]

Table S15. Monoclonal antibody contact set scanning.

Results of the mAb contact set scanning analysis grouped by monoclonal antibody class (CD4bs, CD4i, and V3, Quarternary, gp41 MPER, gp41 NHR, gp41 cluster II, and Glycan) and ranked within class by P-value. The effect size is measured in additional mismatch rate in the vaccine group per contact site where a positive (negative) value indicates more (fewer) mismatches at contact site residues in breakthrough sequences for the vaccine group as compared to the placebo group and **n** is the number of cantact sites. For results with a Q-value ≤ 0.2 the mAb name is in bold.

	mAb	Effect	n	P-value	Q-value
**CD4bs**	**CD4**	4.9%	48	0.00079	0.035
	**F105**	5.2%	36	0.00289	0.039
	**b12**	5.8%	32	0.00311	0.039
	**VRC-PG20**	4.2%	44	0.00431	0.039
	**12A12**	3.9%	47	0.00448	0.039
	**NIH45-46**	3.5%	48	0.01209	0.072
	**VRC03**	3.7%	43	0.01257	0.072
	**b13**	5.9%	26	0.01479	0.072
	**CH103**	5.1%	27	0.01642	0.072
	**VRC06**	3.2%	46	0.02000	0.078
	**3BNC117**	3.6%	39	0.02210	0.078
	**VRC23**	3.5%	43	0.02298	0.078
	**VRC-PG04**	3.9%	32	0.02881	0.082
	**VRC01**	3.8%	37	0.02933	0.082
	**CH31**	4.4%	31	0.03382	0.086
**CD4i**	**412d**	2.0%	37	0.03514	0.086
	**X5**	2.5%	25	0.06689	0.155
	17b	2.0%	19	0.11406	0.251
	48d	1.7%	23	0.13082	0.274
	21c	1.9%	25	0.22526	0.411
**V3**	**PGT128**	5.3%	12	0.01483	0.072
	**PGT122**	4.9%	15	0.02997	0.082
	4025	4.0%	14	0.21402	0.411
	537-10D	3.9%	13	0.21777	0.411
	2558	3.3%	15	0.23380	0.411
	R20	3.2%	9	0.25528	0.432
	1006-15D	3.2%	13	0.30330	0.440
	2219	2.9%	13	0.30920	0.440
	268-D	3.2%	11	0.30943	0.440
	3074	3.3%	14	0.30967	0.440
	R56	2.1%	10	0.44015	0.605
	F425-B4e8	2.8%	11	0.46506	0.620
	447-52D	2.1%	9	0.54235	0.645
	PGT135	1.3%	19	0.59721	0.661
**Quaternary**	PG16	1.1%	13	0.72926	0.783
	PG9	0.2%	17	0.92635	0.948
**gp41 MPER**	10E8	2.0%	14	0.30750	0.440
	4E10	1.5%	10	0.52193	0.638
	Z13e1	1.8%	8	0.52193	0.638
	2F5	-2.0%	7	0.58217	0.661
	m66	0.1%	13	0.96003	0.960
**gp41 NHR**	D5	-0.9%	24	0.52192	0.638
**gp41 cluster II**	1281	0.5%	16	0.84981	0.890
**Glycan**	2G12	2.0%	6	0.60059	0.661
