# Supplementary material for: Sieve analysis of breakthrough HIV-1 sequences in HVTN 505 identifies vaccine pressure targeting the CD4 binding site of Env-gp120
Source: PLoS One. 2017 Nov 17;12(11):e0185959. doi: 10.1371/journal.pone.0185959 (PMC5693417; doi:10.1371/journal.pone.0185959)

Figure S1: Mindist sequence cysteine counts. (A) Cysteine counts of gp160 mindist sequences by treatment group. P-values compare counts by treatment using a two-sample t-test. (B) A total of twenty cysteines in gp160 were found in all mindist sequences consisting of 9 pairs in gp120 and 1 pair in gp41. At least one additional cysteine was found in each of the mindist sequences in one or more of the following regions; gp41 cytoplasmic tail (CT), gp41 transmembrane domain (TM), the V1 variable loop (V1), and the signal peptide (SP). Each additional cysteine is represented by a bar of height one color coded by region. When additional cysteine residues were observed they came as a pair in V1 (2 vaccine participant mindist sequences, blue bars), either a pair or singleton in CT and SP (red and purple bars), and as a singleton in TM (2 vaccine participant mindist sequences, green bars).

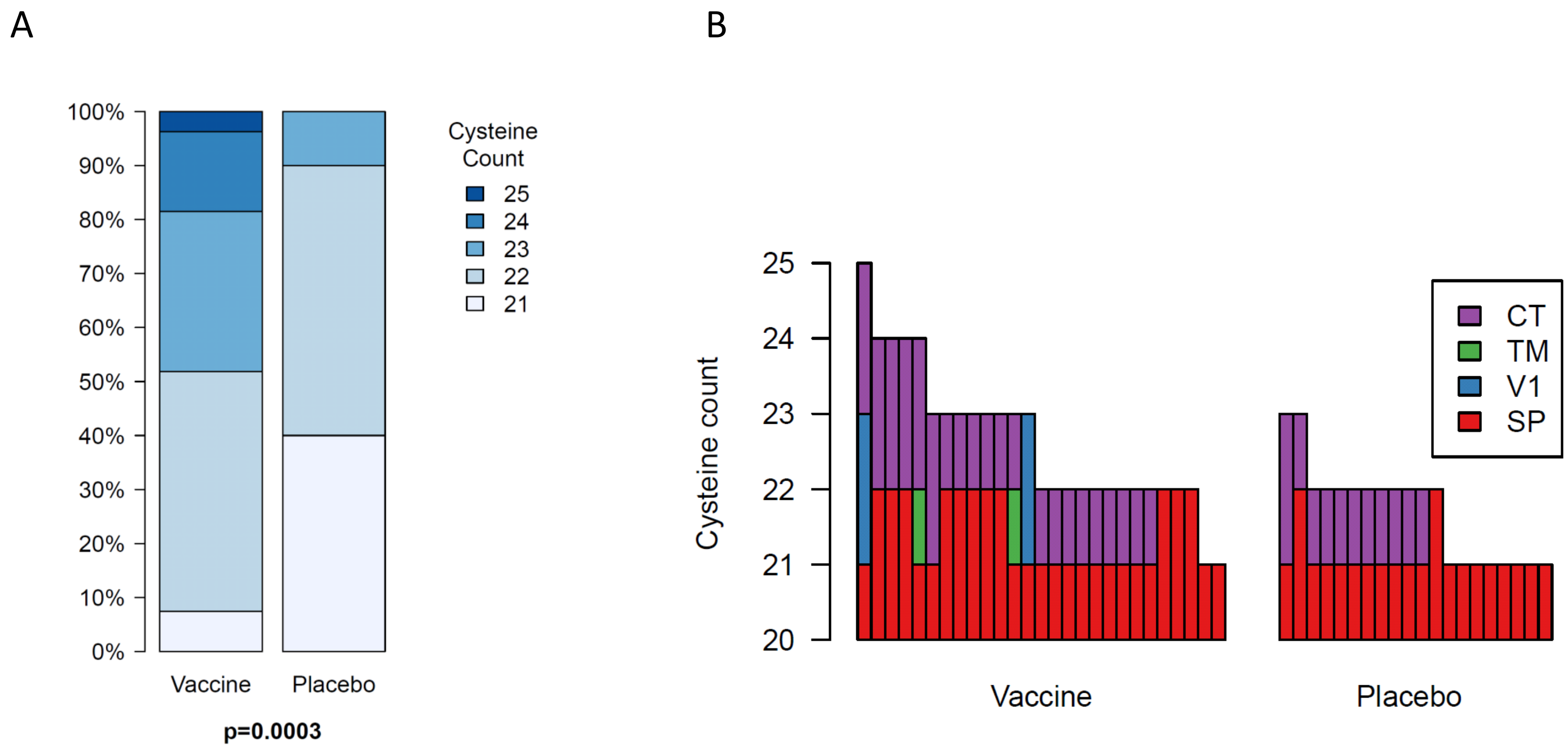

Supplement: S1 Fig — (A) Cysteine counts of gp160 mindist sequences by treatment group. P-values compare counts by treatment using a two-sample t-test. (B) A total of twenty cysteines in gp160 were found in all mindist sequences consisting of 9 pairs in gp120 and 1 pair in gp41. At least one additional cysteine was found in each of the mindist sequences in one or more of the following regions; gp41 cytoplasmic tail (CT), gp41 transmembrane domain (TM), the V1 variable loop (V1), and the signal peptide (SP). Each additional cysteine is represented by a bar of height one color coded by region. When additional cysteine residues were observed they came as a pair in V1 (2 vaccine participant mindist sequences, blue bars), either a pair or singleton in CT and SP (red and purple bars), and as a singleton in TM (2 vaccine participant mindist sequences, green bars). (PDF) [file pone.0185959.s018.pdf]
