# Supplementary material for: Sieve analysis of breakthrough HIV-1 sequences in HVTN 505 identifies vaccine pressure targeting the CD4 binding site of Env-gp120
Source: PLoS One. 2017 Nov 17;12(11):e0185959. doi: 10.1371/journal.pone.0185959 (PMC5693417; doi:10.1371/journal.pone.0185959)

Figure S4: The amino acid (AA) distribution for linear signature region Env-gp120 AA192-207 by treatment group for all breakthrough sequences. Each row displays the AA distribution for one site relative to the VRC-B vaccine strain AA. Sequences for each participant are represented by a bar of equal height with the reference sequence AA residue, in black, shown above the midline. Within a bar, colors depict the fraction of the participant's sequences with that AA residue (or insertion or deletion, indicated by a dash). The widths of the bars scaled so that the total width for each treatment group is the same. The Y-axis label indicates the HXB2 position.

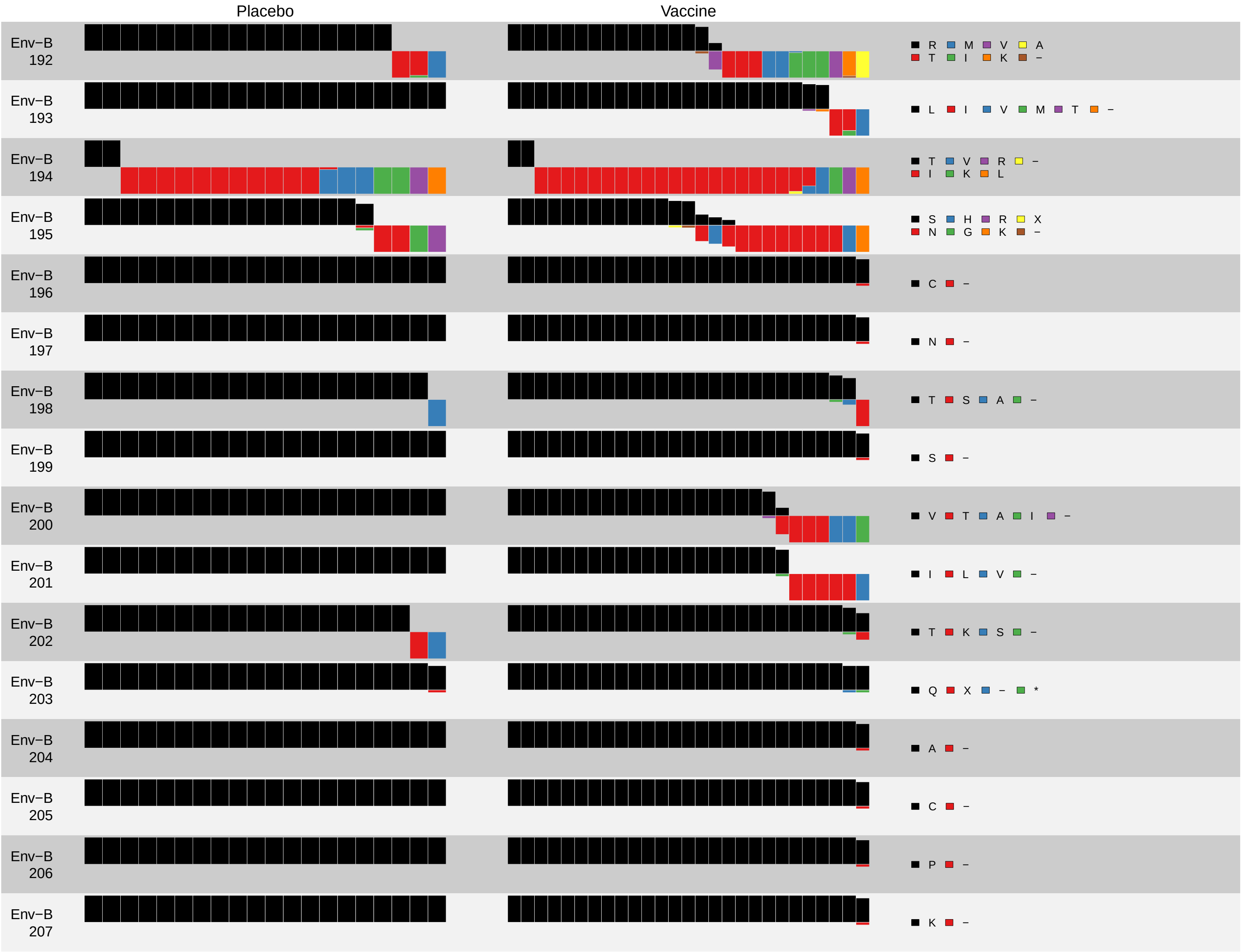

Supplement: S4 Fig — The amino acid (AA) distribution for linear signature region Env-gp120 AA192-207 by treatment group for all breakthrough sequences. Each row displays the AA distribution for one site relative to the VRC-B vaccine strain AA. Sequences for each participant are represented by a bar of equal height with the reference sequence AA residue, in black, shown above the midline. Within a bar, colors depict the fraction of the participant's sequences with that AA residue (or insertion or deletion, indicated by a dash). The widths of the bars are scaled so that the total width for each treatment group is the same. The Y-axis label indicates the HXB2 position. (PDF) [file pone.0185959.s021.pdf]
