# Supplementary material for: Sieve analysis of breakthrough HIV-1 sequences in HVTN 505 identifies vaccine pressure targeting the CD4 binding site of Env-gp120
Source: PLoS One. 2017 Nov 17;12(11):e0185959. doi: 10.1371/journal.pone.0185959 (PMC5693417; doi:10.1371/journal.pone.0185959)

Figure S8: Percent Epitope Mismatch based on (A) netMHCpan and (B) ADT. For Env, predicted weak binders to all 9-mers from the three vaccine insert sequences (VRC-A, VRC-B, and VRC-C) combined were used to determine the percent epitope mismatch. Similarly, percent epitope mismatch was computed using all of the non-insert genes of HIV-1 combined. Mismatch percent for vaccine recipients in red and placebo recipients in blue. P-values are from a choplump Wilcoxon test.

A

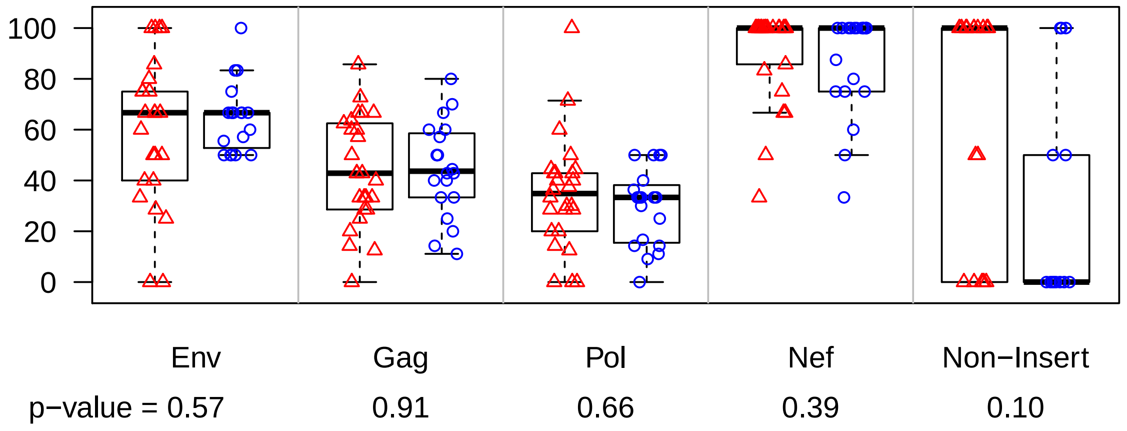

B

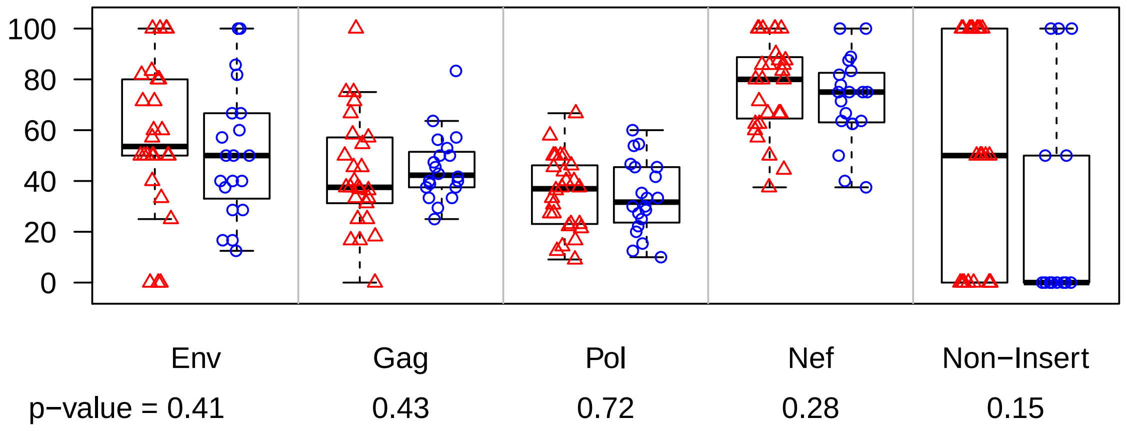

Supplement: S8 Fig — Percent epitope mismatch based on (A) netMHCpan and (B) ADT. For Env, predicted weak binders to all 9-mers from the three vaccine insert sequences (VRC-A, VRC-B, and VRC-C) combined were used to determine the percent epitope mismatch. Similarly, percent epitope mismatch was computed using all of the non-insert genes of HIV-1 combined. Mismatch percent for vaccine recipients in red and placebo recipients in blue. P-values are from a choplump Wilcoxon test. (PDF) [file pone.0185959.s025.pdf]
