# Supplementary material for: Sieve analysis of breakthrough HIV-1 sequences in HVTN 505 identifies vaccine pressure targeting the CD4 binding site of Env-gp120
Source: PLoS One. 2017 Nov 17;12(11):e0185959. doi: 10.1371/journal.pone.0185959 (PMC5693417; doi:10.1371/journal.pone.0185959)

Fig S9. Antibody-dependent cellular phagocytosis (ADCP) activity of antibodies induced by the DNA/rAd5 vaccine regimen at (A) baseline and (B) 4 weeks post-4th vaccination. Neutravidin fluorescent beads were coated with a biotinylated HIV-1 antigen (ConSgp140), then incubated with monoclonal antibodies (positive control CH31 and negative control CH65) or IgG purified from participant serum samples. THP-1 cells (pre-treated with anti-human CD4 to reduce CD4-Env mediated virus internalization) were incubated with the antibody/bead mixture, then paraformaldehyde-fixed before analysis by flow cytometry. A phagocytic score was determined based on the ratio of experimental sample to PBS control. For further details, see the Supplementary Methods. Data from responders are shown in red and non-responders in blue, with box plots based on data from responders superimposed on the distribution. The mid-line of the box denotes the median and the ends of the box denote the 25th and 75th percentiles. The whiskers that extend from the top and bottom of the box extend to the most extreme data points that are no more than 1.5 times the interquartile range (i.e., height of the box) or if no value meets this criterion, to the data extremes.

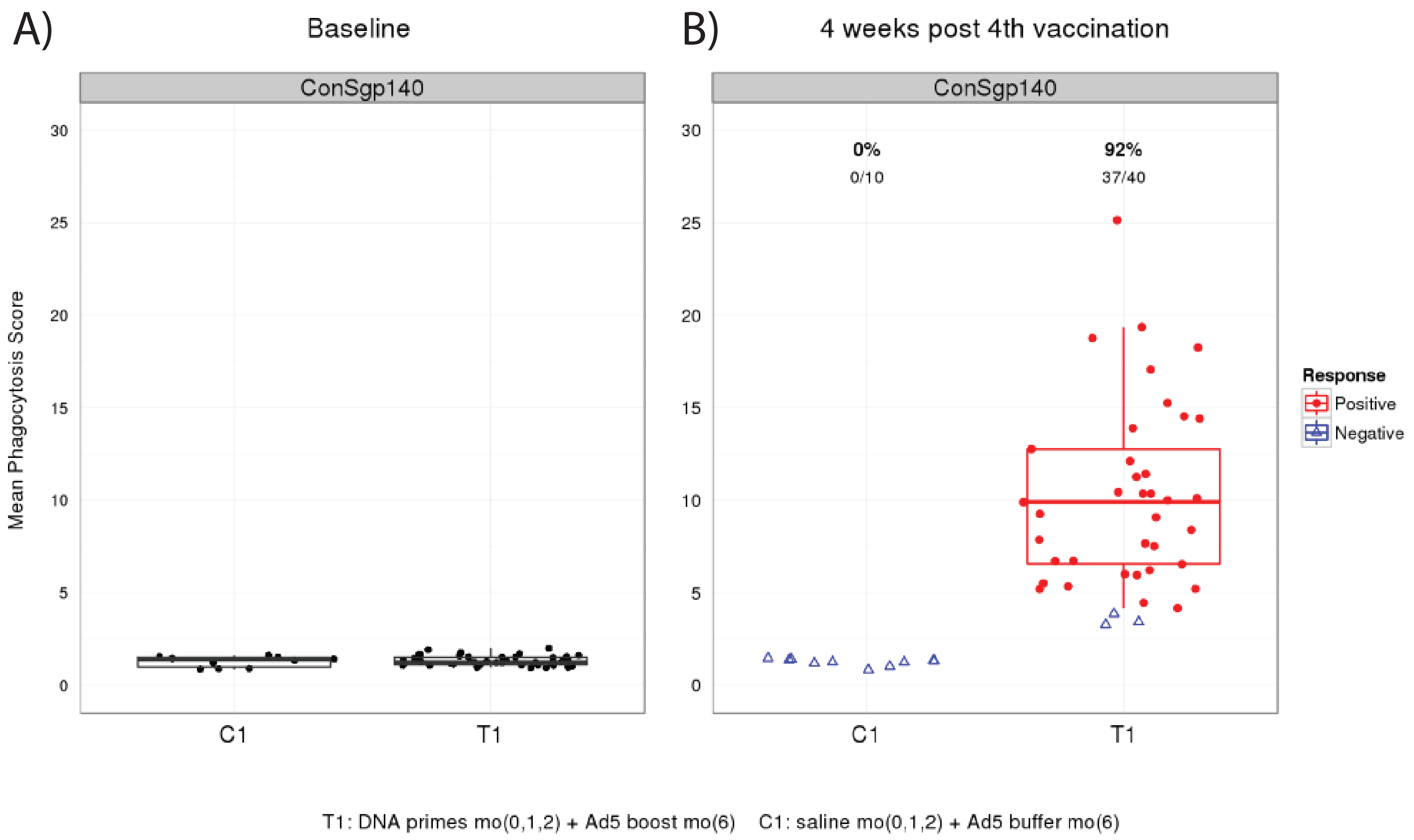

Supplement: S9 Fig — Antibody-dependent cellular phagocytosis (ADCP) activity of antibodies induced by the DNA/rAd5 vaccine regimen at (A) baseline and (B) 4 weeks post-4th vaccination. Neutravidin fluorescent beads were coated with a biotinylated HIV-1 antigen (ConSgp140), then incubated with monoclonal antibodies (positive control CH31 and negative control CH65) or IgG purified from participant serum samples. THP-1 cells (pre-treated with anti-human CD4 to reduce CD4-Env mediated virus internalization) were incubated with the antibody/bead mixture, then paraformaldehyde-fixed before analysis by flow cytometry. A phagocytic score was determined based on the ratio of experimental sample to PBS control. For further details, see S1 Supplementary Methods. Data from responders are shown in red and non-responders in blue, with box plots based on data from responders superimposed on the distribution. The mid-line of the box denotes the median and the ends of the box denote the 25th and 75th percentiles. The whiskers that extend from the top and bottom of the box extend to the most extreme data points that are no more than 1.5 times the interquartile range (i.e., height of the box) or if no value meets this criterion, to the data extremes. (PDF) [file pone.0185959.s026.pdf]
