# Supplementary material for: Sieve analysis of breakthrough HIV-1 sequences in HVTN 505 identifies vaccine pressure targeting the CD4 binding site of Env-gp120
Source: PLoS One. 2017 Nov 17;12(11):e0185959. doi: 10.1371/journal.pone.0185959 (PMC5693417; doi:10.1371/journal.pone.0185959)
Supplement: S1 Supplementary Methods — (DOCX) [file pone.0185959.s027.docx]

**Supplementary Methods**

for

“Sieve Analysis of Breakthrough HIV-1 Sequences in HVTN 505

Identifies Vaccine Pressure Targeting the CD4 Binding Site of Env-gp120”

by deCamp and Rolland et al.

Table of Contents

Supplementary Methods 2

HIV-1 sequences 2

Vaccine sequences 2

Reference sequences 2

Sequence analysis 2

Evaluation of evidence for positive selection 2

Identification of potential N-linked glycosylation sites 2

Reconstruction of maximum-likelihood phylogenetic trees 3

CTL epitope predictions 3

Site scanning methods 4

GWJ, EGWJ, MBS, PCP, and QEMD methods 4

PF test statistic 5

Specific sets of AA for site scanning 5

Machine learning highly multivariable sieve analysis 6

Antibody-dependent cellular phagocytosis (ADCP) 7

Participants analyzed 7

Endpoints 7

Laboratory methods 7

Statistical analysis 8

References for Supplementary Methods 9

## Supplementary Methods

### HIV-1 sequences

#### Vaccine sequences

The vaccine insert sequences were as follows: *gag*, subtype B strain HXB2 (K03455); *pol* and *nef*, subtype B strain NL4-3 (M19921)]. The subtype A, B, and C *env* sequences were from 92RW020 (CCR5-tropic, U08794), HXB2 (CXCR4-tropic, K03455), and 97ZA012 (CCR5-tropic, AF286227), respectively.

#### Reference sequences

The HIV-1 subtype B consensus 2004 (B.Con), ancestral (B.Anc), and subtype B HXB2 (K03455) sequences served as additional references.

### Sequence analysis

#### Evaluation of evidence for positive selection

Evidence of positive selection was examined at each codon separately for the vaccine and placebo groups by measuring the nonsynonymous (dN) minus synonymous substitution rates (dS), dN-dS [1] using SLAC, a fixed effects likelihood (IFEL) method and MEME, under the GTR nucleotide substitution model as implemented in HyPhy [2]. Sites with dN-dS significantly greater than 0 (1-sided p < 0.025) were considered to be under positive selection.

#### Identification of potential N-linked glycosylation sites and Co-receptor specificity

Potential N-linked glycosylation sites (PNLGS) were identified from either *mindist* or all sequences were compared between treatment groups as described previously [3]. CCR5 and CXCR4 Co-receptor usage was predicted via the Web PSSM [4] (<http://indra.mullins.microbiol.washington.edu/webpssm/>) using the X4R5 matrix for HIV-1 subtype B.

#### Reconstruction of maximum-likelihood phylogenetic trees

Maximum-likelihood phylogenetic trees were reconstructed using PhyML [version 3.0]) [5] implemented in DIVEIN [6] (<http://indra.mullins.microbiol.washington.edu/cgi-bin/DIVEIN>). Pairwise and tree-based distances were calculated between participant-derived sequences and HIV-1 reference sequences (vaccine inserts or subtype B central sequences) using the General Time Reversible nucleotide substitution model (GTR + I + G) or the HIVb model of protein evolution [7]. Trees were also reconstructed for Env-gp120 after removing amino acid positions from variable segments in the signal peptide 10-16; V1 loop 132-152; V2 loop 186-191; V3 loop 309-310; V4 loop 392-413; and V5 loop 460-464 (HXB2 positions). These measures were summarized as a single value (mean) per participant and compared between the vaccine and placebo groups using Mann-Whitney tests with exact 2-sided p-values.

Analyses of both near full-length genomes and individual genes were conducted to estimate the number of founder variants. Note that as 10 genomes per participant represents a small fraction of the viral population, there was a 65% probability of observing a variant that corresponded to only 10% of the viral population. Alignments and trees were visually inspected; the timing of the most recent common ancestor [using BEAST [8] and Poisson-fitter [9]] was estimated; phylogenetically-informative (shared in at least two sequences) and private mutations were compared [6].

### CTL epitope predictions

CTL epitope predictions were obtained using NetMHCpan version 2.8 [10] (<http://www.cbs.dtu.dk/services/NetMHCpan/>) and adaptive double threading (ADT) [11]. Unique HLA-peptide combinations (9-mers only) that qualified as strong or weak binders based on their predicted binding affinity were used in analyses (strong binders <50 nM; weak binders <500 nM). A participant-derived predicted epitope was considered matched to the vaccine/reference epitope when both 9-mer peptides were identical or differed by a maximum of 3 residues. Matched epitopes were compared by calculating both the difference in predicted binding affinity between the two peptides and the evolutionary distance between them; evolutionary distances were computed using the HIVb matrix [7]. Summary values corresponding to participant-specific mean binding affinity or mean evolutionary distance were calculated for each participant and compared using Mann-Whitney tests. Predicted epitopes in Env-gp120 were analyzed on an epitope-by-epitope basis when an epitope was identified in at least three vaccine and three placebo recipients.

Percent epitope mismatch distances [12] to the reference sequence were compared between treatment groups using choplump Wilcoxon tests [13]. For each breakthrough infection, the this distance between was computed as one minus the percentage of predicted epitopes in the reference sequence that were identical to the corresponding epitope in all of the breakthrough sequences, with predicted epitopes defined as weak binders. Two analyses were done, for weak binders defined by NetMHCpan or ADT.

### Site scanning methods

#### GWJ, EGWJ, MBS, PCP, and QEMD methods

At each site, the AA distribution was compared across treatment groups using the Gilbert, Wu, Jobes (GWJ) [14] method, which uses a single representative sequence per participant, and the Expected Gilbert, Wu, Jobes (EGWJ) method, which uses all the participant’s sequences [3]; Numeric weights signify a match (1) or mismatch (0) between the AA in the breakthrough sequence and the AA in the vaccine insert [3, 14]. For EGWJ, the match/mismatch weight corresponding to a particular participant at a particular site is the expected value over the frequency distribution of AAs for that participant/site. GWJ and EGWJ compute 2-sided p-values by randomly permuting vaccine/placebo labels of participants 10,000 times, which preserves covariation of sequences. The Model-Based Sieve (MBS) method calculates the posterior probability that the AA distribution of breakthrough infections differs between treatment groups, and computes a 2-sided p-value via 10,000 permutations [15]. The Physico-Chemical Property (PCP) method compares counts of Taylor properties at each AA site between each breakthrough sequence residue and the insert residue [16]. This analysis was restricted to 4 of 10 properties, namely hydrophobicity, polarity, charge and small steric volume, with the analysis conducted separately for each of the 4 properties; 2-sided p-values were computed with a two-sample t-test. The Quasi-Earth Mover’s Distance (QEMD) method examines whether the pairwise similarity scores between vaccine and placebo sequences are different from what would be expected under the null hypothesis that they came from the same distribution. This approach does not use a reference sequence. QEMD was computed using Hamming distance as the summary measure of similarity and a 2-sided p-value computed by 10,000 permutations. [3].

#### PF test statistic

To compute the PF test statistic, each *mindist* sequence was first converted into a vector of indicator variables defined by the presence or absence of one AA or gap at a given site. Each vector has a length of 21 times the number of sites. Next, these vectors were averaged by treatment group resulting in a vector of frequencies of each AA or gap at each position for each group. Finally, the PF statistic was defined as the Euclidean distance between these two frequency vectors. Mid p-values [17] based on 10,000 permutations were used to test the null hypothesis that the distance equals zero.

#### Specific sets of AA for site scanning

Sets of known mAb contact sites were defined for 44 known interactions between a ligand and a mAb or CD4 [3] and corresponded to the CD4bs, CD4i, V3, Glycan, Quarternary, Env-gp41 N-terminal heptad-repeat (NHR), Env-gp41 immunodominant cluster II and Env-gp41 membrane proximal external region (MPER) (S17 Table). Sets corresponding to peptide microarray hotspots were defined based on the results of microarrays conducted on Month 0 and 7 plasma from a pilot immunogenicity study of randomly sampled HIV-1 uninfected participants (40 vaccine and 10 placebo recipients). Peptide microarrays covered Env-gp160 consensus sequences for HIV-1 Group M, subtypes A, B, C, D, CRF01_AE and CRF02_AG with 1423 peptides (15-mers overlapping by 12 amino acids). Antibody reactivity hotspots were defined via the method of Imholte et al. [18] based on a response rate threshold of 30%. Two hotspots were identified; one in the V3 loop (HXB2 positions AA304-318) and another in the cluster I immunodominant region in Env-gp41 (HXB2 positions AA581-593) [19].

Signature sites of interest from prior sieve analyses of HIV-1 vaccine efficacy trials included ten sites identified in the Step Study (Gag 84, 211, 403, 475; Pol 541 and 721; Nef 64a, 82, 116, 173), and five sites from RV144 (Pol 41; Env 169, 181, 307 and 317). These corresponded to published results with a sieve effect Q-value less than or equal to 0.2, or sites with a P-value below 0.05 and with follow-up analyses providing supportive experimental data [3, 20].

### Machine learning sieve analysis

The feature space of each analysis was defined to include the following binary information at every AA position: the presence or absence of a residue; match or mismatch to the protein-relevant insert (using multiple inserts when necessary); match or mismatch to the population consensus of all of the HVTN 505 sequences; and, the presence or absence of one of ten physicochemical properties, as defined by Taylor [16]. This resulted in a high-dimensional data set that was reduced using the same site-specific filters as for the local sieve analysis, as well as a multicollinearity filter, where features with correlated data were grouped, and all but one of the correlated features were removed. The multicollinearity threshold was adjusted independently for each analysis to achieve a final dimensionality of the feature space (p/n) of less than 5.

Learning methods were chosen based on their facility of working with small datasets with a large feature space. The methods chosen were LASSO, Naïve Bayes, Random Forests, and boosted decision stumps as an ensemble of weak learners [21-24]. Each analysis was performed using 1,000 iterations of Monte Carlo cross validation (MCCV), with a training split of 65% and the remaining 35% for validation (appropriate to the size of the data set). The summary statistics of classification performance were the cross-validated area under the ROC curve (AUC) and classification accuracy, taken as the mean of the results on validation set data over all MCCV iterations. A level of signal was determined to be of interest if its cross-validated mean AUC was above 0.7.

### Antibody-dependent cellular phagocytosis (ADCP)

#### Participants analyzed

To assay ADCP activity in antibodies elicited by the DNA/rAd5 vaccine regimen in HVTN 505, a subset of 50 trial participants (40 vaccinees and 10 placebo recipients) was selected. Participants were randomly selected from study participants that met all of the following criteria: had received all 4 study vaccinations within the appropriate window; had plasma, serum, and PBMC samples available from all four immunogenicity visits; and had completed the month 24 visit without evidence of HIV infection.

#### Endpoints

The frequency and magnitude mean phagocytosis score responses were measured in serum samples from these 50 participants obtained at visit 7 (visit day 196), corresponding to 4 weeks post-final vaccination in treatment arms T1 and C1. Baseline samples were also assayed for positivity determination at visit 2 (visit day 0), corresponding to enrollment.

#### Laboratory methods

To assess the ability of antibodies induced by the DNA/rAd5 vaccine regimen to engage FcR, antibody-dependent cellular phagocytosis (ADCP) was measured as described [25, 26]. Briefly, neutravidin fluorescent beads were coated with a biotinylated HIV-1 antigen (ConSgp140), then incubated with monoclonal antibodies (positive control CH31 and negative control CH65) or IgG purified from participant serum samples. For the ADCP assay, THP-1 cells (pre-treated with anti-human CD4 to reduce CD4-Env mediated virus internalization) were incubated with the antibody/bead mixture, then paraformaldehyde-fixed before analysis by flow cytometry. A phagocytic score was determined based on the ratio of experimental sample to PBS control. Mean phagocytosis score is defined as: (% positive cells x MFI positive cells for participant IgG) / (% positive x MFI positive for no-IgG control).

#### Statistical analysis of ADCP

Samples from post-enrollment visits were declared to have positive responses if both of the following criteria were met.

1. Mean phagocytosis score at follow-up > positivity threshold
2. Mean phagocytosis score at follow-up > 3x mean phagocytosis score at baseline

The positivity threshold is defined separately for each protocol and antigen as the 95th percentile of the mean phagocytosis score of baseline samples. Response rates and corresponding 95% confidence intervals were calculated by the Wilson score method [27]. Mean phagocytosis score was used to summarize the magnitude at a given time-point.

## References for Supplementary Methods

1. Gojobori T, Yamaguchi Y, Ikeo K, Mizokami M. Evolution of pathogenic viruses with special reference to the rates of synonymous and nonsynonymous substitutions. Jpn J Genet. 1994;69(5):481-8.

2. Pond SL, Frost SD, Muse SV. HyPhy: hypothesis testing using phylogenies. Bioinformatics. 2005;21(5):676-9.

3. Edlefsen PT, Rolland M, Hertz T, Tovanabutra S, Gartland AJ, deCamp AC, et al. Comprehensive sieve analysis of breakthrough HIV-1 sequences in the RV144 vaccine efficacy trial. PLoS Comput Biol. 2015;11(2):e1003973.

4. Jensen MA, Coetzer M, van 't Wout AB, Morris L, Mullins JI. A reliable phenotype predictor for human immunodeficiency virus type 1 subtype C based on envelope V3 sequences. J Virol. 2006;80(10):4698-704.

5. Guindon S, Gascuel O. A simple, fast, and accurate algorithm to estimate large phylogenies by maximum likelihood. Syst Biol. 2003;52(5):696-704.

6. Deng W, Maust BS, Nickle DC, Learn GH, Liu Y, Heath L, et al. DIVEIN: a web server to analyze phylogenies, sequence divergence, diversity, and informative sites. BioTechniques. 2010;48(5):405-8. Epub 2010/06/24.

7. Nickle DC, Heath L, Jensen MA, Gilbert PB, Mullins JI, Kosakovsky Pond SL. HIV-specific probabilistic models of protein evolution. PLoS One. 2007;2(6):e503.

8. Drummond AJ, Rambaut A. BEAST: Bayesian evolutionary analysis by sampling trees. BMC Evol Biol. 2007;7:214. Epub 2007/11/13.

9. Giorgi EE, Funkhouser B, Athreya G, Perelson AS, Korber BT, Bhattacharya T. Estimating time since infection in early homogeneous HIV-1 samples using a poisson model. BMC Bioinformatics. 2010;11:532. Epub 2010/10/27.

10. Nielsen M, Lundegaard C, Blicher T, Lamberth K, Harndahl M, Justesen S, et al. NetMHCpan, a method for quantitative predictions of peptide binding to any HLA-A and -B locus protein of known sequence. PLoS One. 2007;2(8):e796.

11. Jojic N, Reyes-Gomez M, Heckerman D, Kadie C, Schueler-Furman O. Learning MHC I--peptide binding. Bioinformatics. 2006;22(14):e227-35.

12. Rolland M, Tovanabutra S, deCamp AC, Frahm N, Gilbert PB, Sanders-Buell E, et al. Genetic impact of vaccination on breakthrough HIV-1 sequences from the STEP trial. Nat Med. 2011;17(3):366-71. Epub 2011/03/02.

13. Follmann D, Fay MP, Proschan M. Chop-lump tests for vaccine trials. Biometrics. 2009;65(3):885-93.

14. Gilbert PB, Wu C, Jobes DV. Genome scanning tests for comparing amino acid sequences between groups. Biometrics. 2008;64(1):198-207.

15. Rolland M, Edlefsen PT, Larsen BB, Tovanabutra S, Sanders-Buell E, Hertz T, et al. Increased HIV-1 vaccine efficacy against viruses with genetic signatures in Env V2. Nature. 2012;490(7420):417-20.

16. Taylor WR. The classification of amino acid conservation. J Theor Biol. 1986;119(2):205-18.

17. Routledge RD. Practicing Safe Statistics with the Mid-P. Canadian Journal of Statistics-Revue Canadienne De Statistique. 1994;22(1):103-10.

18. Imholte GC, Sauteraud R, Korber B, Bailer RT, Turk ET, Shen X, et al. A computational framework for the analysis of peptide microarray antibody binding data with application to HIV vaccine profiling. J Immunol Methods. 2013;395(1-2):1-13.

19. Xu JY GM, Palker T, Karwowska S, Zolla-Pazner S. Epitope mapping of two immunodominant domains of gp41, the transmembrane protein of human immunodeficiency virus type 1, using ten human monoclonal antibodies. J Virol. 1991;65(9).

20. Zolla-Pazner S, Edlefsen PT, Rolland M, Kong XP, deCamp A, Gottardo R, et al. Vaccine-induced Human Antibodies Specific for the Third Variable Region of HIV-1 gp120 Impose Immune Pressure on Infecting Viruses. EBioMedicine. 2014;1(1):37-45.

21. John GH, Langley P. Estimating continuous distributions in Bayesian classifiers. Proceedings of the Eleventh Conference on Uncertainty in Artificial Intelligence; Montreal, Quebec Canada. 2074196: Morgan Kaufmann Publishers Inc.; 1995. p. 338-45.

22. Freund YaS, Robert E. Experiments with a New Boosting Algorithm. Proceedings of the Thirteenth International Conference on Machine Learning (ICML 1996): Morgan Kaufmann; 1996. p. 148-56.

23. Breiman L. Random forests. Machine Learning. 2001;45(1):5-32.

24. Tibshirani R. Regression shrinkage and selection via the Lasso. Journal of the Royal Statistical Society Series B-Methodological. 1996;58(1):267-88.

25. Ackerman ME, Moldt B, Wyatt RT, Dugast AS, McAndrew E, Tsoukas S, et al. A robust, high-throughput assay to determine the phagocytic activity of clinical antibody samples. J Immunol Methods. 2011;366(1-2):8-19.

26. Tay MZ, Liu P, Williams LD, McRaven MD, Sawant S, Gurley TC, et al. Antibody-Mediated Internalization of Infectious HIV-1 Virions Differs among Antibody Isotypes and Subclasses. PLoS Pathog. 2016;12(8):e1005817.

27. Agresti A, Coull BA. Approximate is better than "exact" for interval estimation of binomial proportions. Am Stat. 1998;52(2):119-26.
